# Polydatin Inhibits Neuroinflammation in Cerebral Ischemia–Reperfusion Injury Through Suppressing the CXCL3/CXCR2 Axis

**DOI:** 10.1002/cns.70947

**Published:** 2026-05-23

**Authors:** Youxiang Cui, Fangmei Hu, Shixin Wang, Zehua Liu, Jinzhou Chen, Mingyue Cui, Leilei Wang, Yuming Wang, Ning Wang, Yuhong Bian, Shuquan Lv, Huantian Cui

**Affiliations:** ^1^ Cangzhou Hospital of Integrated Traditional Chinese Medicine and Western Medicine Cangzhou China; ^2^ Key Laboratory of Neurological Rehabilitation Cangzhou Hospital of Integrated Traditional Chinese Medicine and Western Medicine Cangzhou China; ^3^ Qingxian Hospital of Traditional Chinese Medicine Cangzhou China; ^4^ Huanghua Hospital of Traditional Chinese Medicine Huanghua China; ^5^ Nantong Hospital of Traditional Chinese Medicine Nantong China; ^6^ Tianjin University of Traditional Chinese Medicine Tianjin China; ^7^ Yunnan University of Chinese Medicine Kunming China

**Keywords:** cerebral ischemia–reperfusion injury, CXCL3/CXCR2 axis, microglial cell, Polydatin, Transcriptomics

## Abstract

**Aim:**

The aim of this study is to investigate the precise mechanisms by which Polydatin (PD) ameliorates cerebral ischemia–reperfusion injury (CIRI).

**Methods:**

We first established a rat model of CIRI to evaluate PD's therapeutic effects. Transcriptomic analysis was used to explore PD's impact on gene expression in ischemic brain tissue of CIRI rats. OGD/R‐induced microglial activation experiments validated that PD inhibits microglial activation by targeting the CXCL3/CXCR2 axis.

**Results:**

PD intervention improved neurological function scores of CIRI rats, reduced infarct area, alleviated pathological damage, and preserved Nissl bodies in brain tissue. It also modulated oxidative stress levels. Transcriptomic analysis showed enrichment of the Cytokine–cytokine receptor interaction pathway and several inflammatory pathways after PD intervention, with downregulation of CXCL3 and CXCR2. PD decreased pro‐inflammatory cytokine levels and CXCL3/CXCR2 protein expression in ischemic brain tissue of CIRI rats, and reduced positive expression areas of IBA1^+^CXCL3^+^, IBA1^+^CXCR2^+^, and MPO^+^CXCR2^+^. In vitro experiments demonstrated that PD reduced pro‐inflammatory cytokine levels in BV2 cell supernatants treated with OGD/R and inhibited BV2 cell migration. However, these effects were abolished when treated with CXCL3 neutralizing antibody and SiCXCR2.

**Conclusion:**

Our findings suggest that PD can inhibit microglial migration and activation by acting on the CXCL3/CXCR2 axis, thereby alleviating the inflammatory response in CIRI.

## Graphical abstract

PD can act on the CXCL3/CXCR2 axis, thereby inhibiting migration and activation of microglia and neutrophils, and thus alleviating the inflammatory response associated with CIRI, highlighting its potential as a therapeutic agent for CIRI.

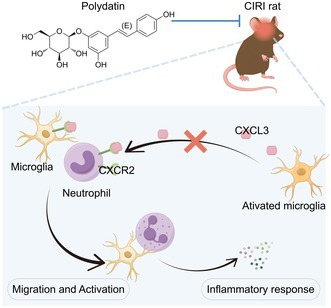

## 1 Introduction

Stroke stands as the second leading cause of death and disability globally, predominantly driven by ischemic stroke, which constitutes roughly 87% of all stroke occurrences [1]. This form of stroke is marked by heightened morbidity, disability rates, and mortality. In recent years, reperfusion therapy, utilizing thrombolysis or interventional treatments, has emerged as the primary intervention for ischemic stroke [2]. However, reperfusion of ischemic brain tissue can incite secondary damage termed cerebral ischemia–reperfusion injury (CIRI), significantly impacting patients' quality of life [3]. Despite strides in modern medicine concerning CIRI diagnosis and treatment, challenges persist, including postoperative recovery hurdles and adverse drug reactions. Hence, there is a pressing need for novel therapeutic avenues and drugs to address CIRI.

Inflammation assumes a pivotal role in exacerbating CIRI [4, 5, 6]. Notably elevated levels of proinflammatory cytokines such as TNF‐α, IL‐6, and IL‐1β are observed in the serum of CIRI patients compared to healthy individuals [7]. Similarly, these cytokines are markedly increased in the serum of CIRI animal models [8]. Microglia, specialized macrophages within brain tissue, demonstrate significant infiltration in the brain tissue of CIRI animal models [9]. Further investigations reveal that ischemia and hypoxia trigger microglial activation, prompting them to secrete proinflammatory cytokines, thereby inducing neuroinflammatory reactions and facilitating CIRI onset [10]. Suppression of the inflammatory response proves effective in ameliorating CIRI [4]. Notably, the activation of the CXCL3/CXCR2 signaling has been confirmed to play a crucial role in CIRI [11]. Microglia serve as both a source of CXCL3 [11, 12] and a cell type functionally expressing CXCR2 [13]. This autocrine/paracrine loop continuously amplifies neuroinflammatory responses, making it a key therapeutic target for CIRI. In recent years, research has found that natural products, including ginkgo terpene lactones, ginkgolide B, total alkaloids in Leonuri Herba, quercetin, and breviscapine, have demonstrated significant efficacy in treating cerebral ischemia–reperfusion injury (CIRI) primarily by modulating physiological functions and inhibiting neuroinflammation [14].

Polydatin (PD) possesses various properties, including anti‐inflammatory, antioxidant, anti‐apoptotic, and anticancer effects [15]. Previous studies have highlighted PD's efficacy in protecting against spinal ischemia–reperfusion injury [16] and protecting the central nervous system [17]. However, the precise mechanisms underlying PD's therapeutic effects on CIRI warrant further exploration. This study aims to elucidate the therapeutic mechanisms of PD in CIRI utilizing transcriptomics technology coupled with Transwell cell migration experiments, building upon a comprehensive understanding of its therapeutic potential in CIRI. Initially, we established a CIRI rat model to examine PD's ameliorative effects on CIRI. Subsequently, transcriptomics technology was used to assess PD's impact on gene expression in the brain tissue of CIRI rats. Building on the transcriptomic findings, we further validated PD's effect on the CXCL3/CXCR2 axis. Additionally, considering the pivotal role of CXCL3/CXCR2 axis regulation in microglial activation during the inflammatory response in CIRI, we conducted in vitro experiments using OGD/R to induce microglial activation and verified PD's mechanism of inhibiting microglial activation through its interaction with the CXCL3/CXCR2 axis. This study reveals that the neuroprotective effect of PD against CIRI is associated with the suppression of the CXCL3/CXCR2 axis in microglia, supporting its potential as a novel candidate for the treatment of CIRI.

## 2 Methods

### 2.1 In Vivo Experiment

#### 2.1.1 Animals and Reagents

According to epidemiological reports, adult men have a significantly higher incidence and risk of ischemic stroke than women [18, 19, 20]. Animal experiments similarly demonstrate that male SD rats have a higher incidence and severity of ischemic stroke compared to age‐matched females [21] even exhibiting higher mortality and elevated pro‐inflammatory cytokines [22]. Based on this clinical reality, we selected male rats for this study, an approach that is consistent with numerous previous investigations [23, 24, 25, 26].

We procured SD rats (SPF grade, healthy males, aged 6–8 weeks, weighing approximately 230 g) from SPF (Beijing) Biotechnology Co. Ltd., possessing an animal license number of SCXK (Beijing) 2019–0010. These rats were accommodated in cages housing five rats each and maintained under standard SPF‐grade conditions, with normal feeding. Approval for all animal experiments was obtained from the Ethical Review Committee of Animal Experiments in Yunnan University of Chinese Medicine (Approval No.: R‐062023LH300).

Polydatin (15,721, Empirical formula: C_20_H_22_O_8_, Purity ≥ 95%, Constitutional formula was shown in Figure S1) was purchased from Merck China (Beijing, China). Detailed information regarding the materials and reagents utilized in this experiment is provided in the Supporting Informations.

#### 2.1.2 Establishment of the CIRI Rat Model

Following one week of acclimation to the environment, the rats underwent a 12 h fasting period before modeling, with unrestricted water access. The rat model for middle cerebral artery occlusion reperfusion was induced utilizing the modified thread‐occlusion method [27]. In brief, we anesthetized the rats via pentobarbital sodium intraperitoneal injection (50 mg/kg). We also exposed the common carotid artery, external carotid artery, and internal carotid artery. A suture was then inserted from the common carotid artery into the internal carotid artery through the external carotid artery until reaching the middle cerebral artery. Following a 2 h insertion period, the suture was withdrawn to induce reperfusion injury. The Sham group underwent identical procedures, excluding ligation and suture insertion. We collected tissue samples as well as relevant indicators after 5 days of reperfusion.

#### 2.1.3 Grouping and Dosing Regimen

We randomly allocated ninety SD rats into six groups: Sham, CIRI, Ginaton (GNT), and three different doses of PD intervention groups, namely low‐dose PD (L‐PD), medium‐dose PD (M‐PD), and high‐dose PD (H‐PD). CIRI models were induced in all groups except the Sham group. Post‐modeling, the Sham and CIRI groups received 0.01 mL/g of normal saline via gavage, the GNT group received Ginaton at a dosage of 21.6 mg/kg via gavage [28], and the L‐PD, M‐PD, and H‐PD groups were administered PD at doses of 50, 100, and 200 mg/kg via gavage, respectively [29]. Rats were subjected to daily gavage for a continuous period of 5 days.

#### 2.1.4 Neurological Function Evaluation

We assessed the rats' neurological function impairment using Bederson's score [30, 31], postural reflex test [32], and asymmetry score [33]. Bederson's score gauged the extent of body flexion; the postural reflex test determined body flexion when rats were suspended upside down; and the asymmetry score measured motor asymmetry by observing the frequency of the rats' left and right limbs touching the edge of the table. Detailed evaluation criteria are provided in Table S1.

#### 2.1.5 TTC Staining

As per previous literature, we measured the brain's infarct size [34]. In brief, the rat brain underwent division into six consecutive coronal sections, followed by staining with 2% triphenyltetrazolium chloride (TTC) in a dark environment at 37.0°C for 15 min. Subsequently, each section underwent photography, and the cerebral infarction area was quantified utilizing Image J.

#### 2.1.6 Pathological Staining

We fixed rat cerebral cortex tissue from the same ischemic region with 4% paraformaldehyde, followed by dehydration with ethanol and embedding in paraffin. Then, we prepared 5 μm sections. Hematoxylin–eosin (HE) [35] and Nissl [36] staining procedures were conducted following established protocols. The results of HE and Nissl staining were examined under an optical microscope and quantified using Image J.

Denatured cell index (DCI) was used for quantification of pathological changes in HE staining. Briefly, six non‐overlapping images from brain tissue staining section were obtained using a microscope (magnification: 400×). The numbers of total cells and denatured cells were calculated from each image. The average of total cells and denatured cell numbers from each section was calculated. The DCI was calculated as the number of denatured cells divided by the total cell count [37].

For Nissl body quantification, the total region of interest (ROI) was outlined manually (six sections per animal), and the percentage of Nissl‐positive area within each ROI was calculated relative to the total cytoplasmic area. Then, the optical density of Nissl staining was measured after subtracting background signals from adjacent white matter. Averaged optical densities were acquired in images with converted 8‐bit grayscale.

#### 2.1.7 Detection of SOD, MDA, and ROS Levels in Brain Tissue

We collected cerebral cortex tissue from the ischemic side and homogenized the tissue. The total protein concentration of the samples was normalized using the Bicinchoninic acid (BCA) method. Subsequently, we measured superoxide dismutase (SOD) activity, malondialdehyde (MDA), and reactive oxygen species (ROS) levels in the brain tissue according to the instructions provided in the kit.

#### 2.1.8 Transcriptome Analysis

We conducted transcriptomic analysis on the cerebral cortex tissue from the ischemic side of CIRI rats following established protocols [38]. In brief, we lysed and disrupted rat brain tissue using magnetic beads, followed by reverse transcription and purification of cDNA to establish an amplification library. Subsequently, we conducted Illumina sequencing to generate raw data. Quantification of gene expression levels was then carried out, followed by differential expression analysis and KEGG pathway enrichment analysis.

#### 2.1.9 Immunofluorescence Assay

We processed the ischemic cerebral cortex tissue from each group by dewaxing, hydrating, and conducting antigen retrieval. Following this, we subjected the sections to incubation at room temperature with a 3% hydrogen peroxide solution. Subsequently, the sections were treated at 4.0°C with 20% normal goat serum for 1 h, followed by overnight incubation with the primary antibodies: rabbit anti‐Iba1 (1/100) and mouse anti‐CXCL3 (1/1000), rabbit anti‐Iba1 (1/100) and mouse anti‐CXCR2 (1/500), mouse anti‐MPO (1/50) and rabbit anti‐CXCR2 (1/100) at 4.0°C After washing, the sections underwent incubation at 25.0°C with fluorescein‐conjugated secondary antibodies for 1 h. DAPI solution and anti‐fade mounting medium was then used to stain the cell nuclei. Capturing of six non‐overlapping images from brain tissue staining section was performed using a fluorescence microscope, and Pearson's correlation coefficient was calculated to quantify the expression of fluorophore co‐localization using Image J.

### 2.2 In Vitro Experiment

#### 2.2.1 Cell Culture

We cultured mouse microglial cells (BV2) in a complete medium consisting of 90% H‐DMEM, 10% FBS, and penicillin–streptomycin (P/S) solution. The cells were kept in a 37°C, 5% CO_2_ incubator and were passaged once they reached 75%–85% confluence.

HL‐60 cells were cultured in RPMI 1640 medium supplemented with 10% FBS and 1% P/S at 37°C with 5% CO_2_. We induced differentiation into neutrophil‐like cells by treating with 1.3% DMSO for 5 days, refreshing the medium every 3 days.

#### 2.2.2 Cell Transfection

Upon plating the cells, we allowed them to adhere to the plate. Following this, we discarded the medium and cultured the cells in serum‐free conditions for 2 h. Subsequently, we replaced the medium with fresh medium containing Lipo2000 transfection reagent, siCXCR2 plasmid, and an empty vector (NC) for a 6 h incubation period. The sequence of siCXCR2 is: CTCAAAGATGGGAGAATTCAAGG.

#### 2.2.3 MTT Assay

We treated plated BV2 cells and neutrophils with different concentrations of PD (0, 12.5, 25, 50, 100, 200 μM) for 24 h. Following this, we added 10 μL of MTT solution (5 mg/mL) to each well. After a 4 h incubation period, we discarded the medium and added 100 μL of DMSO. Subsequently, we measured the absorbance of the samples at 490 nm and calculated the relative cell viability based on absorbance values.

#### 2.2.4 Oxygen–Glucose Deprivation/Restoration (OGD/R) Cell Model

We established the oxygen–glucose deprivation/reperfusion (OGD/R) cell model based on previous reports to mimic CIRI in vitro [39]. Initially, BV2 cells in the logarithmic growth phase were harvested after adhering to the plate. Sugar‐free medium was introduced, and the cells were placed in a hypoxic chamber. To induce hypoxia, nitrogen gas was introduced into the chamber for 10 min to remove oxygen. Subsequently, the chamber was sealed and placed in an incubator for 2 h. Following this period, the cells were removed from the chamber and oxygen supply was resumed. The sugar‐free medium was replaced with complete medium, and an equivalent volume of cell culture medium was added. The cells were then placed in an incubator with a composition of 95% air and 5% CO_2_ for further culture for 24 h. Drug intervention was conducted after the oxygen–glucose deprivation of BV2 cells. Cells in the control group were continuously cultured in complete medium in a 37°C incubator with 95% air and 5% CO_2_.

#### 2.2.5 Transwell

To evaluate the effects of PD on BV2 cell migration, we seeded 1 × 10^5^ BV2 cells in the lower chamber of the Transwell system, followed by 24 h of OGD/R or PD intervention. Normal BV2 cells, BV2 cells treated with CXCL3 neutralizing antibody, or those transfected with SiCXCR2 were seeded in the upper chamber of the Transwell, at a density of 1 × 10^5^ cells per well. After an additional 24 h of culturing, we collected the supernatant of the co‐culture system to measure the levels of proinflammatory cytokines. To evaluate the effects of PD on CXCL3‐induced neutrophils migration, we added serum‐free medium containing 10 ng/mL CXCL3 to the lower chamber of the Transwell. 2 × 10^5^ neutrophils were seeded into the upper chamber and incubated for 3 h, with or without simultaneous treatment with PD. After incubation, we removed the upper chamber of the Transwell, discarded the cells on its inner side, and fixed the remaining cells with 4% paraformaldehyde for 10 min. Afterward, we stained the cells with 0.1% crystal violet for 20 min. The staining outcomes were observed under a microscope, and the number of stained cells in 5 randomly selected fields at 100× magnification was counted.

#### 2.2.6 Proinflammatory Cytokine Level Detection

In the in vivo experiments, we collected and homogenized the brain tissue on the ischemic side. The total protein concentration of the samples was normalized using the BCA method. For the in vitro experiments, we collected the cell supernatants and measured the levels of IL‐1β, IL‐6, and TNF‐α in both brain tissue and cell supernatants using ELISA kits.

#### 2.2.7 RT‐qPCR

A total RNA extraction kit was used to extract the total RNA from the cells cultured in vitro. We then measured the RNA concentration. Subsequently, we obtained cDNA using a reverse transcription kit. To determine the mRNA expression levels of target genes, we utilized qPCR. We calculated the relative expression levels of each target mRNA relative to β‐actin using the 2^−ΔΔCT^ method. Refer to the Supporting Informations for the primer sequences (Table S2).

#### 2.2.8 Western Blot Assay

Ultrasonic homogenization was used to extract total protein from the ischemic brain tissue and cells. We then determined the protein concentration through the BCA method. Following this, the proteins underwent heat‐denaturation and were separated by SDS‐PAGE before being transferred to a PVDF membrane. Subsequently, we blocked the membrane for 1 h with 5% skim milk. Primary antibodies against CXCL3, CXCR2, and GAPDH were then incubated overnight at 4.0°C. After washing, incubation was done at room temperature for 1 h with secondary antibodies. We quantitatively analyzed the ECL‐developed blots using Image J.

#### 2.2.9 Statistical Analysis

We conducted statistical analysis using SPSS Pro, expressing all data as mean ± SD. All data were tested for normality using the Shapiro–Wilk test. Parametric data were analyzed for group differences using one‐way ANOVA, followed by Tukey's post hoc test. Non‐parametric data were analyzed using the Kruskal–Wallis test, followed by Dunn's post hoc test. A significance level of *p* < 0.05 was deemed statistically significant.

## 3 Results

### 3.1 PD Has Significant Therapeutic Effects on CIRI Rats

**FIGURE 1:**
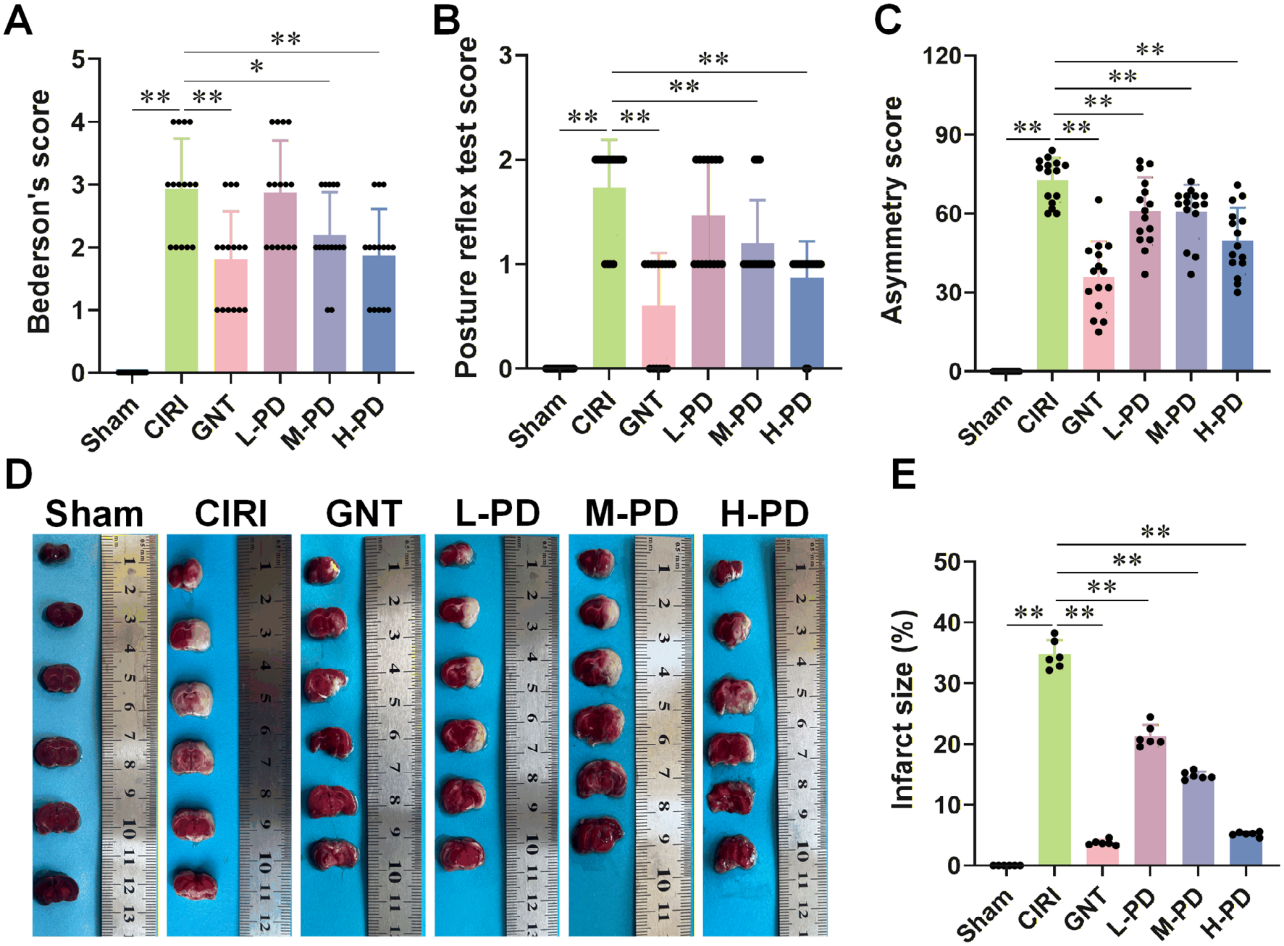
PD intervention significantly improves the neurological function and reduces the area of cerebral infarction in CIRI rats. CIRI models were established in rats through MCAO/R. PD intervention followed. The effects of PD on the neurological function and infarct area in CIRI rats were evaluated through neurological function scoring (Bederson's score, postural reflex score, and asymmetry score) and TTC staining of brain tissue. (A–C) PD intervention significantly reduced the Bederson's score (A), postural reflex score (B), and asymmetry score (C) in CIRI rats. (D, E) TTC staining revealed that PD intervention reduced the infarct area in CIRI rats. *n* = 15 for A‐C, *n* = 6 for D, E. Data are presented as the mean ± SD. *p < 0.05, **p < 0.01.

Initially, we assessed the neurological recovery in CIRI rats. Based on Bederson's score and the posture reflex test, CIRI rats displayed significantly higher neurological impairment scores compared to the Sham group. However, PD intervention notably mitigated neurological impairment in CIRI rats (Figure 1A,B). Moreover, PD significantly reduced the asymmetry score, suggesting its potential to enhance limb activity and foster neurological function recovery in CIRI rats (Figure 1C). Additionally, TTC staining unveiled a considerable increase in the cerebral infarction area in CIRI rats compared to the Sham group, which was notably diminished following 5 days of PD intervention (Figure 1D,E).

**FIGURE 2:**
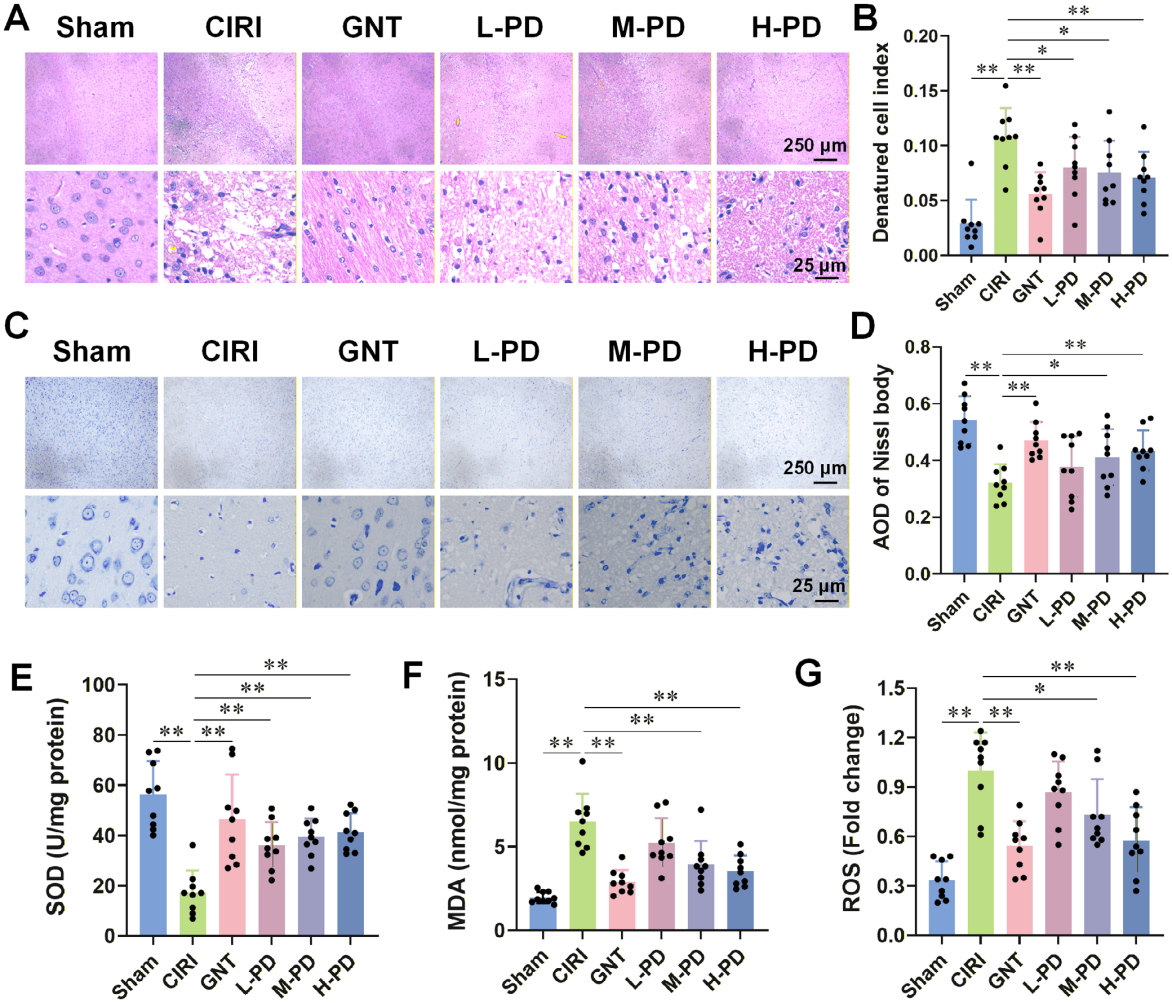
PD intervention significantly improves the pathological state and oxidative stress level in the brain tissue of CIRI rats. Using HE and Nissl staining, we observed the effect of PD on the pathological changes in ischemic areas of CIRI rat brain tissue. We evaluated the impact of PD on oxidative stress injury by measuring the levels of SOD, MDA, and ROS in the ischemic brain tissue. (A, B) HE staining showed that PD intervention improved the pathological state of the brain tissue and reduced the DCI index in CIRI rats. (C, D) Nissl staining revealed that PD intervention increased the mean optical density of Nissl bodies in the brain tissue. Furthermore, (E–G) PD intervention enhanced SOD activity (E) and reduced the levels of MDA (F) and ROS (G) in the ischemic side of the brain tissue. The magnification of HE staining and Nissl staining were 40× and 400×. *n* = 9 per group. Data are presented as the mean ± SD. *p < 0.05, **p < 0.01.

To further examine the effects of PD intervention on the pathological alterations in the brain tissue of CIRI rats, we conducted HE and Nissl staining. HE staining unveiled that CIRI rats exhibited pathological changes including increased glial cells, neuronal disarray, and reticular lesions in brain tissue compared to the Sham group. PD intervention effectively ameliorated these damages (Figure 2A,B). Nissl staining depicted a significant reduction in the number of Nissl bodies in CIRI rats compared to the Sham group, which was reversed with PD intervention (Figure 2C,D). Moreover, we evaluated the impact of PD on SOD activity and the levels of MDA and ROS in brain tissue. Results indicated that PD notably augmented SOD activity and attenuated the levels of MDA and ROS (Figure 2E – G). Differing doses of PD showcased dose‐dependent therapeutic effects on CIRI, prompting us to select the H‐PD group for further analysis.

### 3.2 PD Can Regulate the CXCL3/CXCR2 Axis and Related Inflammatory Pathways

**FIGURE 3:**
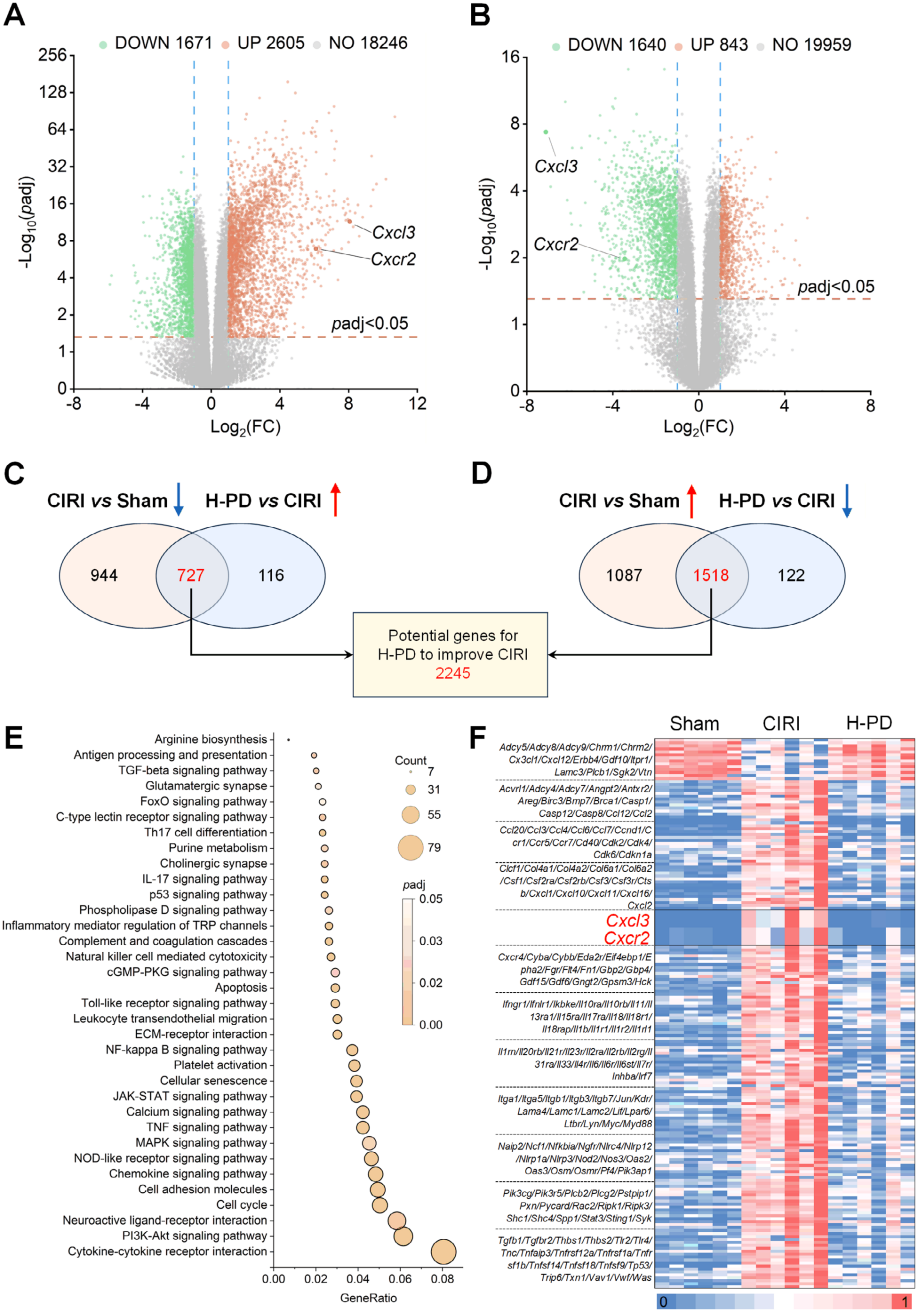
Impact of PD intervention on gene expression in the brain tissue of CIRI rats based on transcriptomics. (A–D) Differential genes were screened between the CIRI group vs. Sham group and the H‐PD group vs. CIRI group based on the criteria of |Log2(FoldChange)| ≥ 1 and padj ≤ 0.05. Volcano plots were used for visualization (A, B). The intersection genes were represented using Venn diagrams (C, D). (E, F) The results of the KEGG pathway enrichment analysis of these intersection genes showed that after PD intervention, Cytokine–cytokine receptor interaction and a large number of inflammatory pathways were enriched (E). The expression of genes related to these pathways was visualized using a heatmap, which revealed that the chemokine CXCL3 and its receptor CXCR2 were significantly downregulated (F). *n* = 6 per group.

Applying the criteria of |Log_2_(FoldChange)| ≥ 1 and padj ≤ 0.05, we identified differentially expressed genes between the CIRI group vs. Sham group and the H‐PD group vs. CIRI group (Figure 3A,B). Specifically, there were 1671 downregulated genes in the CIRI group vs. Sham group and 843 upregulated genes in the H‐PD group vs. CIRI group, with 727 genes shared between both sets. Conversely, there were 2605 upregulated genes in the CIRI group vs. Sham group and 1640 downregulated genes in the H‐PD group vs. CIRI group, with 1518 genes in common (Figure 3C,D). Detailed gene information is provided in the Supporting Information (Table S3). These shared genes represent potential targets of PD in CIRI. Thus, we proceeded to analyze the pathways associated with these genes using KEGG analysis. The outcomes revealed enrichment of numerous inflammatory pathways, including Cytokine–cytokine receptor interaction, PI3K‐Akt signaling pathway, Chemokine signaling pathway, NOD‐like receptor signaling pathway, and NF‐kappa B signaling pathway, among others. PD primarily modulates inflammatory‐related pathways to ameliorate CIRI (Figure 3E). Subsequently, we depicted these pathway‐related genes using a heatmap (Figure 3F, S2). Notably, CXCL3 exhibited the most prominent change among all differentially expressed genes post PD intervention (with a Log_2_(FoldChange) of −7.12 between the H‐PD group and CIRI group). Moreover, KEGG pathway enrichment analysis unveiled that CXCL3 is primarily linked with Cytokine–cytokine receptor interaction (KEGG ID: rno04060) and Chemokine signaling pathway (KEGG ID: rno04062). Upon further exploration of these pathways in the KEGG database, we discovered that CXCR2 serves as a critical receptor for CXCL3. Interestingly, PD significantly downregulated CXCR2 expression (with a Log_2_(FoldChange) of −3.45 between the H‐PD group and CIRI group) (Figure 3F). Based on these findings, we postulate that the CXCL3/CXCR2 axis may constitute a crucial target for PD in CIRI. Consequently, we conducted additional validation studies on the CXCL3/CXCR2 axis.

### 3.3 PD Suppressed the Expression of Factors Related to the CXCL3/CXCR2 Axis in the Brain Tissue of CIRI Rats

**FIGURE 4:**
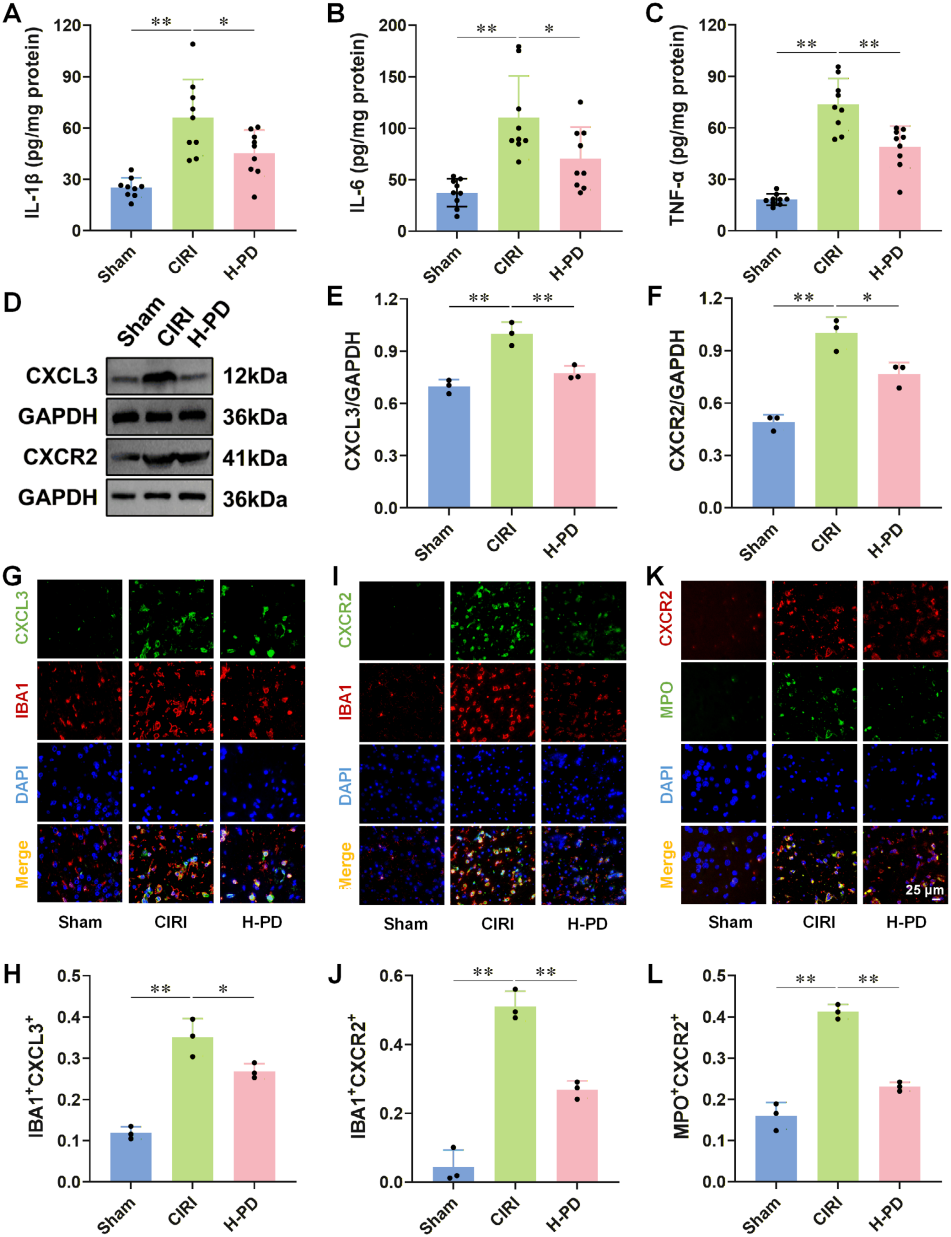
PD inhibits the expression of CXCL3/CXCR2 axis‐related factors in the brain tissue of CIRI rats. Using ELISA, we measured the effects of PD on the levels of proinflammatory cytokines IL‐1β, IL‐6, and TNF‐α in the brain tissue of CIRI rats. Western blot was used to detect the protein levels of CXCL3 and CXCR2 in the brain tissue, while immunofluorescence was used to assess the positive expression areas of IBA1^+^CXCL3^+^, IBA1^+^CXCR2^+^, and MPO^+^CXCR2^+^ in the brain tissue and thus evaluating the expression of CXCL3/CXCR2 axis‐related factors in CIRI rats. (A–C) PD intervention reduced the levels of proinflammatory cytokines IL‐1β (A), IL‐6 (B), and TNF‐α (C) in the ischemic side of CIRI rat brain tissue. (D–F) PD intervention downregulated the expression of CXCL3 (D, E) and CXCR2 (D, F). (G–L) PD intervention reduced the positive expression of IBA1^+^CXCL3^+^ (G, H), IBA1^+^CXCR2^+^ (I, J), and MPO^+^CXCR2^+^ (K, L). *n* = 9 for A–C, *n* = 3 for D–L. Data are presented as the mean ± SD. *p < 0.05, **p < 0.01.

Some studies have demonstrated that CXCL3 influences the activation of various inflammatory pathways by engaging the CXCR2 receptor [40]. Initially, we assessed the levels of inflammatory cytokines in the brain tissue. The findings revealed significantly elevated levels of proinflammatory cytokines (IL‐1β, IL‐6, and TNF‐α) in the brain tissue of CIRI rats compared to the Sham group, whereas PD intervention effectively mitigated these proinflammatory cytokine levels (Figure 4A – C). It is known that microglia can produce CXCL3, which binds to CXCR2 receptors on the surfaces of microglia and neutrophils, thereby fostering their migration and activation, thus contributing to the initiation of inflammatory responses [11, 40, 41, 42]. Subsequently, we investigated the impact of PD on the protein expressions of CXCL3 and CXCR2 (Figure 4D – F). Immunofluorescence staining was used to assess the expressions of CXCL3 and CXCR2 in microglia, and CXCR2 in neutrophils (Figure 4G – L, Table S4). The results revealed significant upregulation in the protein expressions of CXCL3 and CXCR2 in the brain tissue of CIRI rats, accompanied by noticeable increases in the positive expression areas of IBA1^+^CXCL3^+^, IBA1^+^CXCR2^+^, and MPO^+^CXCR2^+^. However, PD intervention reversed these outcomes.

### 3.4 PD Suppresses CXCL3 and CXCR2 Expression to Reduce Microglia Migration and Inflammatory Cytokine Release in in Vitro Experiments

Building upon the therapeutic efficacy of PD observed in CIRI rats and its ability to inhibit CXCL3/CXCR2 and inflammatory responses in vivo, we delved deeper into its therapeutic benefits and underlying mechanisms by establishing an in vitro model of OGD/R‐induced microglia activation.

**FIGURE 5:**
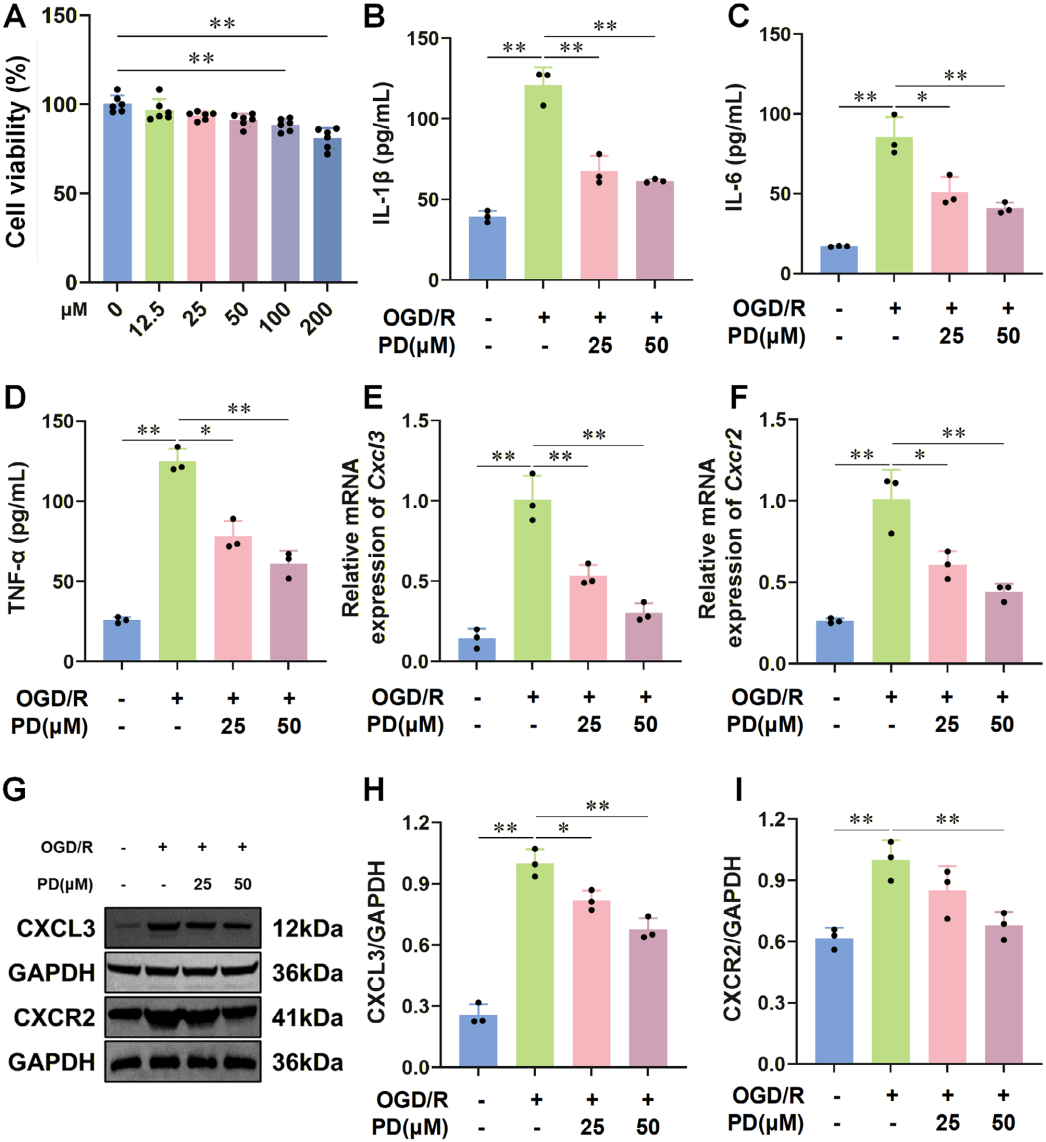
Effect of PD on the activation of BV2 cells induced by OGD/R. (A–D) MTT assay results showed that PD concentrations below 100 μM had no significant effect on the viability of BV2 cells (A). ELISA results indicated that PD intervention reduced the levels of proinflammatory cytokines IL‐1β (B), IL‐6 (C), and TNF‐α (D) in the supernatants of BV2 cells induced by OGD/R. (E–I) RT‐qPCR and Western blot results further demonstrated that PD intervention downregulated the gene and protein expression of CXCL3 (E, G, H) and CXCR2 (F, G, I). *n* = 6 for A, *n* = 3 for B–I. Data are presented as the mean ± SD. *p < 0.05, **p < 0.01.

MTT results indicated that PD concentrations below 100 μM had no significant impact on BV2 cell activity (Figure 5A). Consequently, we chose 25 and 50 μM concentrations of PD for further investigation. Using OGD/R, we intervened in BV2 cells to simulate CIRI in vitro while concurrently administering varying PD concentrations. ELISA outcomes revealed that following OGD/R intervention, the levels of inflammatory cytokines (IL‐1β, IL‐6, TNF‐α) in BV2 cell supernatants significantly increased, whereas PD intervention markedly attenuated these levels (Figure 5B–D).

**FIGURE 6:**
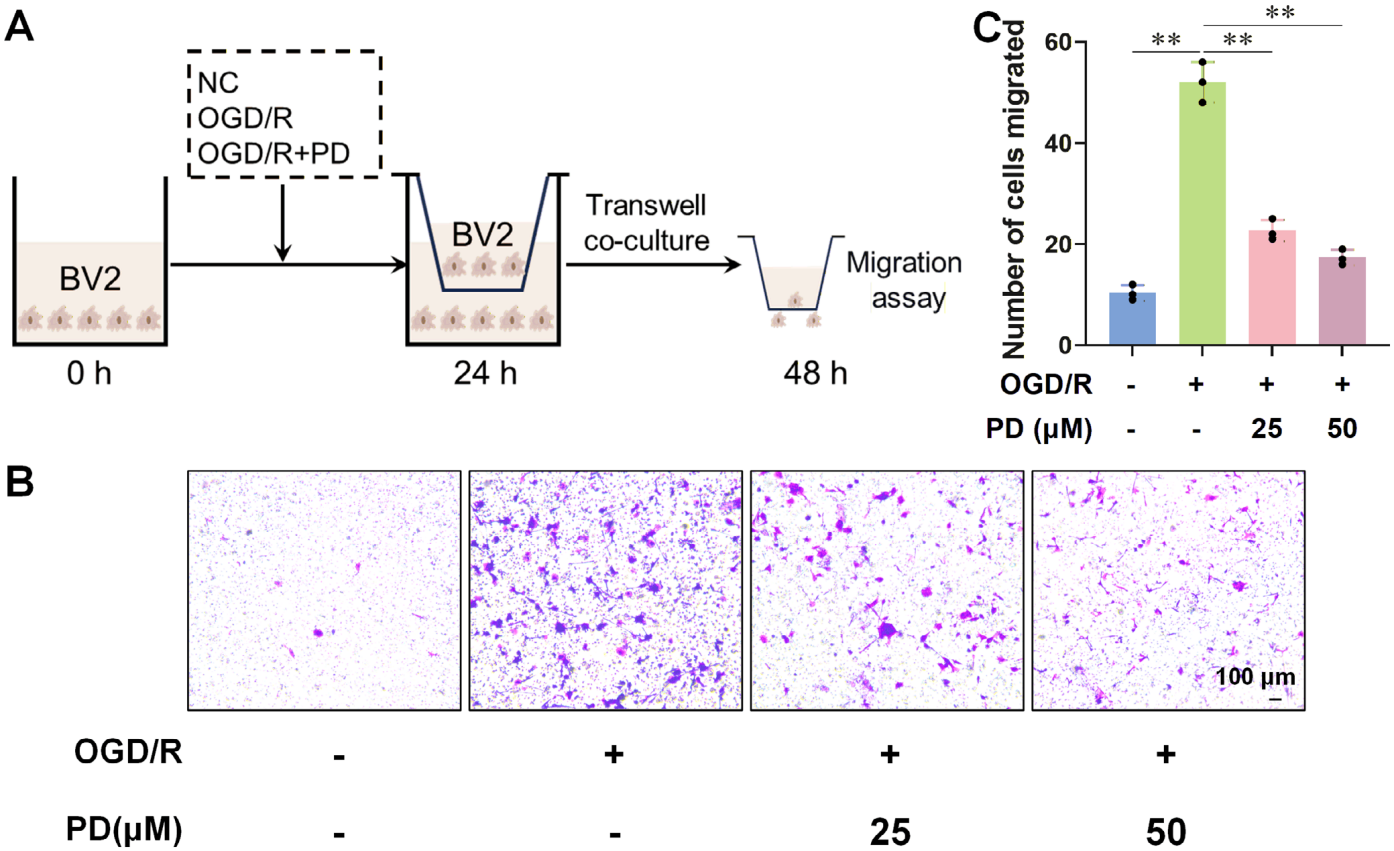
Effect of PD on the migration ability of OGD/R‐induced activated BV2 cells. (A) Schematic diagram of the Transwell cell migration experiment. We first seeded normal BV2 cells, OGD/R‐induced activated BV2 cells, and OGD/R‐induced activated BV2 cells that were treated with 50 μM PD in the lower chamber of a Transwell system. After 24 h incubation, normal BV2 cells were seeded in the upper chamber of the Transwell and further cultured for 24 h. (B, C) Crystal violet staining results showed that PD intervention reduced the number of migrating BV2 cells. *n* = 3 per group. Data are presented as the mean ± SD. *p < 0.05, **p < 0.01.

RT‐qPCR and Western blot analyses demonstrated that PD intervention significantly reduced the gene and protein expressions of CXCL3 and CXCR2 in the OGD/R cell model (Figure 5E–I). Subsequently, we assessed PD's effect on microglial migration via Transwell experiments. We seeded normal BV2 cells, OGD/R‐induced activated cells, and PD‐treated OGD/R‐induced activated cells in the lower chamber of Transwell plates, while normal BV2 cells were seeded in the upper chamber (Figure 6A). Results indicated that post‐OGD/R intervention, the number of migrating BV2 cells significantly increased, which PD intervention effectively reversed (Figure 6B,C). Among the different PD doses tested, 50 μM PD demonstrated the most potent intervention effect, prompting its selection for subsequent validation experiments.

**FIGURE 7:**
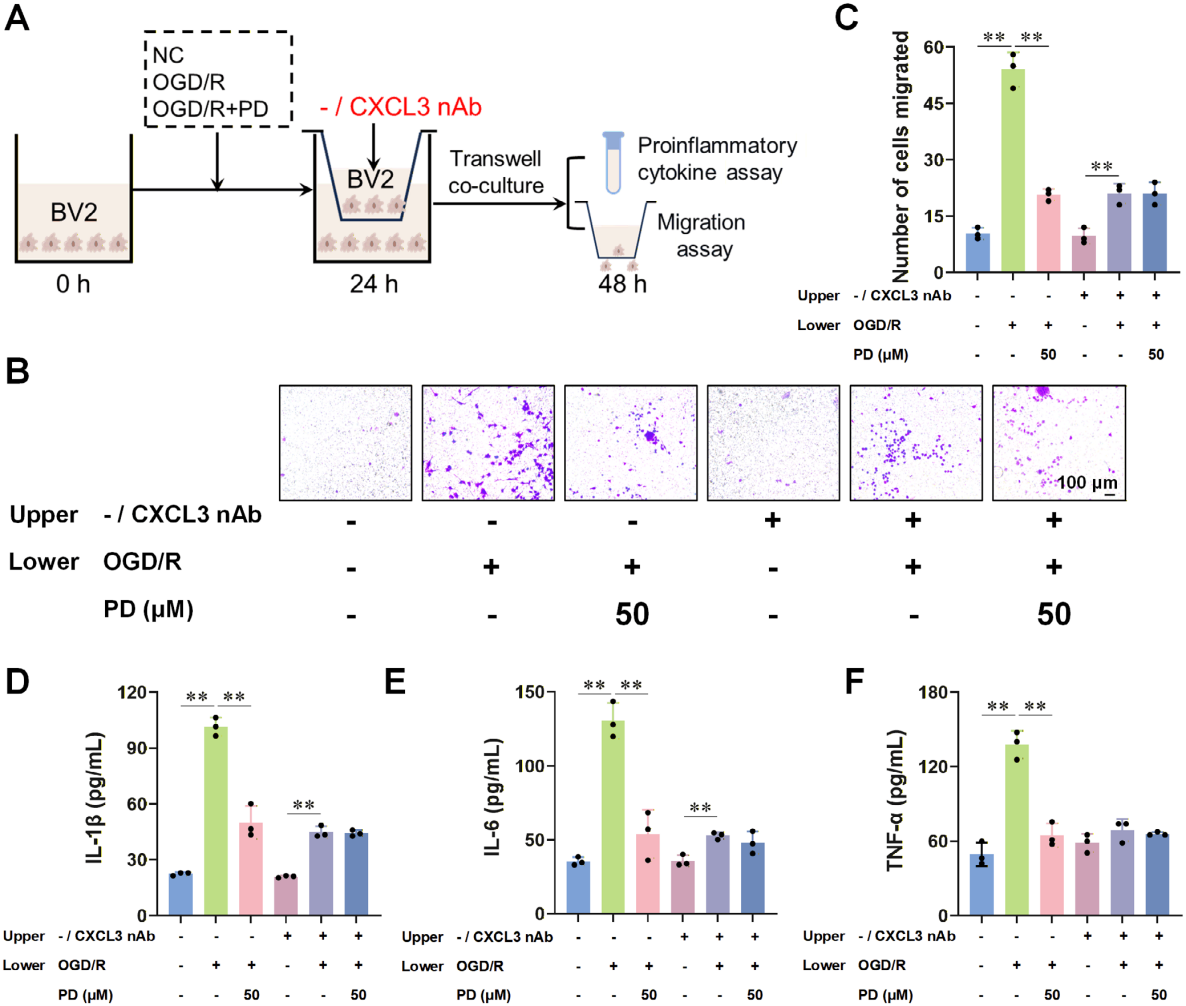
Anti‐CXCL3 neutralizing antibody abolishes the interventional effect of PD on OGD/R‐induced activation of BV2 cells. (A) Schematic diagram of the Transwell cell migration experiment. We first seeded normal BV2 cells, OGD/R‐induced activated BV2 cells, and 50 μM PD‐treated OGD/R‐induced activated BV2 cells in the lower chamber of a Transwell system and incubated them for 24 h. Subsequently, we seeded normal BV2 cells and BV2 cells treated with anti‐CXCL3 neutralizing antibody in the upper chamber of the Transwell system and continued culturing for another 24 h. (B–F) Anti‐CXCL3 neutralizing antibody abolished the suppressive effect of PD on the migration number of BV2 cells (B, C) and the levels of proinflammatory cytokines IL‐1β (D), IL‐6 (E), and TNF‐α (F) in the supernatants of OGD/R‐induced activated BV2 cells. *n* = 3 per group. Data are presented as the mean ± SD. *p < 0.05, **p < 0.01.

To evaluate the impact of PD on the CXCL3/CXCR2 axis, we placed normal, OGD/R‐induced activated, and PD‐treated OGD/R‐induced activated BV2 cells in the lower chamber of Transwell plates. Concurrently, we introduced normal BV2 cells and BV2 cells treated with CXCL3 neutralizing antibody into the upper chamber (Figure 7A). Our analysis revealed that, compared to conditions without CXCL3 neutralizing antibody treatment, its inclusion reduced both the migration of OGD/R‐induced activated BV2 cells (Figure 7B,C) and the levels of proinflammatory cytokines in the supernatant (Figure 7D – F). However, CXCL3 neutralizing antibody treatment did not yield a significant difference in these parameters, irrespective of PD treatment.

**FIGURE 8:**
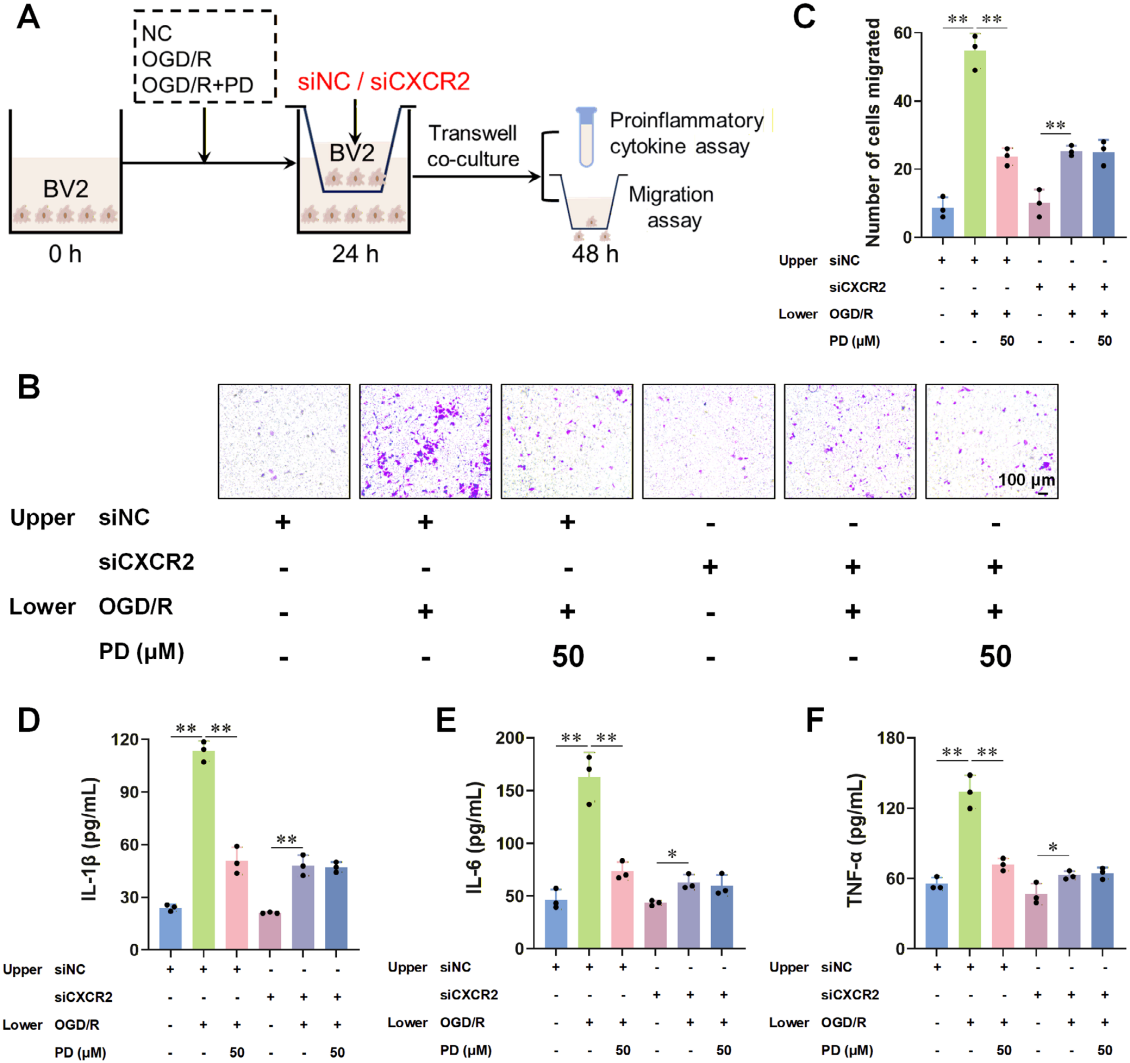
SiCXCR2 abolishes the interventional effect of PD on OGD/R‐induced activation of BV2 cells. (A) Schematic diagram of the Transwell cell migration experiment. Initially, we seeded normal BV2 cells, OGD/R‐induced activated BV2 cells, and OGD/R‐induced activated BV2 cells that were treated with 50 μM PD in the lower chamber of a Transwell system. After 24 h incubation, we seeded BV2 cells transfected with SiNC and SiCXCR2, respectively, in the upper chamber of the Transwell system and continued culturing for another 24 h. (B–F) SiCXCR2 abolished the suppressive effect of PD on the migration number of BV2 cells (B, C) and the levels of proinflammatory cytokines IL‐1β (D), IL‐6 (E), and TNF‐α (F) in the supernatants of OGD/R‐induced activated BV2 cells. *n* = 3 per group. Data are presented as the mean ± SD. *p < 0.05, **p < 0.01.

Subsequently, we seeded normal, OGD/R‐induced activated, and PD‐treated OGD/R‐induced activated BV2 cells in the lower chamber of Transwell plates. In the upper chamber, BV2 cells were transfected with either SiNC or SiCXCR2 (Figure 8A). Our observations indicated that compared to SiNC transfection, SiCXCR2 transfection reduced the migration of OGD/R‐induced activated BV2 cells or the levels of proinflammatory cytokines in the supernatant. However, following CXCR2 silencing, there were no significant differences in these parameters, regardless of PD treatment (Figure 8B–F).

Considering that PD reduced the level of MPO, a neutrophil marker, in the brain tissue of CIRI, we conducted in vitro experiments to preliminarily explore the effect of PD on neutrophil migration. MTT assays revealed that PD concentrations below 50 μM did not affect neutrophil viability (Figure S3A). Subsequently, we induced neutrophil migration using CXCL3 and intervened with 50 μM PD. The results showed that CXCL3 treatment significantly increased neutrophil migration, whereas PD intervention reduced the number of migrating neutrophils (Figure S3B,C).

## 4 Discussion

CIRI poses a significant challenge in the prognosis of ischemic stroke [43]. While some studies have suggested that PD holds promise in protecting the central nervous system [17], its underlying mechanism on CIRI remains unclear. Our objective is to elucidate the mechanism by which PD acts on CIRI, focusing on the CXCL3/CXCR2 axis, utilizing transcriptomics technology alongside in vivo and in vitro experiments. Bederson's scale score [30, 31], postural reflex test [32], and asymmetry score [33] serve as key indicators for evaluating the pathological condition of CIRI. Our observations in CIRI rats revealed an augmentation in infarct area, neuronal disarray, reduced Nissl body count, diminished SOD activity, and heightened levels of MDA and ROS, consistent with the aforementioned pathological state. As a commonly used adjuvant medication for treating CIRI, ginaton has been demonstrated to effectively restore neurological function, and we used ginaton as a positive control [28]. However, the side effects of ginaton have been reported in various aspects, such as causing gastrointestinal distress, headache, and allergy [44]. Our study demonstrated that PD intervention effectively mitigates the pathological manifestations, facilitates the restoration of neurological and motor functions, and attenuates these pathological changes. This suggests that PD may serve as an alternative therapy for CIRI. Currently, oral administration is the predominant method for delivering PD, which offers advantages such as high patient compliance and convenient dosing. However, there remains significant potential for enhancing its efficacy. From a safety perspective, PD exhibits low toxicity. In mouse models, no deaths or neurobehavioral abnormalities were observed following intraperitoneal injection of 100 mg/kg PD [45]. In vitro experiments have demonstrated that the safety threshold of PD for most cells can reach up to 100 μg/mL [46, 47]. Notably, the existing therapeutic window data available to guide clinical practice primarily originate from studies on the combined use of PD with other drugs, such as palmitoylethanolamide [48]. In contrast, the safe dosage range for single‐component PD has yet to be explored. Therefore, conducting clinical trials focused on single‐component PD to further clarify its safe dosage range and pharmacokinetics is crucial for achieving its genuine clinical translation.

Moreover, we utilized transcriptomics technology to delve deeper into the mechanism by which PD operates on CIRI. Our transcriptomics analysis unveiled that PD modulates numerous inflammation‐related pathways, with notable regulation observed in CXCL3 and CXCR2. Consequently, we proceeded to validate the impact of PD on the CXCL3/CXCR2 axis and the ensuing inflammatory response. CXCL3, a low‐molecular‐weight single‐chain protein classified within the CXC chemokine family, plays a pivotal role in CIRI [49]. Within CIRI, CXCL3 can be synthesized by microglia and function to recruit and activate CXCR2‐expressing cells, such as microglia and macrophages, via autocrine or paracrine pathways [11]. This activation initiates diverse inflammatory cascades, including the PI3K‐Akt signaling pathway and the NOD‐like receptor signaling pathway [40]. Notably, studies have highlighted a significant elevation in CXCL3 expression levels in models of meningitis. Elevated CXCL3 levels can induce the proinflammatory polarization of CXCR2‐expressing microglia, subsequently amplifying the production of proinflammatory cytokines like IL‐6 and exacerbating the inflammatory response [42]. Targeting the CXCL3/CXCR2 axis has shown efficacy in reducing the inflammatory response [41]. Our findings demonstrate that PD profoundly suppresses the expression of the CXCL3/CXCR2 axis, thus mitigating the inflammatory response. This suggests that PD may ameliorate CIRI by inhibiting the CXCL3/CXCR2 axis. Additionally, we observed that PD treatment down‐regulated most of the genes in the NF‐kappa B signaling pathway. Extensive research has reported that NF‐κB regulates the expression of the cytokine CXCL3 [50, 51, 52]. NF‐κB translocates into the nucleus via phosphorylation, promoting the transcription of downstream CXCL3 and thereby exacerbating disease progression [53]. Conversely, inhibition of NF‐κB function significantly downregulates CXCL3 expression [51]. Taken together, the inhibitory effect of PD on CXCL3 may be related to the suppression of the NF‐κB pathway. However, the precise regulatory mechanism and target sites still require further validation. Further transcriptomic analysis, such as GSEA, is still warranted to observe the overall expression trends of pathway‐level genes in CIRI and to cross‐validate with the current KEGG enrichment analysis results, thereby enhancing the robustness of our conclusions.

In our study, we only tested the effects of PD on the CXCL3/CXCR2 axis in microglia by detecting the positive area of IBA1^+^CXCL3^+^and IBA1^+^CXCR2^+^ in brain. The results showed that PD treatment reduced the double positive area of IBA1^+^CXCL3^+^and IBA1^+^CXCR2^+^ in brain, indicating the inhibitory effects of PD on the CXCL3/CXCR2 axis in vivo. These data could also reveal the inhibitory effects of PD on microglia activation, as activated microglia could secrete several chemokines such as CXCL3. Future study can be conducted to further detect the direct effect of PD on microglia by testing the markers related to microglia activation and polarization such as CD68, CD86, and iNOS. Notably, CXCL3 fluorescence co‐localizes with the labeled portions of microglia, suggesting that microglia may be one of the sources of CXCL3. Multiple independent studies have collectively confirmed that CXCL3 can be produced by neurons, astrocytes, and microglia in brain tissue [11, 54]. Single cell sequencing will be conducted in the future to confirm this result.

We proceeded to further validate the impact of PD on the CXCL3/CXCR2 axis in microglia by establishing an in vitro OGD/R model. OGD/R serves as a well‐established cell model for simulating CIRI in vitro [39]. Our investigation revealed that PD intervention led to a decrease in the secretion of proinflammatory cytokines by BV2 cells and a reduction in the expression of CXCL3 and CXCR2 in BV2 cells following OGD/R induction. Subsequently, we used a Transwell cell migration assay to evaluate the influence of PD on the migratory capacity of BV2 cells. The Transwell assay serves as a crucial tool for studying cellular behavior in vitro, allowing for the assessment of cell migration, chemotaxis, and invasiveness in response to various stimuli, such as chemokines. Quantitative indicators, including the number of cells migrating or invading through the chamber bottom or culture medium, as well as alterations in cell movement or chemotactic response parameters, are commonly utilized in these assays [55]. Our findings indicated that PD intervention impeded the migration of BV2 cells. Having established the therapeutic efficacy of PD, we proceeded to investigate the impact of PD on the CXCL3/CXCR2 axis using CXCL3 neutralizing antibodies and SiCXCR2 to interfere with BV2 cells. Previous studies have demonstrated that CXCL3 neutralizing antibodies can alleviate neuropathic pain [11], while SiCXCR2 can inhibit the activation of multiple inflammatory pathways and reduce the inflammatory response in postoperative abdominal adhesions [56]. Our study revealed that intervention with CXCL3 neutralizing antibodies and SiCXCR2 nullified the effects of PD on the secretion of proinflammatory cytokines and the migratory ability of BV2 cells, indicating that PD effectively ameliorates CIRI by inhibiting the CXCL3/CXCR2 axis. Remarkably, the BV2 cells selected for in vitro experiments possess advantages such as ease of handling, reproducibility, and the ability to exhibit fundamental characteristics of microglia [57]. However, BV2 cells cannot fully replicate all the functional details of primary microglia [58], and there are several limitations associated with their use, including lower reactivity to oxygen–glucose deprivation/reperfusion (OGD/R) compared to primary microglia [59], a tendency to undergo dedifferentiation or phenotypic changes resembling microglial alterations [60], and a lack of interaction with other brain cell types. Given these functional disparities, caution should be exercised when extrapolating findings from BV2 cells to microglia in the brain under inflammatory conditions. To extend the pathophysiological significance of our research conclusions, the relevant results should be further validated in primary microglia.

CIRI involves interactions among multiple cell types, including neurons, brain microvascular endothelial cells (BMECs), astrocytes, neutrophils, and microglia [61]. Among them, neutrophils have been reported to infiltrate the damaged site by binding to the chemokine CXCL3 during the early stage of CIRI, exacerbating CIRI injury [62]. A recent study has revealed that neutrophils can induce functional changes in microglia, leading to the inhibition of microglial phagocytosis, and thereby exacerbate CIRI [63]. We also observed high expression of the neutrophil marker in the cerebral cortex of CIRI. Blocking the CXCR2 receptor on neutrophils can reduce the occurrence of CIRI [64]. In vitro experiments have revealed that PD decreased the number of CXCL3‐induced neutrophil migration, suggesting that PD may reduce neutrophil migration by blocking the CXCL3/CXCR2 axis. Concurrently, recent studies have revealed that microglia—the core effector cells of innate immunity in the central nervous system—not only secrete CXCL3 and other chemokines to mediate neutrophil recruitment [11, 12] but also express CXCR2 receptors themselves, participating in inflammatory signal amplification loops [13]. In the future, a neutrophil‐microglia co‐culture system and a neutrophil‐specific CXCR2 knockout rat model could be used to further verify whether PD inhibits neutrophil migration through the CXCL3/CXCR2 pathway.

## 5 Conclusion

**FIGURE 9:**
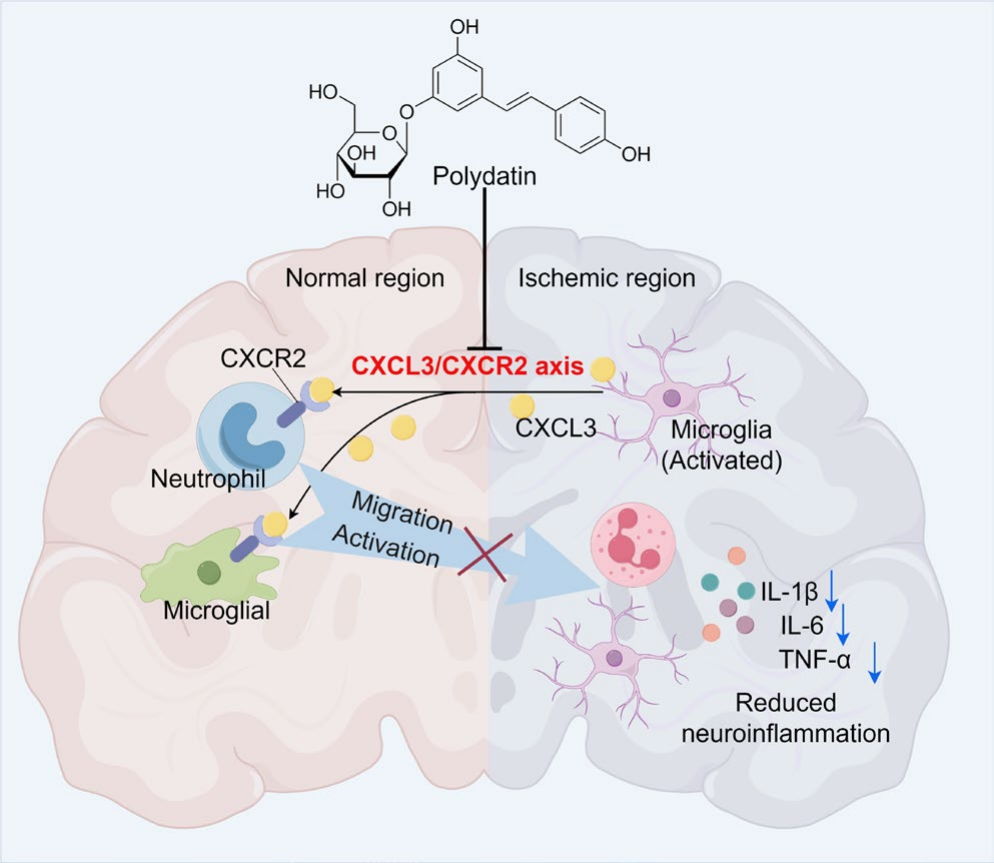
PD can act on the CXCL3/CXCR2 axis, thereby inhibiting migration and activation of microglia and neutrophil, and thus alleviating the inflammatory response associated with CIRI (by Figdraw).

This study demonstrates that PD effectively alleviates CIRI by modulating the CXCL3/CXCR2 axis, thereby suppressing microglia/macrophage migration and activation and attenuating neuroinflammation (Figure 9). PD holds promise as a novel and potent therapeutic agent for CIRI treatment, and the pivotal role played by the CXCL3/CXCR2 axis in this context will provide valuable insights for the development of other natural products. However, some limitations warrant attention. The upstream regulatory mechanisms of PD on the CXCL3/CXCR2 axis remain to be further elucidated. Meanwhile, the synergistic regulatory effects of PD on key cell populations involved in CIRI, such as microglia and neutrophils, necessitate clarification through the integration of single‐cell sequencing and in vitro and in vivo target validation. Furthermore, the safe dosage range and pharmacokinetics of PD are still unknown, which are of critical importance for its clinical translation.

## Author Contributions

Youxiang Cui: Writing – original draft, Investigation, Funding acquisition; Fangmei Hu: Writing – original draft, Investigation, Validation, Data curation; Shixin Wang: Investigation, Validation, Data curation; Zehua Liu, Jinzhou Chen: Validation, Data curation, Formal analysis; Mingyue Cui, Leilei Wang: Investigation, Validation Formal analysis; Yuming Wang, Ning Wang:Validation, Data curation；Yuhong Bian, Shuquan Lv: Writing – original draft, Investigation, Data curation, Validation, Conceptualization; Huantian Cui: Writing – review and editing, Conceptualization.

## Funding

This work was supported by Hebei Natural Science Foundation (H2024110042) and the National Natural Science Foundation of China (U21A20400).

## Conflicts of Interest

The authors declare no conflicts of interest.

## Supporting information


**Figure S1:** Constitutional formula of Polydatin.
**Figure S2:** Heatmap of gene expression related to the NF‐kappa B signaling pathway.
**Figure S3:** PD reduces the number of CXCL3‐induced neutrophil migration.
(A) MTT assay results showed that PD concentrations below 50 μM had no significant effect on the viability of neutrophils. (B, C) Crystal violet staining results showed that PD intervention reduced the number of migrating neutrophils. *n* = 6 for A, *n* = 3 for B, C. Data are presented as the mean ± SD. **p < 0.01, *p < 0.05.
**Table S1:** Neurological function evaluation.
**Table S2:** Primer sequence.
**Table S3:** Common genes.
**Table S4:** Statistical analysis of fluorescence intensity in rat brain tissues among groups in Figure 4G.

## Data Availability

The data that support the findings of this study are available from the corresponding author upon reasonable request.

## References

[1] A. Ajoolabady , S. Wang , G. Kroemer , et al., “Targeting Autophagy in Ischemic Stroke: From Molecular Mechanisms to Clinical Therapeutics,” Pharmacology & Therapeutics 225 (2021): 107848. 33823204 10.1016/j.pharmthera.2021.107848 PMC8263472

[2] L. Shen , Q. Gan , Y. Yang , et al., “Mitophagy in Cerebral Ischemia and Ischemia/Reperfusion Injury,” Frontiers in Aging Neuroscience 13 (2021): 687246. 34168551 10.3389/fnagi.2021.687246 PMC8217453

[3] L. Huang , X. Li , Y. Liu , et al., “Curcumin Alleviates Cerebral Ischemia‐Reperfusion Injury by Inhibiting NLRP1‐Dependent Neuronal Pyroptosis,” Current Neurovascular Research 18 (2021): 189–196. 34109908 10.2174/1567202618666210607150140

[4] Q. Cai , C. Zhao , Y. Xu , et al., “Qingda Granule Alleviates Cerebral Ischemia/Reperfusion Injury by inhibitingTLR4/NF‐kappaB/NLRP3 Signaling in Microglia,” Journal of Ethnopharmacology 324 (2024): 117712. 38184025 10.1016/j.jep.2024.117712

[5] L. Luo , M. Liu , Y. Fan , et al., “Intermittent Theta‐Burst Stimulation Improves Motor Function by Inhibiting Neuronal Pyroptosis and Regulating Microglial Polarization via TLR4/NFkappaB/NLRP3 Signaling Pathway in Cerebral Ischemic Mice,” Journal of Neuroinflammation 19 (2022): 141. 35690810 10.1186/s12974-022-02501-2 PMC9188077

[6] H. Shen , H. Pei , L. Zhai , Q. Guan , and G. Wang , “Salvianolic Acid C Improves Cerebral Ischemia Reperfusion Injury Through Suppressing Microglial Cell M1 Polarization and Promoting Cerebral Angiogenesis,” International Immunopharmacology 110 (2022): 109021. 35810493 10.1016/j.intimp.2022.109021

[7] C. J. Smith , S. Hulme , A. Vail , et al., “SCIL‐STROKE (Subcutaneous Interleukin‐1 Receptor Antagonist in Ischemic Stroke): A Randomized Controlled Phase 2 Trial,” Stroke 49 (2018): 1210–1216. 29567761 10.1161/STROKEAHA.118.020750

[8] M. Liu , Z. Xu , L. Wang , et al., “Cottonseed Oil Alleviates Ischemic Stroke Injury by Inhibiting the Inflammatory Activation of Microglia and Astrocyte,” Journal of Neuroinflammation 17 (2020): 270. 32917229 10.1186/s12974-020-01946-7 PMC7488511

[9] Y. Wang , J. Luo , and S. Y. Li , “Nano‐Curcumin Simultaneously Protects the Blood‐Brain Barrier and Reduces M1 Microglial Activation During Cerebral Ischemia‐Reperfusion Injury,” ACS Applied Materials & Interfaces 11 (2019): 3763–3770. 30618231 10.1021/acsami.8b20594

[10] X. Zhao , J. Zhu , S. Chen , et al., “Neural Stem Cell‐Derived Exosomes Improve Neurological Function in Rats With Cerebral Ischemia‐Reperfusion Injury by Regulating Microglia‐Mediated Inflammatory Response,” Journal of Inflammation Research 16 (2023): 3079–3092. 37520663 10.2147/JIR.S414121 PMC10378531

[11] A. Piotrowska , E. Rojewska , K. Pawlik , et al., “Pharmacological Blockade of Spinal CXCL3/CXCR2 Signaling by NVP CXCR2 20, a Selective CXCR2 Antagonist, Reduces Neuropathic Pain Following Peripheral Nerve Injury,” Frontiers in Immunology 10 (2019): 2198. 31616413 10.3389/fimmu.2019.02198 PMC6775284

[12] I. Rana , V. Suphapimol , J. R. Jerome , D. M. Talia , D. Deliyanti , and J. L. Wilkinson‐Berka , “Angiotensin II and Aldosterone Activate Retinal Microglia,” Experimental Eye Research 191 (2020): 107902. 31884019 10.1016/j.exer.2019.107902

[13] M. Serdar , K. Kempe , R. Herrmann , et al., “Involvement of CXCL1/CXCR2 During Microglia Activation Following Inflammation‐Sensitized Hypoxic‐Ischemic Brain Injury in Neonatal Rats,” Frontiers in Neurology 11 (2020): 540878. 33123073 10.3389/fneur.2020.540878 PMC7573390

[14] H. D. Chen , M. Z. Jiang , Y. Y. Zhao , et al., “Effects of Breviscapine on Cerebral Ischemia‐Reperfusion Injury and Intestinal Flora Imbalance by Regulating the TLR4/MyD88/NF‐kappaB Signaling Pathway in Rats,” Journal of Ethnopharmacology 300 (2023): 115691. 36087844 10.1016/j.jep.2022.115691

[15] P. Ahmad , S. S. Alvi , D. Iqbal , and M. S. Khan , “Insights Into Pharmacological Mechanisms of Polydatin in Targeting Risk Factors‐Mediated Atherosclerosis,” Life Sciences 254 (2020): 117756. 32389832 10.1016/j.lfs.2020.117756

[16] Z. Sun , Y. Wang , X. Pang , X. Wang , and H. Zeng , “Mechanisms of Polydatin Against Spinal Cord Ischemia‐Reperfusion Injury Based on Network Pharmacology, Molecular Docking and Molecular Dynamics Simulation,” Bioorganic Chemistry 140 (2023): 106840. 37683540 10.1016/j.bioorg.2023.106840

[17] L. E. Schimith , S. M. Dos , B. D. Arbo , et al., “Polydatin as a Therapeutic Alternative for Central Nervous System Disorders: A Systematic Review of Animal Studies,” Phytotherapy Research 36 (2022): 2852–2877. 35614539 10.1002/ptr.7497

[18] N. Qian , C. Lu , T. Wei , et al., “Epidemiological Trends and Forecasts in Stroke at Global, Regional and National Levels,” Journal of Stroke and Cerebrovascular Diseases 34 (2025): 108347. 40381865 10.1016/j.jstrokecerebrovasdis.2025.108347

[19] D. Sun , X. Guo , L. Ling , et al., “Sex‐Related Differences in Endovascular Treatment Outcomes for Acute Large Infarcts: The ANGEL‐ASPECT Subanalysis,” Stroke 56 (2025): 2033–2042. 40340582 10.1161/STROKEAHA.124.050025

[20] G. Bereda , “Demographic Disparities in Stroke Occurrence: Insights From an Integrative Review of Emerging Trends,” Brain and Behavior: A Cognitive Neuroscience Perspective 15 (2025): e70575. 10.1002/brb3.70575 PMC12321967 40760721

[21] L. M. Achzet and D. A. Jackson , “Sex‐Dependent Differences in the Ischemia/Reperfusion‐Induced Expression of AMPA Receptors,” International Journal of Molecular Sciences 25 (2024): 2231. 38396906 10.3390/ijms25042231 PMC10889403

[22] Y. El‐Hakim , K. K. Mani , A. Eldouh , et al., “Sex Differences in Stroke Outcome Correspond to Rapid and Severe Changes in Gut Permeability in Adult Sprague‐Dawley Rats,” Biology of Sex Differences 12 (2021): 14. 33451354 10.1186/s13293-020-00352-1 PMC7811247

[23] K. Kimura , Y. H. Liu , and C. L. Hsieh , “Amygdalin's Neuroprotective Effects on Acute Ischemic Stroke in Rats,” Journal of Ethnopharmacology 345 (2025): 119621. 40081511 10.1016/j.jep.2025.119621

[24] E. D. Abd , M. A. Rabie , R. A. Mohamed , et al., “Acute Calcitriol Treatment Alleviates Cerebral Ischemia Reperfusion Injury via Activation of MasR/PI3K/Akt/CREB Axis and Suppression of AT‐1R/NF‐kappaB p65 Signaling Cascade,” European Journal of Pharmacology 1007 (2025): 178240. 41101680 10.1016/j.ejphar.2025.178240

[25] W. Li , Y. Fan , and S. A. Hussain , “Neuroprotective Effects of Sinomenine and Metformin in Diabetic Stroke: Role of NLRP3/Caspase‐1 and Mitophagy,” Experimental Physiology 111, no. 2 (2026): 489–500. 41110997 10.1113/EP093135 PMC12857461

[26] X. Yin , J. Wang , S. Yang , et al., “Sam50 Exerts Neuroprotection by Maintaining the Mitochondrial Structure During Experimental Cerebral Ischemia/Reperfusion Injury in Rats,” CNS Neuroscience & Therapeutics 28 (2022): 2230–2244. 36074556 10.1111/cns.13967 PMC9627377

[27] Y. Chen , H. Li , Y. Yang , et al., “Polygalasaponin F Ameliorates Middle Cerebral Artery Occlusion‐Induced Focal Ischemia / Reperfusion Injury in Rats Through Inhibiting TXNIP/NLRP3 Signaling Pathway,” Journal of Neuroimmunology 387 (2024): 578281. 38198981 10.1016/j.jneuroim.2023.578281

[28] J. Shang , Q. Li , T. Jiang , et al., “Systems Pharmacology, Proteomics and in Vivo Studies Identification of Mechanisms of Cerebral Ischemia Injury Amelioration by Huanglian Jiedu Decoction,” Journal of Ethnopharmacology 293 (2022): 115244. 35378193 10.1016/j.jep.2022.115244

[29] X. Zhang , Z. Wang , X. Li , et al., “Polydatin Protects Against Atherosclerosis by Activating Autophagy and Inhibiting Pyroptosis Mediated by the NLRP3 Inflammasome,” Journal of Ethnopharmacology 309 (2023): 116304. 36870461 10.1016/j.jep.2023.116304

[30] T. A. Asgari , L. Dargahi , S. Nasoohi , et al., “The Conditioned Medium of Human Embryonic Stem Cell‐Derived Mesenchymal Stem Cells Alleviates Neurological Deficits and Improves Synaptic Recovery in Experimental Stroke,” Journal of Cellular Physiology 236 (2021): 1967–1979. 32730642 10.1002/jcp.29981

[31] J. B. Bederson , L. H. Pitts , M. Tsuji , M. C. Nishimura , R. L. Davis , and H. Bartkowski , “Rat Middle Cerebral Artery Occlusion: Evaluation of the Model and Development of a Neurologic Examination,” Stroke 17 (1986): 472–476. 3715945 10.1161/01.str.17.3.472

[32] G. Chen , X. Ye , J. Zhang , et al., “Limb Remote Ischemic Postconditioning Reduces Ischemia‐Reperfusion Injury by Inhibiting NADPH Oxidase Activation and MyD88‐TRAF6‐P38MAP‐Kinase Pathway of Neutrophils,” International Journal of Molecular Sciences 17 (2016): 1971. 27898007 10.3390/ijms17121971 PMC5187771

[33] B. Zhao , J. Zhu , Y. Fei , et al., “JLX001 Attenuates Blood‐Brain Barrier Dysfunction in MCAO/R Rats via Activating the Wnt/Beta‐Catenin Signaling Pathway,” Life Sciences 260 (2020): 118221. 32768578 10.1016/j.lfs.2020.118221

[34] Y. Liu , X. Xue , H. Zhang , et al., “Neuronal‐Targeted TFEB Rescues Dysfunction of the Autophagy‐Lysosomal Pathway and Alleviates Ischemic Injury in Permanent Cerebral Ischemia,” Autophagy 15 (2019): 493–509. 30304977 10.1080/15548627.2018.1531196 PMC6351122

[35] K. Chen , N. Li , F. Fan , et al., “Tibetan Medicine Duoxuekang Capsule Ameliorates High‐Altitude Polycythemia Accompanied by Brain Injury,” Frontiers in Pharmacology 12 (2021): 680636. 34045970 10.3389/fphar.2021.680636 PMC8144525

[36] X. Wang , Y. Hou , Q. Li , et al., “Rhodiola Crenulata Attenuates Apoptosis and Mitochondrial Energy Metabolism Disorder in Rats With Hypobaric Hypoxia‐Induced Brain Injury by Regulating the HIF‐1alpha/microRNA 210/ISCU1/2(COX10) Signaling Pathway,” Journal of Ethnopharmacology 241 (2019): 111801. 30878546 10.1016/j.jep.2019.03.028

[37] Q. Xie , R. Ma , X. Guo , H. Chen , and J. Wang , “Benzoinum From *Styrax tonkinensis* (Pierre) Craib ex Hart Exerts a NVU Protective Effect by Inhibiting Cell Apoptosis in Cerebral Ischaemia Rats,” Journal of Ethnopharmacology 265 (2021): 113355. 32891816 10.1016/j.jep.2020.113355

[38] H. Cui , Y. Jin , N. Wang , et al., “Mechanic Evaluation of Wu‐Mei‐Pill on Colitis‐Associated Colorectal Cancer: An Integrated Transcriptomics, Metabolomics, and Experimental Validation Study,” Phytomedicine 128 (2024): 155509. 38452403 10.1016/j.phymed.2024.155509

[39] X. Xu , W. Gao , L. Li , et al., “Annexin A1 Protects Against Cerebral Ischemia‐Reperfusion Injury by Modulating Microglia/Macrophage Polarization via FPR2/ALX‐Dependent AMPK‐mTOR Pathway,” Journal of Neuroinflammation 18 (2021): 119. 34022892 10.1186/s12974-021-02174-3 PMC8140477

[40] J. Korbecki , K. Kojder , P. Kapczuk , et al., “The Effect of Hypoxia on the Expression of CXC Chemokines and CXC Chemokine Receptors‐A Review of Literature,” International Journal of Molecular Sciences 22 (2021): 843. 33467722 10.3390/ijms22020843 PMC7830156

[41] H. Li , M. Zhang , Q. Zhao , et al., “Self‐Recruited Neutrophils Trigger Over‐Activated Innate Immune Response and Phenotypic Change of Cardiomyocytes in Fulminant Viral Myocarditis,” Cell Discovery 9 (2023): 103. 37816761 10.1038/s41421-023-00593-5 PMC10564723

[42] X. Qu , B. Dou , R. Yang , C. Tan , H. Chen , and X. Wang , “C‐X‐C Motif Chemokine 3 Promotes the Inflammatory Response of Microglia After *Escherichia coli* ‐Induced Meningitis,” International Journal of Molecular Sciences 24 (2023): 10432. 37445610 10.3390/ijms241310432 PMC10341832

[43] W. Yu , N. Yin , Y. Yang , et al., “Rescuing Ischemic Stroke by Biomimetic Nanovesicles Through Accelerated Thrombolysis and Sequential Ischemia‐Reperfusion Protection,” Acta Biomaterialia 140 (2022): 625–640. 34902617 10.1016/j.actbio.2021.12.009

[44] X. Dang , R. Wang , and Y. Liu , “Disulfiram‐Like Reaction With Ginaton: A Case Report and Literature Review,” Clinical Therapeutics 45 (2023): 1151–1154. 37722955 10.1016/j.clinthera.2023.08.013

[45] Q. Zhou , R. Qin , Y. Yang , et al., “Polydatin Possesses Notable Anti?Osteoporotic Activity via Regulation of OPG, RANKL and β‐Catenin,” Molecular Medicine Reports 14 (2016): 1865–1869. 27357904 10.3892/mmr.2016.5432

[46] L. Lin , H. Gong , R. Li , et al., “Nanodrug With ROS and pH Dual‐Sensitivity Ameliorates Liver Fibrosis via Multicellular Regulation,” Advanced Science (Weinheim, Baden‐Württemberg, Germany) 7 (2020): 1903138. 32274310 10.1002/advs.201903138 PMC7140994

[47] S. Tang , Q. Tang , J. Jin , et al., “Polydatin Inhibits the IL‐1β‐Induced Inflammatory Response in Human Osteoarthritic Chondrocytes by Activating the Nrf2 Signaling Pathway and Ameliorates Murine Osteoarthritis,” Food & Function 9 (2018): 1701–1712. 29484338 10.1039/c7fo01555k

[48] C. Cremon , V. Stanghellini , M. R. Barbaro , et al., “Randomised Clinical Trial: The Analgesic Properties of Dietary Supplementation With Palmitoylethanolamide and Polydatin in Irritable Bowel Syndrome,” Alimentary Pharmacology & Therapeutics 45 (2017): 909–922. 28164346 10.1111/apt.13958

[49] K. Gulati , K. Gangele , N. Agarwal , M. Jamsandekar , D. Kumar , and K. M. Poluri , “Molecular Cloning and Biophysical Characterization of CXCL3 Chemokine,” International Journal of Biological Macromolecules 107 (2018): 575–584. 28928065 10.1016/j.ijbiomac.2017.09.032

[50] S. Wang , Y. Kuai , S. Lin , et al., “NF‐κB Activator 1 Downregulation in Macrophages Activates STAT3 to Promote Adenoma‐Adenocarcinoma Transition and Immunosuppression in Colorectal Cancer,” BMC Medicine 21 (2023): 115. 36978108 10.1186/s12916-023-02791-0 PMC10053426

[51] C. Fang , R. Zhong , S. Lu , et al., “TREM2 Promotes Macrophage Polarization From M1 to M2 and Suppresses Osteoarthritis Through the NF‐κB/CXCL3 Axis,” International Journal of Biological Sciences 20 (2024): 1992–2007. 38617547 10.7150/ijbs.91519 PMC11008261

[52] J. Guan , J. Weng , Q. Ren , et al., “Clinical Significance and Biological Functions of Chemokine CXCL3 in Head and Neck Squamous Cell Carcinoma,” Bioscience Reports 41 (2021): BSR20212403. 34870709 10.1042/BSR20212403 PMC8696619

[53] H. Wang , W. Chen , Y. Wang , et al., “SUB1 Promotes Colorectal Cancer Metastasis by Activating NF‐κB Signaling via UBR5‐Mediated Ubiquitination of UBXN1,” Science China. Life Sciences 67 (2024): 1199–1211. 38240906 10.1007/s11427-023-2429-5

[54] J. Wagner , L. M. Park , P. Mukhopadhyay , et al., “PCSK9 Inhibition Attenuates Alcohol‐Associated Neuronal Oxidative Stress and Cellular Injury,” Brain, Behavior, and Immunity 119 (2024): 494–506. 38657842 10.1016/j.bbi.2024.04.022

[55] C. R. Justus , M. A. Marie , E. J. Sanderlin , and L. V. Yang , “Transwell in Vitro Cell Migration and Invasion Assays,” Methods in Molecular Biology 2644 (2023): 349–359. 37142933 10.1007/978-1-0716-3052-5_22 PMC10335869

[56] W. Liu , F. Wu , X. Bi , et al., “Herbal Formula Jiawei Xiaochengqi Decoction Prevents Postoperative Abdominal Adhesion in a Rat Model Through Inhibition of CXCL2‐CXCR2 Pathway,” Phytomedicine 111 (2023): 154662. 36681054 10.1016/j.phymed.2023.154662

[57] A. Das , J. C. Chai , S. H. Kim , et al., “Dual RNA Sequencing Reveals the Expression of Unique Transcriptomic Signatures in Lipopolysaccharide‐Induced BV‐2 Microglial Cells,” PLoS One 10 (2015): e0121117. 25811458 10.1371/journal.pone.0121117 PMC4374676

[58] A. Das , S. H. Kim , S. Arifuzzaman , et al., “Transcriptome Sequencing Reveals That LPS‐Triggered Transcriptional Responses in Established Microglia BV2 Cell Lines Are Poorly Representative of Primary Microglia,” Journal of Neuroinflammation 13 (2016): 182. 27400875 10.1186/s12974-016-0644-1 PMC4940985

[59] X. Li , X. Yang , H. Lu , et al., “Calycosin Attenuates the Inflammatory Damage of Microglia Induced by Oxygen and Glucose Deprivation Through the HMGB1/TLR4/NF‐κB Signaling Pathway,” Acta Biochimica et Biophysica Sinica Shanghai 55 (2023): 1415–1424. 10.3724/abbs.2023125 PMC10520471 37528661

[60] H. Yeh , M. A. De Cruz , Y. You , et al., “Development and Characterization of in Vitro Inducible Immortalization of a Murine Microglia Cell Line for High Throughput Studies,” Scientific Reports 15 (2025): 3207. 39863723 10.1038/s41598-025-87543-1 PMC11762310

[61] Z. Li , M. Li , Z. Fang , and H. Wang , “Immunological Mechanisms and Therapeutic Strategies in Cerebral Ischemia‐Reperfusion Injury: From Inflammatory Response to Neurorepair,” International Journal of Molecular Sciences 26 (2025): 8336. 40943269 10.3390/ijms26178336 PMC12427725

[62] P. Yin , Y. Wei , X. Wang , M. Zhu , and J. Feng , “Roles of Specialized Pro‐Resolving Lipid Mediators in Cerebral Ischemia Reperfusion Injury,” Frontiers in Neurology 9 (2018): 617. 30131754 10.3389/fneur.2018.00617 PMC6090140

[63] H. Jin , Z. Li , S. Tan , et al., “Neutrophil Mobilization Triggers Microglial Functional Change to Exacerbate Cerebral Ischemia‐Reperfusion Injury,” Advanced Science (Weinheim, Baden‐Württemberg, Germany) 12 (2025): e03722. 40557450 10.1002/advs.202503722 PMC12462934

[64] J. Herz , P. Sabellek , T. E. Lane , M. Gunzer , D. M. Hermann , and T. R. Doeppner , “Role of Neutrophils in Exacerbation of Brain Injury After Focal Cerebral Ischemia in Hyperlipidemic Mice,” Stroke 46 (2015): 2916–2925. 26337969 10.1161/STROKEAHA.115.010620 PMC4589522

