# Supplementary material for: Polydatin Inhibits Neuroinflammation in Cerebral Ischemia–Reperfusion Injury Through Suppressing the CXCL3/CXCR2 Axis
Source: CNS Neurosci Ther. 2026 May 23;32(5):e70947. doi: 10.1002/cns.70947 (PMC13239370; doi:10.1002/cns.70947)
Supplement: Supplementary file 1 — Figure S1: Constitutional formula of Polydatin. Figure S2: Heatmap of gene expression related to the NF‐kappa B signaling pathway. Figure S3: PD reduces the number of CXCL3‐induced neutrophil migration. (A) MTT assay results showed that PD concentrations below 50 μM had no significant effect on the viability of neutrophils. (B, C) Crystal violet staining results showed that PD intervention reduced the number of migrating neutrophils. n = 6 for A, n = 3 for B, C. Data are presented as the mean ± SD. **p < 0.01, *p < 0.05. Table S1: Neurological function evaluation. Table S2: Primer sequence. Table S3: Common genes. Table S4: Statistical analysis of fluorescence intensity in rat brain tissues among groups in Figure 4G. [file CNS-32-e70947-s001.docx]

***Supplementary Materials***

**1 Reagents**

Ginaton (H20090365) was purchased from Dr. Willmar Schwabe GmbH & Co. KG (Karlsruhe, Germany). Polydatin (15721, Empirical formula: C_20_H_22_O_8_, Purity ≥95%, Constitutional formula was shown in Figure S1) was purchased from Merck China (Beijing, China). SOD assay kit (A001-3-2), MDA assay kit (A003-1-2), ROS assay kit (E004-1-1) were purchased from Nanjing Jiancheng Bioengineering Research Institute Co., LTD. ELISA kits for IL-1β (ml037361), IL-6 (ml102828), TNF-α (ml002859) were purchased from Shanghai Enzyme-linked Biotechnology Co., Ltd. (Shanghai, China). Recombinant Human CXCL3/GRO gamma Protein (277-GG) was purchased from R&D Systems China Co., Ltd (Shanghai, China). Primary antibodies: mouse anti-Iba1 (ab283319), rabbit anti-CXCL3 (ab220431), mouse anti-MPO (ab90810), rabbit anti- GAPDH (ab181602) were purchased from Abcam (Shanghai, China); rabbit anti-CXCR2 (PA5-102662) was purchased from Thermo Fisher Scientific (Shanghai, China). Secondary antibody for goat anti-mouse IgG H&L (ab6785) and goat anti-rabbit IgG H&L (ab207995) were purchased from Abcam (Shanghai, China). Total RNA extraction, first-strand cDNA reverse transcription, polymerase chain reaction (PCR) kits and primers were obtained from TianGen Biotechnology Co., Ltd. (Beijing, China).


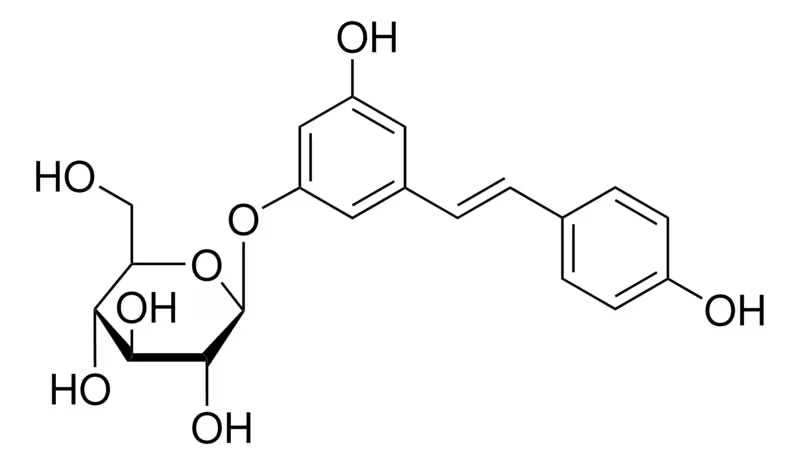


**Figure S1 Constitutional formula of Polydatin.**

**
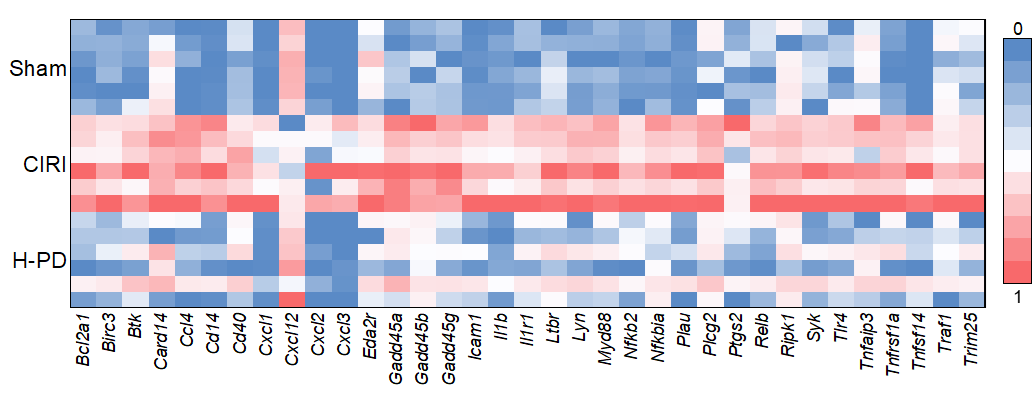
**

**Figure S2 Heatmap of gene expression related to the NF-kappa B signaling pathway.**

**
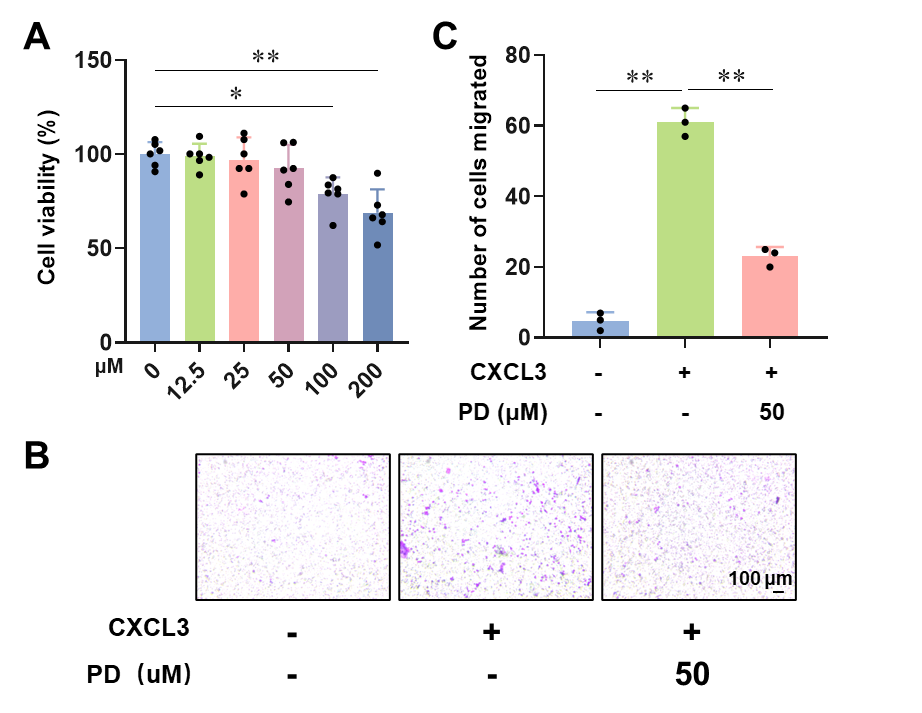
**

**Figure S3 PD reduces the number of CXCL3-induced neutrophil migration.**

(A) MTT assay results showed that PD concentrations below 50 μM had no significant effect on the viability of neutrophils. (B, C) Crystal violet staining results showed that PD intervention reduced the number of migrating neutrophils. n = 6 for A, n = 3 for B, C. Data are presented as the mean ± SD. ***P* < 0.01, **P* < 0.05.

**Table S1 Neurological function evaluation**

| Score | Bederson's scale score | Postural reflex test | Asymmetry score |
| --- | --- | --- | --- |
| 0 | No neurological functional impairment | Right forelimb showed ventral and lateral stretch | The front limb of rats with an unconstrained hanging freely brushed the edge of a table for 20 times.  $Asymmetry score=\frac{Left-Right}{Both+Left+Right}\times100\%$ |
| 1 | Continuous torso flexion to the right; | Right forelimb extended at an angle |  |
| 2 | Weakened resistance to contralateral push without turning | Right forelimb affixed to the chest with twisted body |  |
| 3 | Spontaneous circling around affected limbs under the condition of free activity | / |  |
| 4 | No spontaneous motor activity and occurring flaccid paralysis | / |  |

**Table S2 Primer sequence**

| Gene | Sequence (5'->3') | |
| --- | --- | --- |
| *Cxcl3* | Forward primer | CACCAGGCTACAGGGGC |
|  | Reverse primer | CAACCCTTGGTAGGGTGTTCA |
| *Cxcr2* | Forward primer | GCCCTGCCCATCTTAATTCTAC |
|  | Reverse primer | ACCCTCAAACGGGATGTATTGT |
| *Actb* | Forward primer | GTGACGTTGACATCCGTAAAGA |
|  | Reverse primer | GCCGGACTCATCGTACTCC |

**Table S3 Common genes**

| Gene name | CIRI vs Sham | | H-PD vs CIRI | |
| --- | --- | --- | --- | --- |
|  | log2FoldChange | padj | log2FoldChange | padj |
| Nuak2 | 1.738 | 0.000 | -1.277 | 0.003 |
| Antxr2 | 2.173 | 0.000 | -1.731 | 0.000 |
| Cplx2 | -1.970 | 0.000 | 1.592 | 0.004 |
| Ak2 | 1.908 | 0.000 | -1.356 | 0.000 |
| Phf24 | -1.165 | 0.000 | 1.010 | 0.003 |
| Glis1 | 1.598 | 0.001 | -1.382 | 0.003 |
| Pfkfb1 | 2.306 | 0.000 | -1.466 | 0.006 |
| Sqor | 1.632 | 0.000 | -1.152 | 0.002 |
| Tst | -1.257 | 0.000 | 1.098 | 0.001 |
| Csf2rb | 6.085 | 0.000 | -2.465 | 0.027 |
| Ccl7 | 9.621 | 0.000 | -4.091 | 0.023 |
| Tet1 | -1.789 | 0.000 | 1.292 | 0.000 |
| Prodh1 | -2.353 | 0.000 | 1.934 | 0.001 |
| Ermap | 2.545 | 0.002 | -2.332 | 0.033 |
| Nid2 | 1.372 | 0.012 | -1.729 | 0.005 |
| AABR07044900.1 | -2.005 | 0.003 | 1.629 | 0.021 |
| Srgn | 1.873 | 0.000 | -1.547 | 0.000 |
| Pln | -1.819 | 0.001 | 2.092 | 0.000 |
| Gpsm3 | 1.989 | 0.000 | -1.329 | 0.002 |
| Notch4 | 1.573 | 0.000 | -1.108 | 0.002 |
| Psmb8 | 2.895 | 0.000 | -1.745 | 0.001 |
| Tap1 | 1.874 | 0.000 | -1.533 | 0.000 |
| Psmb9 | 2.178 | 0.000 | -1.302 | 0.005 |
| Col11a2 | -2.847 | 0.000 | 1.893 | 0.000 |
| Kifc1 | 3.423 | 0.000 | -1.840 | 0.009 |
| Bak1 | 1.429 | 0.000 | -1.090 | 0.001 |
| Grm4 | -1.511 | 0.001 | 1.004 | 0.032 |
| Def6 | 1.359 | 0.000 | -1.051 | 0.011 |
| Cdkn1a | 2.818 | 0.000 | -1.674 | 0.001 |
| Pim1 | 1.835 | 0.000 | -1.150 | 0.019 |
| Tspyl4 | -1.379 | 0.000 | 1.120 | 0.000 |
| Sgpl1 | 1.193 | 0.000 | -1.007 | 0.002 |
| Slc16a10 | 1.670 | 0.016 | -1.306 | 0.049 |
| Lama4 | 1.684 | 0.000 | -1.481 | 0.000 |
| Bicc1 | 1.403 | 0.000 | -1.089 | 0.018 |
| Rtkn2 | -1.325 | 0.000 | 1.080 | 0.002 |
| Ptprc | 3.542 | 0.000 | -2.176 | 0.000 |
| Ung | 2.388 | 0.000 | -1.621 | 0.000 |
| Cmklr1 | 3.081 | 0.000 | -2.035 | 0.000 |
| RT1-CE5 | 2.714 | 0.000 | -1.689 | 0.009 |
| RT1-S3 | 1.047 | 0.001 | -1.027 | 0.004 |
| RT1-N3 | 2.040 | 0.000 | -1.602 | 0.000 |
| Smpdl3a | 1.937 | 0.000 | -1.169 | 0.011 |
| Ppp1r18 | 2.270 | 0.000 | -1.540 | 0.000 |
| Nrm | 1.857 | 0.000 | -1.250 | 0.001 |
| Dse | 1.816 | 0.000 | -1.439 | 0.002 |
| Calhm5 | 1.775 | 0.012 | -1.332 | 0.007 |
| Ier3 | 2.138 | 0.000 | -1.791 | 0.000 |
| Ncr3 | 3.301 | 0.000 | -1.986 | 0.009 |
| Lst1 | 6.263 | 0.000 | -3.410 | 0.000 |
| Msh5 | 3.242 | 0.000 | -1.698 | 0.023 |
| Sapcd1 | 1.711 | 0.000 | -1.146 | 0.003 |
| Medag | 2.063 | 0.000 | -1.854 | 0.001 |
| Alox5ap | 3.163 | 0.000 | -2.029 | 0.000 |
| Phkg1 | -1.828 | 0.000 | 1.828 | 0.001 |
| Arpc1b | 3.541 | 0.000 | -2.221 | 0.000 |
| Stxbp2 | 2.618 | 0.000 | -1.581 | 0.002 |
| Mmd2 | -1.500 | 0.000 | 1.234 | 0.003 |
| Cit | -1.322 | 0.000 | 1.094 | 0.002 |
| Pxn | 1.705 | 0.000 | -1.353 | 0.001 |
| Oasl | 2.199 | 0.000 | -2.779 | 0.000 |
| Hsf2bp | 1.834 | 0.003 | -1.471 | 0.028 |
| Trpv4 | 1.844 | 0.003 | -2.488 | 0.000 |
| Cstb | 1.710 | 0.000 | -1.229 | 0.000 |
| Itgb2 | 3.469 | 0.000 | -2.348 | 0.000 |
| Col18a1 | 2.311 | 0.000 | -1.793 | 0.000 |
| Gstt3 | -1.716 | 0.000 | 1.780 | 0.000 |
| Col6a1 | 1.591 | 0.000 | -1.124 | 0.010 |
| Col6a2 | 1.647 | 0.000 | -1.331 | 0.002 |
| Hvcn1 | 2.877 | 0.000 | -2.102 | 0.000 |
| S100b | -1.226 | 0.000 | 1.073 | 0.001 |
| Camkk2 | -2.102 | 0.000 | 1.632 | 0.002 |
| Mcm7 | 1.559 | 0.000 | -1.127 | 0.000 |
| Rph3a | -1.755 | 0.000 | 1.469 | 0.000 |
| Cyp3a62 | -1.933 | 0.000 | 1.404 | 0.009 |
| Slc8b1 | 1.279 | 0.000 | -1.009 | 0.020 |
| Serpine1 | 7.185 | 0.000 | -3.955 | 0.000 |
| Sh2b2 | 1.615 | 0.000 | -1.629 | 0.000 |
| Rasa4 | 3.023 | 0.000 | -1.668 | 0.017 |
| Tmem120a | 1.353 | 0.000 | -1.033 | 0.000 |
| Fzd9 | -1.452 | 0.000 | 1.303 | 0.005 |
| Cldn4 | 2.658 | 0.003 | -2.592 | 0.002 |
| Ncf1 | 3.360 | 0.000 | -2.181 | 0.000 |
| Slc8a2 | -1.661 | 0.000 | 1.146 | 0.002 |
| Cdca7 | 2.407 | 0.000 | -1.388 | 0.021 |
| Cd80 | 2.351 | 0.000 | -1.517 | 0.001 |
| Adamts1 | 3.761 | 0.000 | -2.464 | 0.000 |
| Chaf1b | 2.240 | 0.000 | -1.566 | 0.001 |
| Runx1 | 2.982 | 0.000 | -1.475 | 0.001 |
| Kalrn | -1.553 | 0.000 | 1.238 | 0.005 |
| LOC100360218 | 1.788 | 0.000 | -1.161 | 0.018 |
| Atp13a4 | -2.162 | 0.000 | 1.480 | 0.003 |
| Ece2 | -1.208 | 0.001 | 1.065 | 0.007 |
| Nrros | 2.092 | 0.000 | -1.667 | 0.000 |
| Fetub | 5.461 | 0.000 | -3.572 | 0.005 |
| Arntl2 | 3.748 | 0.000 | -1.885 | 0.008 |
| Mcm4 | 1.628 | 0.000 | -1.365 | 0.000 |
| Tmtc1 | -1.250 | 0.000 | 1.089 | 0.002 |
| Sdf2l1 | 2.059 | 0.000 | -1.018 | 0.015 |
| Klhl6 | 2.003 | 0.000 | -1.144 | 0.007 |
| Mx1 | 2.266 | 0.000 | -2.220 | 0.001 |
| Sult1d1 | -2.716 | 0.000 | 2.296 | 0.001 |
| Tmprss11f | 4.977 | 0.000 | -4.029 | 0.004 |
| Sidt1 | -1.362 | 0.000 | 1.166 | 0.002 |
| Gpat3 | 1.898 | 0.000 | -1.372 | 0.003 |
| Tlr6 | 1.721 | 0.000 | -1.497 | 0.002 |
| Dmp1 | 1.530 | 0.016 | -2.059 | 0.012 |
| Plac8 | 5.212 | 0.000 | -2.165 | 0.002 |
| Adcy5 | -1.048 | 0.000 | 1.149 | 0.013 |
| Aff1 | 1.301 | 0.000 | -1.100 | 0.001 |
| Sema5b | 1.014 | 0.011 | -1.023 | 0.039 |
| Art3 | -1.310 | 0.000 | 1.145 | 0.007 |
| Hlx | 2.679 | 0.000 | -1.952 | 0.000 |
| Atp10d | 1.792 | 0.000 | -1.307 | 0.001 |
| Gabra4 | -1.402 | 0.000 | 1.068 | 0.001 |
| Trim25 | 1.840 | 0.000 | -1.655 | 0.000 |
| Rasgef1b | -1.180 | 0.000 | 1.092 | 0.005 |
| Prkg2 | -2.019 | 0.000 | 1.281 | 0.024 |
| Prg4 | 3.087 | 0.000 | -2.296 | 0.002 |
| Serpinb8 | 6.237 | 0.000 | -3.367 | 0.000 |
| Rbm47 | 1.937 | 0.000 | -2.359 | 0.000 |
| Serpinb10 | 3.123 | 0.000 | -3.086 | 0.000 |
| Pctp | 2.391 | 0.000 | -1.406 | 0.001 |
| Tmem100 | 2.408 | 0.000 | -1.167 | 0.014 |
| Hlf | -2.194 | 0.000 | 1.993 | 0.001 |
| Nid1 | 1.121 | 0.010 | -1.280 | 0.009 |
| Ifi47 | 2.807 | 0.000 | -1.966 | 0.000 |
| Polq | 2.767 | 0.000 | -1.766 | 0.000 |
| AABR07058831.1 | 1.821 | 0.022 | -2.164 | 0.006 |
| Flt4 | 1.729 | 0.000 | -1.340 | 0.004 |
| Litaf | 1.844 | 0.000 | -1.369 | 0.001 |
| Ptgs2 | 1.893 | 0.000 | -1.474 | 0.013 |
| Rhoh | 2.834 | 0.000 | -1.641 | 0.001 |
| Dpp10 | -1.326 | 0.000 | 1.398 | 0.000 |
| Grap | 1.214 | 0.000 | -1.015 | 0.009 |
| Slc5a10 | 4.272 | 0.000 | -2.646 | 0.013 |
| Pla2g4a | 2.292 | 0.000 | -1.482 | 0.003 |
| Ciita | 2.966 | 0.000 | -2.359 | 0.005 |
| Lamc2 | 2.091 | 0.000 | -1.096 | 0.032 |
| Lamc1 | 1.513 | 0.000 | -1.328 | 0.000 |
| Nuf2 | 3.462 | 0.000 | -1.780 | 0.019 |
| Sele | 4.566 | 0.000 | -3.833 | 0.000 |
| Btc | 1.795 | 0.000 | -1.745 | 0.000 |
| Rgs5 | -1.652 | 0.000 | 1.239 | 0.006 |
| Adam11 | -1.869 | 0.000 | 1.387 | 0.005 |
| Areg | 3.005 | 0.018 | -2.802 | 0.040 |
| Npl | 2.215 | 0.000 | -1.161 | 0.002 |
| Sell | 5.842 | 0.000 | -3.604 | 0.000 |
| Cxcl2 | 6.203 | 0.000 | -5.284 | 0.000 |
| Selp | 7.993 | 0.000 | -3.641 | 0.043 |
| Cxcl1 | 7.840 | 0.000 | -4.619 | 0.000 |
| Gfpt2 | 1.277 | 0.001 | -1.018 | 0.028 |
| LOC24906 | 6.348 | 0.000 | -3.113 | 0.003 |
| Rbfox1 | -1.258 | 0.000 | 1.078 | 0.001 |
| Alb | -1.569 | 0.003 | 2.021 | 0.001 |
| Socs3 | 5.854 | 0.000 | -2.851 | 0.001 |
| Abcc3 | 1.940 | 0.000 | -1.390 | 0.007 |
| Lyl1 | 2.055 | 0.000 | -1.474 | 0.001 |
| Bst1 | 5.606 | 0.000 | -3.272 | 0.000 |
| Btbd17 | -3.073 | 0.000 | 2.462 | 0.001 |
| Fcrlb | 3.411 | 0.000 | -1.929 | 0.005 |
| Fcrla | 1.837 | 0.032 | -2.368 | 0.013 |
| Nlrp3 | 1.498 | 0.000 | -1.236 | 0.014 |
| Ercc6l | 4.400 | 0.000 | -2.000 | 0.003 |
| Lgals3bp | 1.664 | 0.000 | -1.517 | 0.000 |
| Trim16 | 1.830 | 0.000 | -1.135 | 0.009 |
| Ltc4s | -2.219 | 0.000 | 2.285 | 0.000 |
| Grin2c | -1.498 | 0.000 | 1.110 | 0.015 |
| Fancb | 1.890 | 0.000 | -1.299 | 0.004 |
| Cadm3 | -1.016 | 0.000 | 1.020 | 0.004 |
| Lypd1 | -1.188 | 0.024 | 1.191 | 0.042 |
| Mnda | 7.088 | 0.000 | -4.023 | 0.000 |
| Olr1585 | 5.338 | 0.000 | -3.920 | 0.003 |
| Clcn4 | -1.130 | 0.000 | 1.049 | 0.001 |
| Tnfrsf12a | 3.941 | 0.000 | -2.886 | 0.000 |
| Cybb | 4.088 | 0.000 | -2.864 | 0.000 |
| Pkmyt1 | 2.461 | 0.000 | -1.394 | 0.007 |
| Gabrb2 | -1.466 | 0.000 | 1.184 | 0.000 |
| Mcm6 | 2.873 | 0.000 | -1.877 | 0.001 |
| Fam184b | -1.581 | 0.000 | 1.564 | 0.004 |
| Bmx | 2.449 | 0.000 | -1.765 | 0.029 |
| Srpx2 | 2.510 | 0.000 | -1.915 | 0.004 |
| Batf3 | 2.350 | 0.000 | -1.976 | 0.001 |
| Ush2a | -2.918 | 0.000 | 1.772 | 0.028 |
| Cdkl5 | -1.843 | 0.000 | 1.354 | 0.001 |
| Atf3 | 5.286 | 0.000 | -3.641 | 0.000 |
| RGD1565785 | 4.081 | 0.000 | -1.755 | 0.002 |
| Tmem163 | -1.696 | 0.000 | 1.420 | 0.000 |
| Atp10b | -1.993 | 0.001 | 1.204 | 0.031 |
| Usp43 | -1.224 | 0.015 | 1.330 | 0.011 |
| Pttg1 | 3.593 | 0.000 | -1.822 | 0.018 |
| Gpr65 | 3.932 | 0.000 | -2.198 | 0.002 |
| Cxcr4 | 2.291 | 0.000 | -2.031 | 0.001 |
| Thsd7b | -2.015 | 0.000 | 1.502 | 0.006 |
| Rgs1 | 5.274 | 0.000 | -3.110 | 0.000 |
| Pi4k2b | 1.344 | 0.000 | -1.020 | 0.000 |
| Cd55 | 1.657 | 0.001 | -1.351 | 0.015 |
| Il2rg | 3.334 | 0.000 | -2.781 | 0.000 |
| Pfn1 | 1.505 | 0.000 | -1.065 | 0.000 |
| Phlda1 | 1.221 | 0.000 | -1.091 | 0.001 |
| Sh3pxd2b | 1.977 | 0.000 | -1.806 | 0.000 |
| Myo1c | 1.674 | 0.000 | -1.301 | 0.001 |
| Kcnc2 | -1.415 | 0.000 | 1.035 | 0.015 |
| Trib1 | 2.202 | 0.000 | -1.731 | 0.000 |
| Zfpm2 | -1.328 | 0.000 | 1.147 | 0.018 |
| Soat1 | 1.591 | 0.000 | -1.338 | 0.001 |
| Kcnv1 | -1.483 | 0.000 | 1.441 | 0.001 |
| Mgat4c | -1.253 | 0.001 | 1.019 | 0.007 |
| Hdac9 | -2.029 | 0.000 | 1.232 | 0.003 |
| Myo1a | 5.008 | 0.000 | -1.933 | 0.011 |
| Tafa2 | -1.267 | 0.002 | 1.480 | 0.017 |
| Arhgap30 | 3.411 | 0.000 | -2.274 | 0.000 |
| Dtl | 3.968 | 0.000 | -1.588 | 0.014 |
| Rims2 | -1.306 | 0.000 | 1.129 | 0.000 |
| Lrr1 | 5.380 | 0.000 | -2.445 | 0.003 |
| Stk10 | 2.261 | 0.000 | -1.950 | 0.000 |
| Lgr5 | -1.401 | 0.001 | 1.217 | 0.011 |
| Pole2 | 2.847 | 0.000 | -1.611 | 0.007 |
| Tlr7 | 2.252 | 0.000 | -1.535 | 0.000 |
| Dipk2b | 1.765 | 0.000 | -1.564 | 0.009 |
| Ifitm1 | 3.731 | 0.000 | -2.571 | 0.000 |
| Pah | -1.609 | 0.001 | 1.875 | 0.002 |
| Vipr2 | -1.790 | 0.000 | 1.447 | 0.000 |
| Ddc | -3.966 | 0.000 | 3.595 | 0.000 |
| Elk3 | 1.702 | 0.000 | -1.199 | 0.005 |
| Ddx39a | 2.128 | 0.000 | -1.624 | 0.000 |
| Tac4 | 3.210 | 0.000 | -2.152 | 0.008 |
| Sash3 | 1.879 | 0.000 | -1.312 | 0.002 |
| Mtbp | 2.728 | 0.000 | -1.523 | 0.003 |
| F11r | 1.425 | 0.000 | -1.279 | 0.003 |
| Map2k6 | -1.411 | 0.000 | 1.354 | 0.000 |
| Fignl1 | 2.898 | 0.000 | -1.404 | 0.018 |
| Sptlc3 | -5.808 | 0.000 | 5.052 | 0.001 |
| Ikzf1 | 1.780 | 0.000 | -1.370 | 0.001 |
| Sdr9c7 | -1.516 | 0.000 | 1.058 | 0.024 |
| Nek2 | 2.660 | 0.000 | -1.527 | 0.011 |
| Adgre5 | 1.655 | 0.000 | -1.402 | 0.000 |
| Scin | 4.140 | 0.007 | -4.496 | 0.014 |
| Myc | 3.164 | 0.000 | -2.257 | 0.000 |
| Lin7a | -1.077 | 0.003 | 1.339 | 0.007 |
| Dcn | 1.576 | 0.005 | -1.772 | 0.003 |
| Cthrc1 | 2.103 | 0.000 | -2.214 | 0.000 |
| Mb | 4.255 | 0.000 | -1.893 | 0.033 |
| Lum | 1.187 | 0.007 | -1.019 | 0.029 |
| Il1b | 4.712 | 0.000 | -3.776 | 0.000 |
| Creld2 | 1.851 | 0.000 | -1.397 | 0.000 |
| Fzd6 | 1.016 | 0.011 | -1.130 | 0.003 |
| Thbd | 2.256 | 0.000 | -1.841 | 0.001 |
| Cd244 | 1.167 | 0.004 | -1.123 | 0.014 |
| Nxt1 | 1.732 | 0.000 | -1.208 | 0.000 |
| Kcnj16 | -1.663 | 0.001 | 1.475 | 0.037 |
| Ano3 | -1.516 | 0.000 | 1.166 | 0.001 |
| Cytip | 3.632 | 0.000 | -2.837 | 0.000 |
| Fam171b | -1.154 | 0.000 | 1.021 | 0.000 |
| Cyp4f1 | -1.092 | 0.000 | 1.379 | 0.000 |
| Plcb1 | -1.345 | 0.000 | 1.085 | 0.001 |
| Limd1 | 1.461 | 0.000 | -1.036 | 0.012 |
| Has2 | 1.761 | 0.000 | -1.964 | 0.000 |
| Adcy8 | -2.123 | 0.000 | 1.184 | 0.039 |
| Kcna4 | -1.354 | 0.000 | 1.099 | 0.008 |
| Nusap1 | 3.709 | 0.000 | -1.728 | 0.016 |
| AABR07057765.1 | -1.836 | 0.000 | 1.313 | 0.002 |
| Ncapg2 | 2.009 | 0.000 | -1.437 | 0.001 |
| Upp1 | 3.496 | 0.000 | -2.175 | 0.000 |
| Arhgef25 | -1.304 | 0.000 | 1.086 | 0.011 |
| Kif18a | 3.365 | 0.000 | -1.684 | 0.027 |
| Ube2t | 3.531 | 0.000 | -2.001 | 0.025 |
| Tbr1 | -1.032 | 0.001 | 1.356 | 0.000 |
| Asf1b | 4.720 | 0.000 | -2.019 | 0.025 |
| Slc66a3 | 2.460 | 0.000 | -1.603 | 0.001 |
| Mapt | -1.331 | 0.000 | 1.092 | 0.000 |
| Kcnq3 | -1.766 | 0.000 | 1.153 | 0.003 |
| Plek | 2.104 | 0.000 | -1.350 | 0.004 |
| Hnmt | -1.405 | 0.000 | 1.343 | 0.001 |
| Shmt1 | 2.125 | 0.000 | -1.404 | 0.002 |
| Trhde | -1.340 | 0.000 | 1.054 | 0.011 |
| Itpka | -1.858 | 0.000 | 1.410 | 0.026 |
| Syne3 | 1.863 | 0.000 | -1.634 | 0.005 |
| Slc2a9 | 2.585 | 0.000 | -2.156 | 0.002 |
| Scn8a | -1.825 | 0.000 | 1.298 | 0.001 |
| Csdc2 | -1.292 | 0.000 | 1.235 | 0.001 |
| Galnt13 | -1.243 | 0.000 | 1.096 | 0.001 |
| Vsnl1 | -1.995 | 0.000 | 1.779 | 0.000 |
| Pamr1 | -1.291 | 0.000 | 1.239 | 0.002 |
| Elf4 | 3.007 | 0.000 | -1.839 | 0.003 |
| Csrnp3 | -1.379 | 0.000 | 1.187 | 0.003 |
| Kcnj3 | -1.214 | 0.000 | 1.038 | 0.003 |
| S1pr4 | 1.693 | 0.007 | -1.493 | 0.025 |
| Lyar | 1.450 | 0.000 | -1.039 | 0.000 |
| Gna15 | 1.590 | 0.000 | -1.074 | 0.010 |
| Ngfr | 2.449 | 0.000 | -2.214 | 0.002 |
| Rasgrp1 | -1.126 | 0.000 | 1.113 | 0.001 |
| Cdca7l | 2.154 | 0.000 | -1.386 | 0.015 |
| Odc1 | 1.476 | 0.000 | -1.198 | 0.001 |
| Pcsk2 | -1.312 | 0.000 | 1.135 | 0.000 |
| Lamp5 | -1.204 | 0.005 | 1.309 | 0.025 |
| Slc1a2 | -2.392 | 0.000 | 1.513 | 0.004 |
| Ybx3 | 1.742 | 0.000 | -1.489 | 0.002 |
| Grm3 | -1.213 | 0.000 | 1.099 | 0.001 |
| Adamts8 | 2.308 | 0.000 | -1.576 | 0.038 |
| Abi3 | 1.611 | 0.000 | -1.283 | 0.002 |
| Rpia | 1.119 | 0.000 | -1.000 | 0.007 |
| Tead4 | 2.167 | 0.000 | -1.826 | 0.000 |
| Gadd45a | 3.006 | 0.000 | -1.594 | 0.001 |
| Misp3 | 2.762 | 0.000 | -1.618 | 0.002 |
| Lcp2 | 2.641 | 0.000 | -1.597 | 0.004 |
| AABR07047872.1 | -1.422 | 0.000 | 1.244 | 0.006 |
| Ar | -2.432 | 0.000 | 1.535 | 0.041 |
| Fam110c | 3.636 | 0.000 | -3.046 | 0.000 |
| Runx1t1 | -1.088 | 0.000 | 1.202 | 0.001 |
| Mgp | 2.787 | 0.000 | -1.252 | 0.013 |
| Slc6a11 | -2.854 | 0.000 | 1.892 | 0.000 |
| Birc3 | 3.744 | 0.000 | -2.367 | 0.001 |
| Hacd4 | 2.181 | 0.000 | -1.797 | 0.006 |
| Ptpn7 | 2.000 | 0.000 | -1.071 | 0.007 |
| Arhgdib | 2.717 | 0.000 | -1.697 | 0.002 |
| Nlrc4 | 2.485 | 0.000 | -1.528 | 0.002 |
| Lyz2 | 4.632 | 0.000 | -3.132 | 0.000 |
| Lrrc3b | -1.297 | 0.000 | 1.042 | 0.009 |
| Il1rn | 4.760 | 0.000 | -3.318 | 0.000 |
| Fsip1 | 2.385 | 0.000 | -1.584 | 0.005 |
| LOC103690020 | 4.550 | 0.001 | -3.883 | 0.010 |
| Rln3 | 2.816 | 0.001 | -2.864 | 0.006 |
| Pawr | 2.102 | 0.000 | -1.490 | 0.002 |
| Nnmt | 1.506 | 0.000 | -1.031 | 0.003 |
| Mlip | -1.650 | 0.000 | 1.536 | 0.000 |
| A3galt2 | 4.393 | 0.000 | -2.220 | 0.004 |
| Foxm1 | 1.969 | 0.000 | -1.345 | 0.005 |
| Nr4a3 | -2.268 | 0.000 | 1.156 | 0.039 |
| Kcnma1 | -1.293 | 0.000 | 1.004 | 0.002 |
| Ezh2 | 1.724 | 0.000 | -1.225 | 0.002 |
| AABR07016841.1 | -1.278 | 0.000 | 1.282 | 0.000 |
| Psd4 | 2.221 | 0.000 | -1.615 | 0.001 |
| Cd44 | 4.511 | 0.000 | -2.379 | 0.000 |
| Gngt2 | 4.353 | 0.000 | -2.526 | 0.005 |
| Hk2 | 1.427 | 0.000 | -1.209 | 0.003 |
| Cntn3 | -1.118 | 0.005 | 1.175 | 0.004 |
| Reg3b | 5.560 | 0.000 | -4.428 | 0.000 |
| Rasal3 | 2.525 | 0.000 | -1.555 | 0.002 |
| Slc30a3 | -1.519 | 0.000 | 1.387 | 0.001 |
| Ifih1 | 1.216 | 0.000 | -1.081 | 0.001 |
| Pdia4 | 1.631 | 0.000 | -1.111 | 0.000 |
| Dsn1 | 2.024 | 0.000 | -1.487 | 0.001 |
| Gpr37l1 | -1.138 | 0.000 | 1.031 | 0.002 |
| Zbp1 | 2.809 | 0.002 | -2.342 | 0.012 |
| Ptges | 4.674 | 0.000 | -3.132 | 0.000 |
| Reg3a | 6.834 | 0.000 | -3.756 | 0.002 |
| Pygl | 1.440 | 0.000 | -1.248 | 0.004 |
| Akap5 | -1.573 | 0.001 | 1.283 | 0.021 |
| Cdk2 | 1.532 | 0.000 | -1.458 | 0.000 |
| Frmd3 | -1.360 | 0.000 | 1.096 | 0.002 |
| Sema3c | 1.170 | 0.001 | -1.121 | 0.016 |
| Slc6a1 | -2.028 | 0.000 | 1.428 | 0.000 |
| Cyfip2 | -1.347 | 0.000 | 1.181 | 0.001 |
| Reg3g | 10.172 | 0.000 | -3.700 | 0.045 |
| Meox2 | -4.443 | 0.000 | 3.874 | 0.031 |
| Fam163b | -1.267 | 0.000 | 1.489 | 0.000 |
| AABR07060487.1 | -1.796 | 0.000 | 1.336 | 0.000 |
| Ccr1 | 5.587 | 0.000 | -4.595 | 0.000 |
| Spc25 | 3.062 | 0.000 | -1.666 | 0.022 |
| Cdkn2b | 1.386 | 0.002 | -1.337 | 0.039 |
| Cst7 | 6.666 | 0.000 | -4.210 | 0.000 |
| Mmp19 | 3.616 | 0.000 | -1.795 | 0.002 |
| Klrg2 | 1.253 | 0.047 | -2.219 | 0.006 |
| Hdac11 | -1.182 | 0.000 | 1.039 | 0.000 |
| Baz1a | 2.246 | 0.000 | -1.593 | 0.001 |
| Pgr | -1.603 | 0.000 | 1.306 | 0.006 |
| Rbl1 | 3.244 | 0.000 | -1.759 | 0.005 |
| AABR07029272.1 | 2.129 | 0.000 | -1.504 | 0.008 |
| Ncf4 | 4.909 | 0.000 | -2.786 | 0.000 |
| Arhgap9 | 2.296 | 0.000 | -1.652 | 0.001 |
| Padi3 | 2.536 | 0.000 | -1.443 | 0.024 |
| Ano6 | 1.695 | 0.000 | -1.341 | 0.000 |
| Mms22l | 4.248 | 0.000 | -2.312 | 0.003 |
| Lif | 5.162 | 0.000 | -4.014 | 0.000 |
| Cntn5 | -1.768 | 0.000 | 1.320 | 0.007 |
| L3mbtl1 | -2.363 | 0.000 | 2.018 | 0.001 |
| Plin2 | 4.150 | 0.000 | -2.665 | 0.000 |
| Rin3 | 2.330 | 0.000 | -1.579 | 0.001 |
| Adhfe1 | -2.353 | 0.000 | 2.083 | 0.000 |
| Xdh | 3.096 | 0.000 | -2.257 | 0.000 |
| Lgmn | 2.490 | 0.000 | -1.764 | 0.000 |
| Fgb | 4.347 | 0.008 | -3.414 | 0.049 |
| Itpr1 | -1.250 | 0.000 | 1.282 | 0.004 |
| Cd8b | 6.807 | 0.000 | -4.188 | 0.000 |
| Ttpa | -2.079 | 0.000 | 1.149 | 0.003 |
| Cyp46a1 | -1.305 | 0.000 | 1.044 | 0.012 |
| Ccl2 | 9.338 | 0.000 | -4.475 | 0.010 |
| Cd8a | 7.161 | 0.000 | -4.111 | 0.007 |
| Flnc | 2.567 | 0.000 | -1.657 | 0.000 |
| Atp1a2 | -1.994 | 0.000 | 1.397 | 0.000 |
| Spdl1 | 2.232 | 0.000 | -1.543 | 0.003 |
| Fbn1 | 1.983 | 0.000 | -2.171 | 0.000 |
| Klrb1b | 3.762 | 0.000 | -2.323 | 0.026 |
| Trib3 | 3.732 | 0.000 | -3.133 | 0.000 |
| Rac2 | 3.540 | 0.000 | -1.835 | 0.004 |
| Casp1 | 2.454 | 0.000 | -1.670 | 0.000 |
| Nfkbia | 2.219 | 0.000 | -1.475 | 0.000 |
| Ebf4 | -1.338 | 0.000 | 1.134 | 0.000 |
| Dok1 | 2.184 | 0.000 | -1.823 | 0.000 |
| Ptgs1 | 1.206 | 0.001 | -1.077 | 0.031 |
| Unc13d | 1.827 | 0.000 | -1.357 | 0.001 |
| Klhl41 | 2.380 | 0.000 | -1.453 | 0.048 |
| Ccnf | 1.761 | 0.000 | -1.070 | 0.003 |
| Lrtm2 | -1.945 | 0.000 | 1.774 | 0.000 |
| Slc1a6 | -1.427 | 0.001 | 1.306 | 0.007 |
| Kcnh7 | -1.508 | 0.000 | 1.326 | 0.008 |
| Msc | 2.031 | 0.006 | -2.706 | 0.001 |
| Fhl3 | 2.596 | 0.000 | -1.766 | 0.000 |
| Il23r | 3.011 | 0.025 | -3.989 | 0.013 |
| Angptl4 | 2.939 | 0.000 | -1.776 | 0.000 |
| Plppr1 | -1.470 | 0.000 | 1.149 | 0.006 |
| Ehd4 | 1.774 | 0.000 | -1.337 | 0.000 |
| Igsf1 | -2.234 | 0.000 | 1.764 | 0.011 |
| Gabrr1 | 1.684 | 0.008 | -1.518 | 0.046 |
| Cadps2 | -1.958 | 0.000 | 1.716 | 0.000 |
| Kcnj9 | -1.375 | 0.000 | 1.063 | 0.007 |
| Sipa1l1 | -1.162 | 0.000 | 1.084 | 0.000 |
| Oprk1 | -2.230 | 0.001 | 2.192 | 0.007 |
| Cd63 | 2.734 | 0.000 | -1.450 | 0.001 |
| Cyth4 | 2.002 | 0.000 | -1.279 | 0.008 |
| Gria3 | -1.523 | 0.000 | 1.271 | 0.000 |
| Mcam | 1.096 | 0.022 | -1.184 | 0.015 |
| Mgst1 | 2.432 | 0.000 | -1.326 | 0.005 |
| Fam151a | -1.478 | 0.000 | 1.074 | 0.004 |
| Mybl2 | 4.325 | 0.000 | -2.274 | 0.005 |
| Gdf6 | 4.314 | 0.000 | -3.486 | 0.002 |
| Apold1 | 2.467 | 0.000 | -1.263 | 0.010 |
| Slc16a7 | -1.360 | 0.000 | 1.070 | 0.000 |
| Vrk2 | 1.800 | 0.000 | -1.250 | 0.004 |
| Clec2dl1 | 2.845 | 0.004 | -2.917 | 0.000 |
| Usp1 | 1.154 | 0.000 | -1.051 | 0.000 |
| Tbxas1 | 3.715 | 0.000 | -1.988 | 0.002 |
| Gsdma | 3.190 | 0.009 | -2.696 | 0.049 |
| Timp4 | -1.288 | 0.000 | 1.131 | 0.002 |
| Klhl23 | -1.174 | 0.000 | 1.085 | 0.000 |
| Sh3tc1 | 2.215 | 0.000 | -1.494 | 0.000 |
| Fos | 1.829 | 0.000 | -1.354 | 0.000 |
| Bend5 | -1.227 | 0.000 | 1.029 | 0.008 |
| Slamf9 | 4.620 | 0.000 | -3.283 | 0.000 |
| Plscr1 | 1.874 | 0.000 | -1.581 | 0.000 |
| Rgs6 | -1.362 | 0.000 | 1.311 | 0.000 |
| Btbd3 | -1.583 | 0.000 | 1.523 | 0.000 |
| Gins1 | 5.074 | 0.000 | -2.138 | 0.003 |
| Lrrc4 | -1.259 | 0.000 | 1.078 | 0.001 |
| Arhgap11a | 2.498 | 0.000 | -1.563 | 0.006 |
| Plpp3 | -1.548 | 0.000 | 1.097 | 0.000 |
| Mfng | 1.546 | 0.000 | -1.314 | 0.001 |
| Jhy | -1.286 | 0.022 | 1.592 | 0.010 |
| Nppb | 5.492 | 0.001 | -5.505 | 0.002 |
| Irf1 | 1.568 | 0.000 | -1.287 | 0.000 |
| Tox2 | -1.736 | 0.000 | 1.303 | 0.000 |
| Tpx2 | 2.220 | 0.000 | -1.225 | 0.019 |
| Slc24a2 | -1.489 | 0.001 | 1.341 | 0.003 |
| Lyn | 2.582 | 0.000 | -1.725 | 0.000 |
| Parp12 | 1.465 | 0.000 | -1.236 | 0.000 |
| Ky | -2.335 | 0.009 | 2.018 | 0.021 |
| Trim47 | 2.810 | 0.000 | -1.367 | 0.002 |
| Madcam1 | 1.096 | 0.042 | -1.212 | 0.037 |
| Tagln2 | 3.006 | 0.000 | -1.878 | 0.000 |
| Gjd2 | -1.999 | 0.000 | 1.418 | 0.023 |
| Gimap4 | 4.152 | 0.000 | -2.880 | 0.002 |
| Myo1f | 3.761 | 0.000 | -2.323 | 0.001 |
| Gprc5a | 4.652 | 0.000 | -3.283 | 0.000 |
| Gimap5 | 1.580 | 0.001 | -1.682 | 0.001 |
| LOC100359539 | 4.098 | 0.000 | -2.124 | 0.006 |
| Lrrc43 | -2.086 | 0.000 | 1.532 | 0.005 |
| Rbms1 | 1.691 | 0.000 | -1.361 | 0.000 |
| LOC103690019 | 3.839 | 0.000 | -2.022 | 0.009 |
| Csf3 | 7.378 | 0.000 | -4.836 | 0.027 |
| Foxs1 | 2.220 | 0.000 | -1.887 | 0.000 |
| Dusp15 | -1.629 | 0.000 | 1.232 | 0.001 |
| Nkiras1 | -1.304 | 0.000 | 1.242 | 0.003 |
| Aoc1 | 2.267 | 0.028 | -2.297 | 0.010 |
| Batf | 4.800 | 0.000 | -2.746 | 0.001 |
| Ttll9 | -1.503 | 0.000 | 1.408 | 0.012 |
| Steap4 | 1.896 | 0.002 | -2.153 | 0.005 |
| Emp1 | 4.031 | 0.000 | -2.639 | 0.000 |
| Loxl1 | 1.454 | 0.000 | -1.382 | 0.005 |
| Tssk3 | -2.154 | 0.027 | 2.619 | 0.007 |
| RGD1563365 | 1.185 | 0.000 | -1.098 | 0.000 |
| Ccn3 | -1.612 | 0.003 | 1.218 | 0.045 |
| Arhgap32 | -1.307 | 0.000 | 1.238 | 0.000 |
| Slamf8 | 2.813 | 0.000 | -1.878 | 0.000 |
| Col5a1 | 1.342 | 0.000 | -1.330 | 0.003 |
| Csf3r | 3.400 | 0.000 | -2.058 | 0.003 |
| Zfhx4 | -1.258 | 0.001 | 1.417 | 0.002 |
| Gpnmb | 6.026 | 0.000 | -2.823 | 0.000 |
| Ass1 | 1.492 | 0.002 | -1.270 | 0.047 |
| Orc1 | 1.950 | 0.000 | -1.452 | 0.000 |
| Ino80b | 2.397 | 0.002 | -1.829 | 0.039 |
| Fli1 | 1.730 | 0.000 | -1.096 | 0.015 |
| Plbd1 | 3.967 | 0.000 | -3.033 | 0.000 |
| Map3k6 | 3.594 | 0.000 | -2.158 | 0.000 |
| Ets1 | 1.270 | 0.000 | -1.201 | 0.003 |
| Diaph3 | 2.444 | 0.000 | -2.073 | 0.001 |
| Slco2a1 | 4.214 | 0.000 | -2.891 | 0.000 |
| Elmod1 | -1.344 | 0.000 | 1.307 | 0.000 |
| Pon3 | 2.003 | 0.005 | -2.114 | 0.002 |
| Zc3h12a | 2.168 | 0.000 | -1.994 | 0.000 |
| C1qtnf4 | -1.572 | 0.000 | 1.479 | 0.001 |
| Cidec | 4.171 | 0.000 | -2.353 | 0.003 |
| Fut4 | 1.317 | 0.002 | -1.796 | 0.000 |
| Dleu7 | -1.382 | 0.000 | 1.194 | 0.003 |
| C3ar1 | 2.845 | 0.000 | -2.151 | 0.000 |
| Epha2 | 1.529 | 0.000 | -1.293 | 0.003 |
| Cdk6 | 2.000 | 0.000 | -1.688 | 0.001 |
| Cnr2 | 2.713 | 0.000 | -1.623 | 0.001 |
| Fgf14 | -1.289 | 0.000 | 1.049 | 0.003 |
| Adra2c | -1.428 | 0.006 | 1.578 | 0.017 |
| Zwilch | 1.188 | 0.000 | -1.112 | 0.000 |
| Fstl3 | 1.979 | 0.000 | -1.425 | 0.005 |
| Nr1d1 | -1.460 | 0.000 | 1.060 | 0.000 |
| Hck | 3.765 | 0.000 | -2.450 | 0.000 |
| Knstrn | 3.995 | 0.000 | -2.011 | 0.012 |
| Cenpe | 2.623 | 0.000 | -1.344 | 0.028 |
| Fcnb | 6.694 | 0.000 | -3.523 | 0.002 |
| Nos3 | 1.664 | 0.000 | -1.379 | 0.001 |
| Tacr3 | -2.312 | 0.000 | 2.243 | 0.000 |
| Pik3cg | 2.040 | 0.000 | -1.342 | 0.008 |
| Sptssb | -2.639 | 0.000 | 2.458 | 0.000 |
| Lmo2 | 1.109 | 0.000 | -1.150 | 0.001 |
| Chn2 | -1.400 | 0.000 | 1.196 | 0.000 |
| Mcub | 4.142 | 0.000 | -2.769 | 0.000 |
| Mme | -1.243 | 0.000 | 1.432 | 0.024 |
| Erich3 | -1.305 | 0.000 | 1.099 | 0.001 |
| Kcnh5 | -1.001 | 0.015 | 1.502 | 0.004 |
| Rpe65 | -1.103 | 0.029 | 1.408 | 0.002 |
| Snai1 | 1.990 | 0.000 | -1.935 | 0.000 |
| Enpp6 | -2.212 | 0.000 | 1.589 | 0.006 |
| Grip2 | -1.462 | 0.000 | 1.151 | 0.002 |
| Akr1b8 | 2.456 | 0.000 | -1.684 | 0.023 |
| Slco1c1 | -1.152 | 0.000 | 1.066 | 0.004 |
| Cdh10 | -1.265 | 0.000 | 1.087 | 0.000 |
| Kirrel3 | -1.701 | 0.000 | 1.081 | 0.021 |
| Krt8 | 1.181 | 0.032 | -1.530 | 0.025 |
| Cdkn3 | 2.304 | 0.000 | -1.622 | 0.001 |
| Efcc1 | -1.123 | 0.023 | 1.170 | 0.016 |
| Tlr2 | 2.385 | 0.000 | -2.111 | 0.000 |
| Slc39a14 | 1.162 | 0.000 | -1.026 | 0.002 |
| Il18 | 2.367 | 0.000 | -1.946 | 0.000 |
| Olfm1 | -1.199 | 0.000 | 1.022 | 0.002 |
| Tmem88 | 2.200 | 0.000 | -1.647 | 0.000 |
| Crtapl1 | 1.727 | 0.000 | -1.395 | 0.003 |
| Fgr | 4.733 | 0.000 | -2.806 | 0.000 |
| Acod1 | 5.852 | 0.000 | -4.209 | 0.001 |
| Otof | -3.856 | 0.000 | 3.327 | 0.000 |
| Ripply2 | -1.007 | 0.030 | 1.118 | 0.012 |
| Osbpl3 | -1.247 | 0.000 | 1.154 | 0.014 |
| Clec4a3 | 3.731 | 0.000 | -2.358 | 0.000 |
| Vtn | -1.352 | 0.000 | 1.155 | 0.003 |
| Clec4a | 1.712 | 0.008 | -1.328 | 0.049 |
| Pkp1 | 1.227 | 0.001 | -1.158 | 0.028 |
| Cldn10 | -1.854 | 0.000 | 1.623 | 0.000 |
| S100a11 | 4.882 | 0.000 | -2.531 | 0.001 |
| Clec4d | 7.961 | 0.000 | -3.687 | 0.001 |
| Cald1 | 1.551 | 0.000 | -1.041 | 0.016 |
| Serpini1 | -1.377 | 0.000 | 1.609 | 0.000 |
| Ada | 2.601 | 0.000 | -1.746 | 0.002 |
| Cd180 | 1.832 | 0.000 | -1.374 | 0.004 |
| Smc4 | 1.800 | 0.000 | -1.205 | 0.003 |
| Slc17a9 | 1.985 | 0.000 | -1.745 | 0.000 |
| Il6 | 5.811 | 0.000 | -3.787 | 0.000 |
| Pde8b | -1.106 | 0.000 | 1.298 | 0.000 |
| Ninj2 | -1.366 | 0.012 | 1.175 | 0.015 |
| Slc7a7 | 2.941 | 0.000 | -2.361 | 0.000 |
| Dzip1 | -1.210 | 0.000 | 1.051 | 0.000 |
| Lcp1 | 3.372 | 0.000 | -2.217 | 0.000 |
| Ptger3 | 1.161 | 0.037 | -1.636 | 0.011 |
| Ctsb | 1.746 | 0.000 | -1.018 | 0.001 |
| Dnajc3 | 1.236 | 0.000 | -1.102 | 0.000 |
| Anxa2 | 4.006 | 0.000 | -2.713 | 0.000 |
| Ccdc180 | -1.075 | 0.024 | 1.378 | 0.018 |
| Gnal | -1.725 | 0.000 | 2.103 | 0.006 |
| Ptbp1 | 1.903 | 0.000 | -1.504 | 0.000 |
| Hes2 | 2.692 | 0.000 | -1.986 | 0.013 |
| RGD1305807 | 5.073 | 0.000 | -3.240 | 0.003 |
| Tfpi2 | 4.627 | 0.000 | -3.866 | 0.000 |
| Plau | 5.456 | 0.000 | -3.018 | 0.000 |
| Tlr4 | 2.622 | 0.000 | -2.021 | 0.000 |
| Thbs2 | 1.836 | 0.001 | -1.605 | 0.012 |
| Tspo | 5.381 | 0.000 | -1.972 | 0.007 |
| Tmem123 | 1.544 | 0.000 | -1.142 | 0.005 |
| Sphk1 | 3.925 | 0.000 | -2.896 | 0.000 |
| Prcp | 1.554 | 0.000 | -1.380 | 0.000 |
| Lgals3 | 7.150 | 0.000 | -2.835 | 0.007 |
| Ccr7 | 2.207 | 0.006 | -2.425 | 0.013 |
| Cmtm3 | 1.997 | 0.000 | -1.323 | 0.003 |
| Depdc1b | 2.412 | 0.000 | -1.552 | 0.024 |
| Dlgap5 | 3.744 | 0.000 | -2.171 | 0.004 |
| Gpm6a | -1.470 | 0.000 | 1.308 | 0.001 |
| Dap | 1.853 | 0.000 | -1.130 | 0.015 |
| Tp53 | 1.357 | 0.000 | -1.127 | 0.000 |
| E2f5 | 1.327 | 0.000 | -1.042 | 0.000 |
| Scg3 | -1.241 | 0.000 | 1.087 | 0.000 |
| Fabp4 | 6.418 | 0.000 | -4.675 | 0.000 |
| Dlx6 | -1.034 | 0.009 | 1.271 | 0.014 |
| Mthfd2 | 1.799 | 0.000 | -1.600 | 0.000 |
| Mpped1 | -1.663 | 0.000 | 1.303 | 0.009 |
| Tmem154 | 2.529 | 0.000 | -1.476 | 0.006 |
| Cebpa | 1.992 | 0.000 | -1.617 | 0.000 |
| Slc7a10 | -1.951 | 0.000 | 1.582 | 0.000 |
| Tifa | 1.888 | 0.000 | -1.023 | 0.023 |
| Mmp14 | 2.034 | 0.000 | -1.971 | 0.000 |
| Cmtm6 | 1.982 | 0.000 | -1.674 | 0.000 |
| Itgb1 | 1.330 | 0.000 | -1.054 | 0.003 |
| Cmtm7 | 1.940 | 0.000 | -1.613 | 0.001 |
| Has1 | 2.273 | 0.000 | -1.980 | 0.002 |
| Rims1 | -1.607 | 0.000 | 1.179 | 0.001 |
| Gch1 | 3.092 | 0.000 | -2.048 | 0.000 |
| Laptm5 | 1.867 | 0.000 | -1.482 | 0.002 |
| Nt5e | 1.807 | 0.000 | -1.120 | 0.002 |
| Calcb | 6.123 | 0.000 | -4.266 | 0.001 |
| Calca | 5.200 | 0.000 | -1.966 | 0.008 |
| Il17ra | 1.611 | 0.000 | -1.318 | 0.001 |
| Wdhd1 | 2.398 | 0.000 | -1.572 | 0.001 |
| Bhmt | -1.931 | 0.003 | 1.740 | 0.024 |
| Ccl3 | 5.650 | 0.000 | -3.798 | 0.000 |
| Blm | 1.355 | 0.000 | -1.026 | 0.007 |
| Atp1b2 | -1.069 | 0.001 | 1.233 | 0.001 |
| Cntnap4 | -1.326 | 0.000 | 1.112 | 0.003 |
| Inmt | 3.919 | 0.000 | -2.438 | 0.002 |
| Hcrtr2 | -1.280 | 0.002 | 1.042 | 0.021 |
| Trmt9b | -1.831 | 0.000 | 1.308 | 0.004 |
| Sgms2 | 1.403 | 0.002 | -1.295 | 0.006 |
| Cenpn | 1.536 | 0.000 | -1.057 | 0.000 |
| Pde10a | -1.376 | 0.000 | 1.399 | 0.010 |
| Fam167a | 3.394 | 0.000 | -1.966 | 0.007 |
| Rftn1 | 1.116 | 0.013 | -1.380 | 0.004 |
| Lgi3 | -1.309 | 0.002 | 1.261 | 0.001 |
| Clspn | 1.538 | 0.002 | -1.360 | 0.001 |
| Ehd2 | 2.225 | 0.000 | -1.288 | 0.000 |
| Furin | 1.358 | 0.000 | -1.211 | 0.000 |
| Shbg | -1.063 | 0.000 | 1.137 | 0.000 |
| Reep4 | 2.025 | 0.000 | -1.223 | 0.003 |
| Acsbg1 | -1.178 | 0.000 | 1.152 | 0.001 |
| Ccl4 | 6.557 | 0.000 | -3.341 | 0.000 |
| Nkain1 | 1.793 | 0.000 | -1.178 | 0.001 |
| Aldoc | -1.341 | 0.000 | 1.375 | 0.000 |
| Rhbdf2 | 3.127 | 0.000 | -2.312 | 0.000 |
| S100a9 | 5.209 | 0.000 | -2.741 | 0.040 |
| Dusp26 | 1.664 | 0.000 | -1.056 | 0.002 |
| Filip1 | -1.976 | 0.000 | 1.518 | 0.014 |
| Coa8 | -3.116 | 0.006 | 2.130 | 0.018 |
| Rspo3 | 1.826 | 0.000 | -1.076 | 0.016 |
| Adamts18 | -1.603 | 0.000 | 1.017 | 0.030 |
| S100a6 | 4.892 | 0.000 | -2.054 | 0.001 |
| Plk4 | 2.493 | 0.000 | -1.559 | 0.002 |
| Fes | 3.253 | 0.000 | -2.117 | 0.001 |
| Fbxo34 | -1.047 | 0.000 | 1.087 | 0.002 |
| Degs2 | -1.119 | 0.005 | 1.947 | 0.000 |
| Elf1 | 1.358 | 0.000 | -1.107 | 0.007 |
| Fblim1 | 3.411 | 0.000 | -2.346 | 0.000 |
| Acpp | 2.404 | 0.000 | -2.428 | 0.003 |
| Lzts1 | -1.217 | 0.001 | 1.063 | 0.004 |
| Rrad | 2.084 | 0.000 | -1.752 | 0.000 |
| Sgtb | -1.378 | 0.000 | 1.166 | 0.005 |
| Krt12 | 2.117 | 0.006 | -1.568 | 0.039 |
| Iqgap1 | 2.040 | 0.000 | -1.709 | 0.000 |
| Sox7 | 2.727 | 0.000 | -2.541 | 0.000 |
| Ncaph | 2.650 | 0.000 | -1.659 | 0.006 |
| LOC685849 | -2.535 | 0.015 | 2.580 | 0.039 |
| Ifngr1 | 1.431 | 0.000 | -1.024 | 0.003 |
| Txn1 | 1.486 | 0.000 | -1.000 | 0.001 |
| Ltbp2 | 3.041 | 0.000 | -1.914 | 0.012 |
| Otulinl | 3.204 | 0.000 | -2.529 | 0.000 |
| Ostf1 | 1.490 | 0.000 | -1.204 | 0.000 |
| Syk | 2.087 | 0.000 | -1.745 | 0.001 |
| Spi1 | 3.113 | 0.000 | -1.989 | 0.000 |
| Asah2 | -1.127 | 0.001 | 1.193 | 0.000 |
| Itgb7 | 6.029 | 0.000 | -3.781 | 0.000 |
| Nthl1 | 1.481 | 0.000 | -1.026 | 0.000 |
| Baiap2l2 | -1.198 | 0.000 | 1.226 | 0.005 |
| Tgfbi | 4.580 | 0.000 | -3.346 | 0.000 |
| Skap2 | 1.953 | 0.000 | -1.297 | 0.002 |
| Ptx3 | 3.681 | 0.000 | -1.862 | 0.032 |
| Gchfr | 2.194 | 0.000 | -1.417 | 0.001 |
| Gucy1a1 | -1.979 | 0.000 | 2.150 | 0.000 |
| Aspm | 2.680 | 0.000 | -1.454 | 0.037 |
| Casp8 | 2.154 | 0.000 | -1.556 | 0.001 |
| Cacng3 | -2.014 | 0.000 | 1.688 | 0.002 |
| Npw | 2.590 | 0.017 | -2.926 | 0.015 |
| Bid | 1.951 | 0.000 | -1.184 | 0.000 |
| Slc39a8 | 1.198 | 0.000 | -1.006 | 0.019 |
| Mcm3 | 4.288 | 0.000 | -2.158 | 0.003 |
| Eif4ebp1 | 2.335 | 0.000 | -1.575 | 0.000 |
| Mmel1 | -1.013 | 0.000 | 1.118 | 0.007 |
| Lpxn | 1.848 | 0.000 | -1.182 | 0.001 |
| Rhoc | 2.582 | 0.000 | -1.384 | 0.000 |
| Ppp1r14d | -1.341 | 0.026 | 1.342 | 0.037 |
| Vamp5 | 2.249 | 0.000 | -1.343 | 0.000 |
| Postn | 3.655 | 0.000 | -2.541 | 0.003 |
| Trim55 | 2.644 | 0.000 | -1.628 | 0.002 |
| Vamp8 | 1.614 | 0.000 | -1.128 | 0.000 |
| Cklf | 2.028 | 0.000 | -1.691 | 0.000 |
| Slc25a43 | 2.479 | 0.000 | -1.123 | 0.013 |
| Ak8 | -2.128 | 0.000 | 1.435 | 0.018 |
| Msr1 | 7.164 | 0.000 | -3.403 | 0.002 |
| C1qc | 3.096 | 0.000 | -1.136 | 0.011 |
| Gdnf | 4.070 | 0.000 | -1.714 | 0.044 |
| Espl1 | 3.356 | 0.000 | -1.618 | 0.050 |
| Parp3 | 3.149 | 0.000 | -1.315 | 0.021 |
| Uaca | 1.412 | 0.000 | -1.183 | 0.001 |
| Fgl2 | 4.110 | 0.000 | -2.019 | 0.001 |
| Maff | 4.906 | 0.000 | -1.980 | 0.002 |
| Abca4 | 1.066 | 0.020 | -1.475 | 0.007 |
| Mov10 | 1.267 | 0.000 | -1.160 | 0.001 |
| Tgm2 | 3.271 | 0.000 | -2.161 | 0.000 |
| Themis2 | 3.114 | 0.000 | -2.146 | 0.000 |
| Lix1 | -2.853 | 0.000 | 2.115 | 0.000 |
| Gpr165 | -2.305 | 0.000 | 1.879 | 0.001 |
| Cyba | 3.227 | 0.000 | -2.147 | 0.000 |
| Eda2r | 1.799 | 0.024 | -2.124 | 0.019 |
| Tram2 | 1.565 | 0.000 | -1.624 | 0.002 |
| Prc1 | 3.641 | 0.000 | -1.941 | 0.013 |
| Sapcd2 | 1.553 | 0.000 | -1.310 | 0.001 |
| Wt1 | 4.351 | 0.000 | -4.367 | 0.001 |
| Gadd45g | 2.935 | 0.000 | -2.194 | 0.000 |
| Pcdh8 | -1.473 | 0.001 | 1.014 | 0.047 |
| Rad54l | 1.969 | 0.000 | -1.465 | 0.000 |
| Hmgb2 | 2.879 | 0.000 | -1.433 | 0.019 |
| Il13ra1 | 2.617 | 0.000 | -1.656 | 0.001 |
| Nr1h3 | 2.090 | 0.000 | -1.483 | 0.000 |
| Map3k1 | 1.333 | 0.005 | -1.437 | 0.003 |
| Tinagl1 | 2.515 | 0.000 | -1.524 | 0.003 |
| Rnaset2 | 2.129 | 0.000 | -1.089 | 0.005 |
| Arhgap45 | 2.428 | 0.000 | -1.763 | 0.001 |
| Scrg1 | -1.656 | 0.000 | 1.037 | 0.001 |
| Ptger4 | 2.233 | 0.000 | -1.678 | 0.002 |
| Vax2 | 2.964 | 0.002 | -2.311 | 0.016 |
| Tgfbr2 | 1.384 | 0.000 | -1.252 | 0.001 |
| Mctp1 | -1.094 | 0.000 | 1.125 | 0.014 |
| Arg1 | 2.430 | 0.000 | -2.273 | 0.001 |
| Pcdh20 | -1.795 | 0.000 | 1.374 | 0.011 |
| Pik3ap1 | 2.361 | 0.000 | -1.868 | 0.000 |
| Kcnt2 | -1.779 | 0.000 | 1.078 | 0.007 |
| Pola1 | 1.412 | 0.000 | -1.185 | 0.002 |
| Cdh5 | 1.192 | 0.003 | -1.115 | 0.018 |
| Rbpms | 2.200 | 0.000 | -1.480 | 0.002 |
| Tpcn2 | 1.057 | 0.000 | -1.020 | 0.002 |
| Rgs7bp | -1.220 | 0.000 | 1.040 | 0.002 |
| Minar1 | -1.637 | 0.000 | 1.767 | 0.001 |
| Npas2 | -1.054 | 0.002 | 1.069 | 0.005 |
| Pde7b | -1.202 | 0.000 | 1.290 | 0.002 |
| Gsta1 | -1.095 | 0.019 | 1.260 | 0.018 |
| Rassf4 | 2.697 | 0.000 | -1.804 | 0.000 |
| Sh2d4a | 1.216 | 0.009 | -1.435 | 0.006 |
| Slc6a12 | 1.655 | 0.041 | -1.723 | 0.017 |
| Treml2 | 1.565 | 0.023 | -2.108 | 0.013 |
| Dok3 | 3.560 | 0.000 | -1.838 | 0.001 |
| Kdelr3 | 1.788 | 0.000 | -1.354 | 0.001 |
| Cxcl12 | -1.303 | 0.000 | 1.313 | 0.003 |
| Melk | 4.868 | 0.000 | -1.827 | 0.029 |
| Dntt | 3.008 | 0.000 | -2.564 | 0.005 |
| Slc31a2 | 2.214 | 0.000 | -1.727 | 0.000 |
| Myd88 | 2.358 | 0.000 | -1.722 | 0.000 |
| Myo7a | 2.282 | 0.000 | -1.632 | 0.005 |
| Capg | 4.684 | 0.000 | -2.038 | 0.004 |
| Cps1 | -1.338 | 0.000 | 1.092 | 0.010 |
| Ndc80 | 3.145 | 0.000 | -1.634 | 0.023 |
| Ppp4r1 | 1.580 | 0.000 | -1.300 | 0.000 |
| Sec61a1 | 1.245 | 0.000 | -1.054 | 0.000 |
| Ctrc | 1.700 | 0.000 | -1.128 | 0.012 |
| Sh3bp2 | 1.809 | 0.000 | -1.751 | 0.000 |
| Trem3 | 6.151 | 0.000 | -3.755 | 0.000 |
| Folh1 | -1.927 | 0.000 | 1.469 | 0.000 |
| Lmnb1 | 1.996 | 0.000 | -1.426 | 0.003 |
| Kcnq5 | -1.825 | 0.000 | 1.734 | 0.000 |
| Enpp3 | 4.195 | 0.000 | -3.152 | 0.000 |
| Rbp1 | 2.967 | 0.000 | -1.813 | 0.004 |
| Tnip2 | 2.240 | 0.000 | -1.635 | 0.000 |
| Ubtd1 | 1.401 | 0.000 | -1.174 | 0.000 |
| Arhgap17 | 1.543 | 0.000 | -1.075 | 0.002 |
| Hes5 | -3.495 | 0.000 | 2.607 | 0.000 |
| Dusp2 | 1.989 | 0.000 | -2.073 | 0.000 |
| Kcnj4 | -1.624 | 0.000 | 1.482 | 0.001 |
| Fyb1 | 2.418 | 0.000 | -1.403 | 0.011 |
| Gpr132 | 3.624 | 0.000 | -2.636 | 0.000 |
| Nefm | -1.258 | 0.000 | 1.114 | 0.016 |
| Dok2 | 2.795 | 0.000 | -2.050 | 0.000 |
| Nox4 | 3.694 | 0.002 | -2.277 | 0.047 |
| Dsp | 1.770 | 0.001 | -1.562 | 0.016 |
| Ankrd34c | -1.737 | 0.000 | 1.565 | 0.003 |
| Itpkc | 1.345 | 0.000 | -1.101 | 0.000 |
| Zc3hav1 | 1.839 | 0.000 | -1.388 | 0.000 |
| Il6st | 1.037 | 0.000 | -1.112 | 0.001 |
| Cdt1 | 3.844 | 0.000 | -2.217 | 0.000 |
| Sbno2 | 3.110 | 0.000 | -2.017 | 0.000 |
| Dll4 | 1.626 | 0.000 | -1.442 | 0.002 |
| St8sia1 | -1.109 | 0.000 | 1.012 | 0.011 |
| Kif23 | 2.564 | 0.000 | -1.770 | 0.001 |
| Hmox1 | 5.890 | 0.000 | -3.960 | 0.000 |
| Nod2 | 1.972 | 0.003 | -2.156 | 0.003 |
| Evi2b | 2.328 | 0.000 | -1.422 | 0.003 |
| Cks2 | 3.880 | 0.000 | -1.926 | 0.017 |
| Fut7 | 2.084 | 0.000 | -1.409 | 0.015 |
| Paqr5 | -1.001 | 0.000 | 1.104 | 0.000 |
| Manf | 1.568 | 0.000 | -1.002 | 0.000 |
| Snx20 | 1.927 | 0.000 | -1.592 | 0.000 |
| Klf2 | 1.503 | 0.000 | -1.293 | 0.000 |
| Plch2 | -1.851 | 0.000 | 1.502 | 0.012 |
| Ece1 | 1.838 | 0.000 | -1.421 | 0.001 |
| Erbb4 | -1.883 | 0.000 | 1.311 | 0.000 |
| Rab32 | 3.766 | 0.000 | -2.026 | 0.002 |
| Cxcr2 | 6.098 | 0.000 | -3.447 | 0.010 |
| Cebpe | 4.607 | 0.000 | -4.048 | 0.000 |
| Fn1 | 3.547 | 0.000 | -1.909 | 0.000 |
| Ptpn6 | 3.101 | 0.000 | -1.849 | 0.000 |
| Syt10 | -1.485 | 0.008 | 1.132 | 0.021 |
| Dlgap3 | -1.384 | 0.000 | 1.232 | 0.010 |
| Slc39a4 | 3.317 | 0.000 | -2.158 | 0.025 |
| Inhba | 1.815 | 0.000 | -1.347 | 0.011 |
| Csrp3 | 7.130 | 0.000 | -3.916 | 0.006 |
| Mcm5 | 4.334 | 0.000 | -2.186 | 0.005 |
| Ccn1 | 4.464 | 0.000 | -2.919 | 0.000 |
| Gja4 | 2.359 | 0.000 | -1.779 | 0.000 |
| Edn1 | 2.121 | 0.000 | -1.052 | 0.013 |
| Trim66 | -1.465 | 0.000 | 1.410 | 0.003 |
| Il1r2 | 6.371 | 0.000 | -4.448 | 0.000 |
| Scara5 | 2.051 | 0.000 | -2.070 | 0.000 |
| Qrfpr | -2.398 | 0.000 | 3.178 | 0.000 |
| Lox | 3.323 | 0.000 | -1.701 | 0.049 |
| Cttnbp2nl | 1.320 | 0.000 | -1.038 | 0.000 |
| Il1r1 | 2.287 | 0.000 | -1.551 | 0.001 |
| Pmfbp1 | 1.290 | 0.011 | -1.269 | 0.018 |
| Plcxd3 | -1.209 | 0.019 | 1.097 | 0.021 |
| Hnrnpf | 1.309 | 0.000 | -1.018 | 0.000 |
| Entpd1 | 2.343 | 0.000 | -1.691 | 0.015 |
| Anpep | 3.578 | 0.000 | -2.733 | 0.000 |
| Efnb2 | -1.053 | 0.001 | 1.186 | 0.000 |
| Arl11 | 3.818 | 0.000 | -2.681 | 0.000 |
| Zfp385d | -1.896 | 0.000 | 1.761 | 0.000 |
| Tonsl | 1.835 | 0.000 | -1.271 | 0.001 |
| Rasd2 | -1.397 | 0.000 | 2.184 | 0.001 |
| Adcy7 | 2.880 | 0.000 | -2.063 | 0.001 |
| Mapkapk3 | 1.593 | 0.000 | -1.229 | 0.000 |
| Il1rl1 | 2.078 | 0.000 | -2.111 | 0.001 |
| Emilin2 | 3.217 | 0.000 | -2.843 | 0.000 |
| Rassf10 | -2.007 | 0.000 | 1.238 | 0.030 |
| Rnf152 | -2.061 | 0.000 | 2.115 | 0.000 |
| Sec24d | 1.388 | 0.000 | -1.112 | 0.001 |
| Ttc7a | 1.555 | 0.000 | -1.336 | 0.000 |
| Adgrg3 | 3.299 | 0.001 | -2.017 | 0.016 |
| Stx11 | 3.096 | 0.000 | -2.985 | 0.000 |
| Ifitm2 | 1.887 | 0.000 | -1.388 | 0.004 |
| Osgin1 | 2.517 | 0.000 | -2.178 | 0.001 |
| Slc11a1 | 3.146 | 0.000 | -2.664 | 0.000 |
| Bard1 | 3.102 | 0.000 | -1.725 | 0.007 |
| Pdpn | 3.208 | 0.000 | -1.421 | 0.016 |
| Hp | 2.938 | 0.000 | -2.102 | 0.002 |
| Zfp483 | -1.390 | 0.000 | 1.041 | 0.003 |
| Mdfi | 2.457 | 0.000 | -1.971 | 0.000 |
| M6pr | 2.006 | 0.001 | -1.823 | 0.007 |
| Abhd15 | 1.725 | 0.000 | -1.223 | 0.012 |
| Il18r1 | 2.619 | 0.001 | -2.820 | 0.001 |
| Ptgr1 | 2.127 | 0.000 | -1.592 | 0.002 |
| Ifitm3 | 3.042 | 0.000 | -1.503 | 0.007 |
| Nlrp10 | 4.534 | 0.001 | -3.118 | 0.024 |
| Mocos | 2.661 | 0.000 | -1.370 | 0.014 |
| Ube2c | 4.493 | 0.000 | -1.983 | 0.025 |
| Nrg4 | 3.078 | 0.000 | -2.277 | 0.002 |
| Pkp3 | 2.168 | 0.000 | -1.756 | 0.009 |
| Gal | 3.792 | 0.000 | -3.186 | 0.000 |
| Gem | 1.851 | 0.000 | -1.610 | 0.000 |
| Tmem266 | -1.713 | 0.000 | 1.310 | 0.002 |
| Ska1 | 3.983 | 0.000 | -1.908 | 0.004 |
| Myl12a | 2.839 | 0.000 | -1.696 | 0.000 |
| Nt5c1a | -1.151 | 0.000 | 1.024 | 0.010 |
| RGD1561662 | 3.147 | 0.000 | -2.315 | 0.000 |
| Pbk | 4.561 | 0.000 | -1.876 | 0.038 |
| Lrrc32 | 1.920 | 0.000 | -1.331 | 0.006 |
| Kpna2 | 1.944 | 0.000 | -1.108 | 0.007 |
| Neurl3 | 4.702 | 0.000 | -2.853 | 0.004 |
| Arid5a | 1.164 | 0.000 | -1.391 | 0.000 |
| Asxl3 | -1.794 | 0.000 | 1.346 | 0.000 |
| LOC100912849 | 2.454 | 0.000 | -1.789 | 0.006 |
| Apobec1 | 3.231 | 0.000 | -1.568 | 0.033 |
| Nabp1 | 1.530 | 0.000 | -1.182 | 0.002 |
| Ccna2 | 4.009 | 0.000 | -1.688 | 0.043 |
| Il4r | 3.118 | 0.000 | -2.171 | 0.000 |
| Mal | -1.524 | 0.047 | 1.299 | 0.027 |
| Dipk1c | -2.145 | 0.000 | 1.899 | 0.002 |
| Tmem171 | 2.273 | 0.001 | -1.914 | 0.013 |
| Cep72 | 1.875 | 0.000 | -1.321 | 0.016 |
| Bpifb1 | 1.838 | 0.024 | -1.872 | 0.042 |
| Nkx3-1 | -1.723 | 0.000 | 1.529 | 0.020 |
| Tead1 | 1.177 | 0.000 | -1.247 | 0.001 |
| Tpm4 | 2.851 | 0.000 | -1.786 | 0.000 |
| Ces1d | -3.755 | 0.003 | 4.008 | 0.025 |
| Ticrr | 3.457 | 0.000 | -2.232 | 0.007 |
| Abcd2 | -1.540 | 0.000 | 1.408 | 0.001 |
| Cdh17 | 6.344 | 0.000 | -2.993 | 0.029 |
| Plpp5 | 1.048 | 0.000 | -1.042 | 0.000 |
| Lpar6 | 2.058 | 0.000 | -1.629 | 0.000 |
| Sigirr | 1.797 | 0.000 | -1.356 | 0.001 |
| Mall | 2.802 | 0.000 | -1.603 | 0.009 |
| Acap1 | 2.994 | 0.000 | -2.150 | 0.030 |
| Nell1 | -1.667 | 0.000 | 1.098 | 0.016 |
| Slc2a13 | -1.557 | 0.000 | 1.295 | 0.000 |
| Kcng4 | -1.754 | 0.000 | 1.359 | 0.018 |
| Cyp4f18 | 3.499 | 0.000 | -3.414 | 0.002 |
| Nat8f3 | -2.718 | 0.000 | 2.396 | 0.001 |
| Nat8f5 | -2.304 | 0.000 | 2.139 | 0.000 |
| Il21r | 2.636 | 0.000 | -1.818 | 0.021 |
| Trip13 | 2.876 | 0.000 | -1.908 | 0.002 |
| Tm4sf1 | 2.218 | 0.000 | -1.816 | 0.000 |
| Cd2 | 4.375 | 0.000 | -3.318 | 0.004 |
| Niban2 | 2.045 | 0.000 | -1.409 | 0.000 |
| Npsr1 | -2.341 | 0.000 | 2.476 | 0.002 |
| Pif1 | 3.245 | 0.000 | -1.513 | 0.028 |
| Dock8 | 2.248 | 0.000 | -1.482 | 0.006 |
| Tgif1 | 2.692 | 0.000 | -1.586 | 0.001 |
| Esco2 | 3.242 | 0.000 | -1.587 | 0.009 |
| Fkbp10 | 1.799 | 0.000 | -1.082 | 0.006 |
| Slc1a5 | 2.542 | 0.000 | -1.912 | 0.000 |
| Usp29 | -1.489 | 0.000 | 1.039 | 0.000 |
| F13a1 | 2.234 | 0.000 | -2.495 | 0.000 |
| Ccl20 | 6.594 | 0.000 | -6.123 | 0.000 |
| Mafb | 2.699 | 0.000 | -2.194 | 0.000 |
| Zc3h12d | 3.654 | 0.000 | -2.199 | 0.006 |
| Mab21l3 | 3.220 | 0.001 | -3.444 | 0.007 |
| Hecw1 | -1.110 | 0.000 | 1.012 | 0.017 |
| Trpc3 | -1.690 | 0.000 | 1.257 | 0.009 |
| Cd274 | 1.328 | 0.000 | -1.090 | 0.009 |
| Myof | 1.478 | 0.000 | -1.150 | 0.009 |
| Slc17a6 | -1.872 | 0.001 | 1.085 | 0.041 |
| Gtse1 | 2.317 | 0.000 | -1.543 | 0.004 |
| Pdlim1 | 3.020 | 0.000 | -2.139 | 0.000 |
| RGD1562114 | 1.400 | 0.000 | -1.185 | 0.000 |
| Vnn1 | 2.754 | 0.000 | -2.184 | 0.000 |
| Ppp1r3g | -1.074 | 0.002 | 1.069 | 0.006 |
| Casq2 | -1.731 | 0.010 | 1.598 | 0.048 |
| Sox18 | 1.703 | 0.000 | -1.432 | 0.000 |
| Cotl1 | 1.395 | 0.000 | -1.392 | 0.000 |
| Col4a1 | 3.283 | 0.000 | -2.335 | 0.000 |
| Klf4 | 1.077 | 0.000 | -1.054 | 0.001 |
| Il10ra | 1.467 | 0.000 | -1.138 | 0.005 |
| Mcm2 | 2.999 | 0.000 | -1.888 | 0.001 |
| Camk2n1 | -1.403 | 0.000 | 1.419 | 0.003 |
| Cx3cl1 | -1.471 | 0.000 | 1.254 | 0.000 |
| Ptbp3 | 1.586 | 0.000 | -1.466 | 0.001 |
| Frrs1 | 2.680 | 0.000 | -1.865 | 0.000 |
| Gpr4 | 1.474 | 0.000 | -1.393 | 0.002 |
| Vasp | 2.110 | 0.000 | -1.491 | 0.000 |
| Slc12a7 | 1.487 | 0.000 | -1.491 | 0.001 |
| Cep55 | 3.755 | 0.000 | -1.774 | 0.031 |
| Map3k8 | 2.098 | 0.000 | -1.267 | 0.006 |
| Gabrd | -2.328 | 0.000 | 2.255 | 0.000 |
| Tmprss13 | 2.495 | 0.000 | -1.451 | 0.012 |
| Pstpip1 | 2.249 | 0.000 | -1.856 | 0.000 |
| Rnh1 | 1.841 | 0.000 | -1.184 | 0.000 |
| Cd1d1 | 1.721 | 0.000 | -1.246 | 0.000 |
| Nasp | 1.258 | 0.000 | -1.093 | 0.000 |
| Il33 | 1.470 | 0.000 | -1.416 | 0.001 |
| Kctd1 | -1.179 | 0.000 | 1.317 | 0.000 |
| Fxyd2 | 4.024 | 0.000 | -2.553 | 0.000 |
| Myo16 | -1.439 | 0.001 | 1.103 | 0.014 |
| Nop58 | 1.043 | 0.000 | -1.006 | 0.000 |
| Fam110d | 1.850 | 0.000 | -1.034 | 0.001 |
| Ctsc | 3.974 | 0.000 | -2.789 | 0.000 |
| Card19 | 2.062 | 0.000 | -1.515 | 0.001 |
| Pclaf | 4.083 | 0.000 | -2.110 | 0.014 |
| Tnfrsf1b | 3.074 | 0.000 | -2.312 | 0.000 |
| Serpinb1a | 3.723 | 0.000 | -2.350 | 0.001 |
| Ninj1 | 1.826 | 0.000 | -1.374 | 0.000 |
| Hhex | 1.433 | 0.000 | -1.094 | 0.008 |
| Wwtr1 | 1.484 | 0.000 | -1.098 | 0.002 |
| Tln1 | 1.310 | 0.000 | -1.088 | 0.003 |
| Dner | -1.192 | 0.002 | 1.081 | 0.033 |
| Angptl2 | 2.620 | 0.000 | -1.912 | 0.000 |
| Angpt2 | 3.546 | 0.000 | -2.250 | 0.000 |
| Fanca | 2.020 | 0.000 | -1.069 | 0.010 |
| Epb41l3 | -1.351 | 0.000 | 1.177 | 0.000 |
| Tcerg1l | -1.222 | 0.010 | 1.345 | 0.023 |
| Crispld2 | 2.560 | 0.000 | -1.373 | 0.030 |
| Ptgir | 3.190 | 0.000 | -2.095 | 0.000 |
| Loxl2 | 2.234 | 0.000 | -2.166 | 0.000 |
| Cd3eap | 1.149 | 0.000 | -1.090 | 0.000 |
| Pla2g2d | 2.206 | 0.000 | -2.267 | 0.001 |
| Serpinh1 | 1.812 | 0.000 | -1.187 | 0.002 |
| Apobec3 | 3.543 | 0.000 | -2.550 | 0.000 |
| Pla2g2a | 3.096 | 0.011 | -4.596 | 0.002 |
| Neu2 | -1.163 | 0.002 | 1.813 | 0.003 |
| LOC308990 | 2.383 | 0.000 | -2.191 | 0.000 |
| Nkx1-2 | -4.520 | 0.000 | 3.496 | 0.006 |
| Ccdc183 | -1.349 | 0.040 | 1.754 | 0.012 |
| Fcna | 1.574 | 0.000 | -1.302 | 0.005 |
| Pxdc1 | 1.535 | 0.000 | -1.345 | 0.000 |
| Ybx2 | -1.545 | 0.000 | 1.162 | 0.014 |
| Ankle1 | 3.709 | 0.000 | -1.760 | 0.033 |
| Lingo1 | -1.295 | 0.000 | 1.127 | 0.007 |
| Tcirg1 | 1.893 | 0.000 | -1.456 | 0.000 |
| Slc2a4 | -1.494 | 0.000 | 1.726 | 0.001 |
| Ica1l | -1.614 | 0.000 | 1.456 | 0.000 |
| Tacc3 | 3.116 | 0.000 | -1.681 | 0.011 |
| Igsf6 | 4.255 | 0.000 | -3.035 | 0.000 |
| Rhobtb2 | -1.161 | 0.000 | 1.069 | 0.001 |
| Il11 | 6.856 | 0.000 | -3.994 | 0.000 |
| Apobr | 1.744 | 0.000 | -1.216 | 0.003 |
| Irf7 | 1.415 | 0.007 | -1.942 | 0.003 |
| Ptpn2 | 1.358 | 0.000 | -1.092 | 0.000 |
| Anxa1 | 4.061 | 0.000 | -2.457 | 0.000 |
| Gja5 | 4.440 | 0.000 | -3.084 | 0.000 |
| Mmp9 | 2.451 | 0.002 | -1.564 | 0.034 |
| Chrm4 | -1.132 | 0.000 | 1.187 | 0.000 |
| Aldh1a1 | -2.095 | 0.000 | 1.425 | 0.007 |
| Npm3 | 1.402 | 0.000 | -1.362 | 0.000 |
| Htr2b | 6.169 | 0.000 | -3.562 | 0.001 |
| RGD1309651 | -1.818 | 0.000 | 1.468 | 0.001 |
| Cckbr | -1.754 | 0.000 | 1.322 | 0.008 |
| Dennd2d | 3.112 | 0.000 | -2.191 | 0.001 |
| Adam18 | 3.923 | 0.009 | -4.419 | 0.011 |
| Itih3 | -2.324 | 0.000 | 2.455 | 0.000 |
| AABR07012795.1 | 1.417 | 0.000 | -1.024 | 0.000 |
| Gpld1 | -1.878 | 0.000 | 1.526 | 0.000 |
| Unc93b1 | 2.373 | 0.000 | -1.817 | 0.000 |
| Pnlip | -1.939 | 0.000 | 1.428 | 0.008 |
| Ripk1 | 1.475 | 0.000 | -1.147 | 0.001 |
| Klf15 | -1.245 | 0.000 | 1.328 | 0.001 |
| Cd14 | 6.849 | 0.000 | -3.606 | 0.000 |
| Ucp2 | 1.677 | 0.000 | -1.328 | 0.001 |
| Irf8 | 2.441 | 0.000 | -1.385 | 0.012 |
| Cd53 | 3.018 | 0.000 | -2.277 | 0.000 |
| Adam8 | 4.409 | 0.000 | -3.852 | 0.000 |
| Chn1 | -1.566 | 0.000 | 1.339 | 0.001 |
| Serpinb6a | 1.462 | 0.000 | -1.040 | 0.000 |
| Olfm3 | -1.104 | 0.001 | 1.135 | 0.001 |
| Itgal | 4.263 | 0.000 | -2.353 | 0.003 |
| Mcm10 | 4.024 | 0.000 | -2.037 | 0.007 |
| F2rl1 | 2.696 | 0.000 | -2.026 | 0.002 |
| Doc2g | -1.534 | 0.000 | 1.680 | 0.001 |
| Gins4 | 2.051 | 0.000 | -1.230 | 0.003 |
| Fmo5 | 3.358 | 0.000 | -1.978 | 0.017 |
| Slc26a2 | 1.664 | 0.000 | -1.232 | 0.012 |
| Vim | 4.167 | 0.000 | -1.427 | 0.039 |
| Slc12a5 | -1.777 | 0.000 | 1.474 | 0.001 |
| Anxa4 | 1.938 | 0.000 | -1.188 | 0.005 |
| Prkab2 | 1.782 | 0.000 | -1.217 | 0.001 |
| Dynlt1 | 1.094 | 0.000 | -1.079 | 0.000 |
| Mrc1 | 2.216 | 0.000 | -1.891 | 0.002 |
| Hpx | 2.762 | 0.003 | -2.228 | 0.012 |
| Hrh2 | -1.696 | 0.000 | 1.557 | 0.000 |
| Hhip | -1.697 | 0.005 | 1.241 | 0.023 |
| Kcna2 | -1.212 | 0.000 | 1.174 | 0.002 |
| Caly | -1.230 | 0.000 | 1.055 | 0.012 |
| Vwa1 | 2.497 | 0.000 | -1.713 | 0.000 |
| Nt5dc2 | 1.308 | 0.000 | -1.248 | 0.004 |
| RGD1310819 | -1.703 | 0.000 | 1.378 | 0.006 |
| Tubb6 | 4.895 | 0.000 | -2.552 | 0.000 |
| Adam12 | 2.053 | 0.000 | -2.041 | 0.000 |
| Chrm1 | -1.349 | 0.000 | 1.161 | 0.008 |
| Aen | 1.025 | 0.000 | -1.042 | 0.000 |
| Stab1 | 1.169 | 0.005 | -1.211 | 0.013 |
| Agt | -3.159 | 0.000 | 2.184 | 0.000 |
| Cd40 | 2.804 | 0.000 | -1.508 | 0.022 |
| LOC100911186 | 3.962 | 0.002 | -4.345 | 0.001 |
| Pitpnm1 | -1.270 | 0.000 | 1.036 | 0.001 |
| Ctsl | 2.035 | 0.000 | -1.439 | 0.000 |
| Cenph | 3.459 | 0.000 | -1.699 | 0.011 |
| Hbegf | 3.742 | 0.000 | -2.750 | 0.000 |
| Csf1 | 1.265 | 0.000 | -1.018 | 0.039 |
| Nes | 4.173 | 0.000 | -2.457 | 0.000 |
| RGD1305464 | 1.238 | 0.000 | -1.150 | 0.001 |
| Lipg | 2.502 | 0.000 | -1.997 | 0.007 |
| Il15ra | 3.043 | 0.000 | -1.464 | 0.007 |
| Camk2a | -2.303 | 0.000 | 1.775 | 0.003 |
| Clec10a | 2.439 | 0.000 | -1.544 | 0.012 |
| Cd74 | 1.994 | 0.000 | -2.261 | 0.000 |
| Ugt1a6 | 2.476 | 0.000 | -1.715 | 0.000 |
| Clcf1 | 3.052 | 0.000 | -2.203 | 0.000 |
| Pmaip1 | 1.687 | 0.000 | -1.181 | 0.003 |
| Gmnn | 2.522 | 0.000 | -1.571 | 0.000 |
| Jph3 | -1.419 | 0.000 | 1.202 | 0.001 |
| Kcnh4 | -1.202 | 0.000 | 1.199 | 0.015 |
| Bcan | -1.648 | 0.000 | 1.061 | 0.001 |
| Plk1 | 3.633 | 0.000 | -1.754 | 0.021 |
| RGD1559896 | -1.640 | 0.000 | 1.048 | 0.000 |
| Pou4f3 | 5.632 | 0.000 | -2.828 | 0.023 |
| Nppc | -2.433 | 0.000 | 1.961 | 0.001 |
| Myct1 | 2.350 | 0.006 | -1.984 | 0.049 |
| Phf19 | 1.768 | 0.000 | -1.583 | 0.000 |
| Tagap | 2.779 | 0.000 | -1.628 | 0.003 |
| Kif20b | 2.362 | 0.000 | -1.332 | 0.028 |
| Slc28a1 | 1.695 | 0.010 | -1.284 | 0.032 |
| Dlk2 | -1.604 | 0.000 | 1.328 | 0.004 |
| AABR07001382.1 | -1.267 | 0.000 | 1.165 | 0.000 |
| Mob3a | 1.294 | 0.000 | -1.134 | 0.001 |
| Zfp385b | -1.259 | 0.000 | 1.252 | 0.000 |
| Nrg2 | -1.150 | 0.000 | 1.098 | 0.004 |
| Kif2c | 4.055 | 0.000 | -1.952 | 0.046 |
| Ocstamp | 4.186 | 0.000 | -3.498 | 0.001 |
| Scamp2 | 1.538 | 0.000 | -1.129 | 0.001 |
| Ch25h | 3.335 | 0.000 | -2.186 | 0.000 |
| Stom | 2.459 | 0.000 | -1.510 | 0.002 |
| Psd2 | -1.192 | 0.000 | 1.049 | 0.001 |
| Ggta1 | 2.293 | 0.000 | -1.439 | 0.004 |
| Pvr | 2.787 | 0.000 | -2.367 | 0.000 |
| Rhod | 2.060 | 0.000 | -1.809 | 0.019 |
| Fbl | 1.300 | 0.000 | -1.321 | 0.000 |
| Ltbr | 1.715 | 0.000 | -1.213 | 0.001 |
| P2ry6 | 3.192 | 0.000 | -2.005 | 0.000 |
| P2ry2 | 2.097 | 0.001 | -1.935 | 0.001 |
| Nfkb2 | 2.137 | 0.000 | -1.615 | 0.000 |
| Procr | 3.032 | 0.000 | -1.701 | 0.000 |
| Tax1bp3 | 2.416 | 0.000 | -1.415 | 0.000 |
| Ifi30 | 3.141 | 0.000 | -1.932 | 0.000 |
| Egfl7 | 1.487 | 0.000 | -1.427 | 0.000 |
| Hhatl | -1.753 | 0.000 | 1.840 | 0.004 |
| Rhbg | 3.316 | 0.001 | -2.085 | 0.041 |
| Rab3a | -1.354 | 0.000 | 1.186 | 0.001 |
| Kcnn4 | 5.185 | 0.000 | -3.511 | 0.000 |
| Agpat2 | 3.142 | 0.000 | -2.314 | 0.000 |
| Zmynd15 | 2.547 | 0.000 | -1.772 | 0.027 |
| Slc6a9 | -1.298 | 0.000 | 1.111 | 0.000 |
| Trpv1 | -1.600 | 0.000 | 1.233 | 0.002 |
| Stat5a | 2.008 | 0.000 | -1.420 | 0.003 |
| Plekhg6 | 1.456 | 0.000 | -1.038 | 0.000 |
| Smim3 | 1.602 | 0.000 | -1.107 | 0.000 |
| Akap12 | 1.351 | 0.000 | -1.139 | 0.011 |
| Adamts19 | -2.793 | 0.000 | 2.328 | 0.001 |
| Pmf1 | 2.744 | 0.000 | -1.439 | 0.005 |
| Lmna | 1.661 | 0.000 | -1.413 | 0.000 |
| Osbp2 | -1.366 | 0.000 | 1.078 | 0.006 |
| AABR07068316.1 | 2.239 | 0.000 | -1.902 | 0.000 |
| Gdf15 | 3.689 | 0.000 | -3.946 | 0.000 |
| Pycard | 3.522 | 0.000 | -1.769 | 0.012 |
| Pold1 | 2.160 | 0.000 | -1.424 | 0.000 |
| Vwf | 2.458 | 0.000 | -1.102 | 0.015 |
| Itgam | 3.346 | 0.000 | -2.068 | 0.001 |
| Stat3 | 1.990 | 0.000 | -1.021 | 0.004 |
| Gadd45b | 2.440 | 0.000 | -1.557 | 0.000 |
| Hsd17b1 | 2.153 | 0.000 | -1.004 | 0.043 |
| Gmfg | 2.975 | 0.000 | -2.123 | 0.000 |
| Napsa | 4.280 | 0.000 | -2.771 | 0.000 |
| Folr2 | 4.520 | 0.000 | -2.600 | 0.000 |
| Lrrfip1 | 1.299 | 0.000 | -1.207 | 0.000 |
| Rhbdl1 | -1.598 | 0.000 | 1.096 | 0.003 |
| Map3k21 | -1.492 | 0.013 | 1.740 | 0.026 |
| Kcnk1 | -1.187 | 0.000 | 1.150 | 0.002 |
| Fbxo17 | 1.259 | 0.007 | -1.379 | 0.025 |
| Tmem151b | -1.664 | 0.000 | 1.159 | 0.005 |
| Kcnc3 | -1.166 | 0.000 | 1.076 | 0.014 |
| Tcte1 | -1.546 | 0.000 | 1.276 | 0.000 |
| Celsr2 | -1.383 | 0.000 | 1.009 | 0.002 |
| Klhl30 | 2.844 | 0.000 | -1.932 | 0.005 |
| Tgm1 | 5.281 | 0.000 | -3.238 | 0.001 |
| Mvp | 2.366 | 0.000 | -1.452 | 0.000 |
| Ctsd | 1.683 | 0.000 | -1.038 | 0.003 |
| Homer3 | 1.276 | 0.002 | -1.293 | 0.001 |
| RGD1305347 | -1.899 | 0.026 | 1.619 | 0.049 |
| Kif22 | 3.388 | 0.000 | -1.750 | 0.025 |
| Bag3 | 2.128 | 0.000 | -1.096 | 0.002 |
| Lsp1 | 4.973 | 0.000 | -2.409 | 0.001 |
| Rab3il1 | 1.445 | 0.000 | -1.171 | 0.005 |
| Lgals7 | 2.298 | 0.000 | -1.242 | 0.009 |
| Ltb4r | 4.784 | 0.000 | -4.178 | 0.000 |
| Adcy4 | 2.678 | 0.000 | -1.811 | 0.000 |
| Zfr2 | -1.594 | 0.000 | 1.245 | 0.001 |
| Ripk3 | 4.663 | 0.000 | -2.486 | 0.000 |
| Slc25a24 | 2.314 | 0.000 | -1.496 | 0.000 |
| Camk4 | -2.399 | 0.000 | 1.641 | 0.008 |
| Bcl2l12 | 1.962 | 0.000 | -1.122 | 0.006 |
| Tmem145 | -1.506 | 0.000 | 1.563 | 0.004 |
| Map4k1 | 1.815 | 0.000 | -1.172 | 0.003 |
| Fosl1 | 1.574 | 0.000 | -1.529 | 0.000 |
| AABR07005821.1 | 4.680 | 0.001 | -3.028 | 0.030 |
| Col7a1 | 2.835 | 0.000 | -2.097 | 0.000 |
| Gipc3 | 1.579 | 0.001 | -1.427 | 0.012 |
| Tgfb1 | 2.589 | 0.000 | -1.802 | 0.000 |
| S1pr2 | 1.525 | 0.000 | -1.130 | 0.000 |
| Ucn2 | 8.077 | 0.000 | -2.945 | 0.015 |
| Shc1 | 2.390 | 0.000 | -1.733 | 0.000 |
| Icam1 | 3.185 | 0.000 | -2.130 | 0.000 |
| Vat1 | 1.681 | 0.000 | -1.447 | 0.001 |
| Tead2 | 1.580 | 0.000 | -1.340 | 0.010 |
| Cd37 | 1.846 | 0.000 | -1.171 | 0.007 |
| Brca1 | 2.199 | 0.000 | -1.347 | 0.007 |
| Axl | 1.354 | 0.000 | -1.024 | 0.002 |
| Hrc | -1.490 | 0.004 | 1.186 | 0.025 |
| Nme6 | 1.488 | 0.000 | -1.179 | 0.000 |
| Sipa1 | 1.927 | 0.000 | -1.269 | 0.001 |
| Lin7b | -2.071 | 0.000 | 1.658 | 0.001 |
| Il6r | 1.435 | 0.000 | -1.137 | 0.000 |
| Hipk4 | -1.307 | 0.000 | 1.120 | 0.005 |
| LOC100360087 | 1.769 | 0.000 | -1.315 | 0.000 |
| Tyrobp | 3.550 | 0.000 | -2.239 | 0.000 |
| Hcst | 4.790 | 0.000 | -2.711 | 0.000 |
| Pyy | 5.856 | 0.000 | -4.183 | 0.000 |
| Ccnd1 | 2.273 | 0.000 | -1.967 | 0.000 |
| Tuft1 | 1.141 | 0.009 | -1.011 | 0.023 |
| Nradd | 1.943 | 0.000 | -1.121 | 0.003 |
| Hsd17b14 | -1.592 | 0.007 | 1.671 | 0.039 |
| Ms4a7 | 2.376 | 0.001 | -2.682 | 0.001 |
| AABR07006278.1 | 3.690 | 0.004 | -4.189 | 0.004 |
| Ms4a6a | 4.771 | 0.000 | -2.449 | 0.000 |
| Ca11 | -1.874 | 0.000 | 1.810 | 0.000 |
| Dbp | -1.775 | 0.000 | 1.342 | 0.001 |
| Grn | 2.067 | 0.000 | -1.321 | 0.001 |
| Fxyd5 | 3.136 | 0.000 | -1.797 | 0.000 |
| Fxyd7 | -2.283 | 0.000 | 1.527 | 0.007 |
| AABR07006310.1 | 1.888 | 0.000 | -1.464 | 0.002 |
| Pygm | -1.153 | 0.000 | 1.218 | 0.002 |
| Tnfaip8l2 | 1.995 | 0.000 | -1.352 | 0.001 |
| Emp3 | 4.029 | 0.000 | -2.254 | 0.000 |
| Prdx5 | 1.599 | 0.000 | -1.308 | 0.000 |
| Kcnk4 | -1.473 | 0.000 | 1.206 | 0.016 |
| Ppp1r14b | 2.142 | 0.000 | -1.309 | 0.000 |
| Fermt3 | 3.400 | 0.000 | -2.209 | 0.000 |
| Ecm1 | 2.408 | 0.000 | -1.419 | 0.002 |
| Otol1 | 3.218 | 0.026 | -3.712 | 0.022 |
| Fcgr1a | 3.550 | 0.000 | -2.599 | 0.000 |
| Lzts3 | -1.668 | 0.000 | 1.368 | 0.005 |
| Gfra4 | -1.104 | 0.002 | 1.072 | 0.004 |
| Siglec1 | 4.270 | 0.000 | -2.793 | 0.001 |
| Pcna | 1.721 | 0.000 | -1.183 | 0.000 |
| Slc36a3 | 3.957 | 0.003 | -3.491 | 0.011 |
| Lag3 | 3.485 | 0.000 | -2.118 | 0.002 |
| Ccdc69 | 3.212 | 0.000 | -2.096 | 0.008 |
| Fads6 | -1.060 | 0.000 | 1.049 | 0.003 |
| Negr1 | -1.589 | 0.000 | 1.219 | 0.013 |
| Slfn13 | 3.113 | 0.000 | -1.773 | 0.000 |
| AABR07073181.1 | 2.337 | 0.000 | -2.251 | 0.000 |
| Tuba1c | 1.170 | 0.004 | -1.170 | 0.023 |
| Luzp2 | -1.656 | 0.000 | 1.251 | 0.000 |
| Slc25a34 | -1.133 | 0.002 | 1.071 | 0.003 |
| Mis18a | 2.318 | 0.001 | -2.085 | 0.003 |
| Cass4 | 1.230 | 0.002 | -1.686 | 0.002 |
| Slc15a3 | 2.086 | 0.000 | -1.624 | 0.001 |
| Pld6 | -2.159 | 0.003 | 1.860 | 0.022 |
| Mybl1 | 1.680 | 0.000 | -1.374 | 0.000 |
| Kif18b | 3.193 | 0.000 | -2.055 | 0.014 |
| Id1 | 1.844 | 0.000 | -1.287 | 0.000 |
| Zfp217 | 1.639 | 0.000 | -1.298 | 0.006 |
| Fam227a | -1.076 | 0.003 | 1.215 | 0.006 |
| Isg15 | 1.302 | 0.002 | -1.371 | 0.014 |
| Irak2 | 1.334 | 0.000 | -1.017 | 0.002 |
| AABR07018318.1 | -1.972 | 0.000 | 1.640 | 0.003 |
| Lat2 | 2.952 | 0.000 | -1.556 | 0.003 |
| Rhoj | 2.154 | 0.000 | -1.549 | 0.004 |
| Ccdc184 | -2.254 | 0.000 | 1.557 | 0.001 |
| Meioc | -1.945 | 0.026 | 2.076 | 0.022 |
| Aunip | 2.275 | 0.000 | -1.260 | 0.002 |
| LOC103690006 | 3.223 | 0.000 | -2.147 | 0.040 |
| Lacc1 | 2.542 | 0.000 | -1.954 | 0.001 |
| Trim72 | 3.695 | 0.000 | -2.040 | 0.006 |
| Crabp2 | 2.658 | 0.000 | -1.819 | 0.003 |
| Gjb6 | -1.316 | 0.003 | 1.529 | 0.003 |
| Cxcl10 | 2.069 | 0.000 | -2.047 | 0.000 |
| Cenpu | 3.669 | 0.000 | -1.891 | 0.001 |
| AABR07039316.1 | -1.375 | 0.005 | 1.110 | 0.047 |
| Cxcl11 | 3.692 | 0.000 | -2.318 | 0.000 |
| Primpol | 1.993 | 0.000 | -1.290 | 0.002 |
| Opalin | -1.763 | 0.007 | 1.330 | 0.020 |
| Hspb8 | 1.939 | 0.000 | -1.189 | 0.000 |
| Prickle3 | 1.554 | 0.000 | -1.305 | 0.002 |
| LOC102546864 | 2.951 | 0.000 | -1.631 | 0.007 |
| Flrt1 | -1.559 | 0.000 | 1.232 | 0.003 |
| Sgo1 | 3.580 | 0.000 | -1.624 | 0.013 |
| Ddias | 3.335 | 0.000 | -2.044 | 0.001 |
| Lrrc25 | 4.897 | 0.000 | -2.923 | 0.000 |
| LOC100362965 | -1.091 | 0.048 | 1.309 | 0.024 |
| Trerf1 | -1.648 | 0.000 | 1.342 | 0.000 |
| Ccdc73 | -1.315 | 0.023 | 1.251 | 0.033 |
| Clec14a | 1.783 | 0.000 | -1.529 | 0.002 |
| Esyt3 | -1.408 | 0.000 | 1.008 | 0.048 |
| Pmp2 | 3.820 | 0.000 | -2.427 | 0.000 |
| Prrg4 | 3.490 | 0.000 | -2.336 | 0.015 |
| Mast3 | -1.370 | 0.000 | 1.010 | 0.001 |
| Rubcnl | 1.242 | 0.001 | -1.011 | 0.019 |
| Sp100 | 2.163 | 0.000 | -1.443 | 0.007 |
| AABR07064386.1 | -2.052 | 0.018 | 2.086 | 0.041 |
| Sp140 | 2.286 | 0.000 | -1.514 | 0.003 |
| Ifit3 | 2.914 | 0.000 | -2.965 | 0.000 |
| Pdzrn4 | -1.511 | 0.008 | 1.322 | 0.023 |
| Snx22 | -2.034 | 0.000 | 1.384 | 0.042 |
| Trem1 | 8.541 | 0.000 | -4.392 | 0.000 |
| Hjurp | 2.909 | 0.000 | -1.874 | 0.008 |
| Dact2 | -1.068 | 0.034 | 1.258 | 0.043 |
| Slc22a3 | -1.486 | 0.016 | 1.914 | 0.009 |
| LOC681325 | 6.913 | 0.000 | -3.605 | 0.000 |
| Mis18bp1 | 2.340 | 0.000 | -1.457 | 0.019 |
| Agmo | -1.751 | 0.001 | 1.370 | 0.012 |
| Nlrp1a | 1.784 | 0.000 | -1.122 | 0.022 |
| Col11a1 | 1.540 | 0.000 | -1.144 | 0.013 |
| Defb29 | 4.141 | 0.000 | -2.686 | 0.004 |
| Myorg | -1.586 | 0.000 | 1.169 | 0.000 |
| Il20rb | 5.636 | 0.000 | -3.430 | 0.000 |
| S100a10 | 4.453 | 0.000 | -1.866 | 0.004 |
| Adamts9 | 2.987 | 0.000 | -2.926 | 0.000 |
| Cenpm | 1.435 | 0.000 | -1.001 | 0.005 |
| Hfm1 | -1.121 | 0.001 | 1.054 | 0.024 |
| Dpep2 | 3.877 | 0.000 | -2.736 | 0.015 |
| Parp14 | 1.791 | 0.000 | -1.481 | 0.000 |
| Sema3a | -1.451 | 0.002 | 1.036 | 0.028 |
| Ephx4 | -1.037 | 0.007 | 1.276 | 0.007 |
| Dtx3l | 1.395 | 0.000 | -1.298 | 0.000 |
| Apol9a | 1.399 | 0.001 | -1.392 | 0.008 |
| Pik3r5 | 2.732 | 0.000 | -1.609 | 0.003 |
| Btbd8 | -1.189 | 0.000 | 1.172 | 0.001 |
| Parp9 | 1.848 | 0.000 | -1.168 | 0.000 |
| Depp1 | 2.786 | 0.000 | -2.489 | 0.000 |
| Plaat5 | 2.467 | 0.000 | -1.923 | 0.004 |
| Aldh5a1 | -1.454 | 0.000 | 1.219 | 0.000 |
| Tmem106a | 2.945 | 0.000 | -2.230 | 0.000 |
| Gprin3 | -1.981 | 0.000 | 1.555 | 0.011 |
| Ntm | -1.140 | 0.000 | 1.066 | 0.000 |
| Nyap2 | -1.486 | 0.000 | 1.219 | 0.006 |
| Ybx1 | 1.727 | 0.000 | -1.094 | 0.001 |
| Opcml | -1.494 | 0.000 | 1.208 | 0.000 |
| Dnase2 | 2.428 | 0.000 | -1.450 | 0.002 |
| Egfem1 | -2.116 | 0.000 | 1.722 | 0.016 |
| Ggn | 1.768 | 0.000 | -1.374 | 0.018 |
| Tbc1d30 | -1.365 | 0.000 | 1.257 | 0.001 |
| Col4a2 | 2.635 | 0.000 | -2.155 | 0.000 |
| Rab20 | 2.119 | 0.000 | -2.163 | 0.000 |
| Cdc25c | 1.739 | 0.000 | -1.101 | 0.011 |
| Sprr1a | 9.426 | 0.000 | -2.962 | 0.032 |
| Fbxo5 | 2.043 | 0.000 | -1.255 | 0.015 |
| Gldn | 4.101 | 0.000 | -1.887 | 0.033 |
| C6 | 4.329 | 0.000 | -2.019 | 0.027 |
| Prr18 | -1.371 | 0.008 | 1.007 | 0.040 |
| Fcer1g | 2.839 | 0.000 | -1.875 | 0.000 |
| Cenpt | 3.534 | 0.000 | -1.679 | 0.043 |
| Shroom2 | -1.676 | 0.000 | 1.438 | 0.000 |
| Cbarp | -1.562 | 0.000 | 1.043 | 0.004 |
| Sertad1 | 1.586 | 0.000 | -1.204 | 0.000 |
| Fcgr3a | 4.140 | 0.000 | -1.928 | 0.015 |
| Osm | 3.566 | 0.000 | -3.094 | 0.000 |
| Eme1 | 3.693 | 0.000 | -1.832 | 0.012 |
| AABR07032520.1 | -1.525 | 0.000 | 1.117 | 0.003 |
| Peg12 | 3.753 | 0.000 | -3.260 | 0.000 |
| Ppp1r35 | -2.034 | 0.042 | 2.787 | 0.013 |
| Gimap9 | 2.534 | 0.000 | -1.915 | 0.001 |
| AC099444.1 | -1.969 | 0.000 | 1.729 | 0.000 |
| Ckap2 | 3.688 | 0.000 | -2.069 | 0.011 |
| Mfsd4a | -1.261 | 0.000 | 1.339 | 0.001 |
| Jpt2 | 1.438 | 0.000 | -1.335 | 0.001 |
| Erfe | 5.854 | 0.000 | -3.528 | 0.000 |
| Arhgap22 | 1.685 | 0.000 | -1.583 | 0.000 |
| Mnd1 | 2.054 | 0.000 | -1.304 | 0.006 |
| Cd93 | 2.537 | 0.000 | -2.183 | 0.000 |
| Fga | 4.944 | 0.000 | -3.863 | 0.015 |
| Pla2g4e | -1.307 | 0.000 | 1.128 | 0.001 |
| Ccdc88b | 1.768 | 0.000 | -1.317 | 0.006 |
| Atp8b1 | 3.881 | 0.000 | -2.988 | 0.001 |
| Mgat5b | -1.652 | 0.000 | 1.316 | 0.001 |
| Stat6 | 1.644 | 0.000 | -1.309 | 0.001 |
| Gpr183 | 2.722 | 0.000 | -1.534 | 0.002 |
| Ikbke | 1.773 | 0.000 | -1.290 | 0.013 |
| Vwa3a | -2.810 | 0.000 | 2.036 | 0.005 |
| Exph5 | -1.282 | 0.002 | 1.297 | 0.019 |
| Ltk | -2.207 | 0.000 | 1.718 | 0.002 |
| Icam2 | 1.615 | 0.000 | -1.409 | 0.003 |
| Gas2l3 | 2.921 | 0.000 | -2.286 | 0.002 |
| Cdca2 | 3.626 | 0.000 | -1.977 | 0.020 |
| Spry1 | 1.455 | 0.000 | -1.260 | 0.000 |
| Mettl11b | -1.242 | 0.049 | 2.084 | 0.000 |
| Tmem252 | 3.659 | 0.000 | -2.067 | 0.001 |
| Jph4 | -1.572 | 0.000 | 1.050 | 0.019 |
| Cdk4 | 1.348 | 0.000 | -1.101 | 0.000 |
| Adgrf2 | 3.306 | 0.000 | -2.661 | 0.002 |
| Lrat | 2.278 | 0.003 | -3.183 | 0.002 |
| Arhgap20 | -1.512 | 0.000 | 1.194 | 0.000 |
| Gpr17 | -2.359 | 0.000 | 1.234 | 0.001 |
| Myh6 | -1.677 | 0.000 | 1.244 | 0.020 |
| AC128848.1 | 3.617 | 0.000 | -1.244 | 0.048 |
| LOC691418 | 3.566 | 0.000 | -2.343 | 0.000 |
| AABR07045485.1 | 1.801 | 0.000 | -1.509 | 0.007 |
| Pdyn | -1.299 | 0.000 | 1.627 | 0.009 |
| Paqr6 | -1.734 | 0.000 | 1.488 | 0.001 |
| Igfn1 | -1.916 | 0.000 | 1.776 | 0.044 |
| Ckap2l | 3.467 | 0.000 | -1.922 | 0.012 |
| Pax8 | 3.200 | 0.003 | -3.019 | 0.010 |
| Hk3 | 5.260 | 0.000 | -3.613 | 0.002 |
| Jun | 1.242 | 0.000 | -1.111 | 0.000 |
| Lrrn4cl | 1.675 | 0.000 | -1.123 | 0.027 |
| Clec5a | 4.293 | 0.000 | -3.114 | 0.000 |
| Taf7l | -1.736 | 0.022 | 1.986 | 0.011 |
| Krt17 | 5.227 | 0.000 | -2.630 | 0.017 |
| Ms4a12 | 3.654 | 0.000 | -1.695 | 0.039 |
| Cdh12 | -1.578 | 0.000 | 1.691 | 0.001 |
| Col14a1 | -3.157 | 0.000 | 2.421 | 0.002 |
| Lrrtm3 | -1.345 | 0.000 | 1.184 | 0.000 |
| Dscc1 | 3.494 | 0.000 | -1.852 | 0.001 |
| Ifi27l2b | 3.473 | 0.000 | -1.606 | 0.002 |
| Tnfsf18 | 3.850 | 0.001 | -3.383 | 0.005 |
| Glipr1 | 2.203 | 0.000 | -1.801 | 0.000 |
| Cxcl16 | 3.757 | 0.000 | -2.716 | 0.000 |
| Hcar2 | 2.495 | 0.000 | -1.529 | 0.012 |
| Arhgap42 | 1.241 | 0.000 | -1.035 | 0.004 |
| Lyve1 | 2.134 | 0.000 | -1.354 | 0.044 |
| RGD1565367 | -1.779 | 0.016 | 1.735 | 0.019 |
| Tril | -1.778 | 0.000 | 1.243 | 0.000 |
| Igtp | 1.297 | 0.000 | -1.049 | 0.000 |
| Rgs16 | 1.644 | 0.000 | -2.044 | 0.000 |
| Adm | 2.963 | 0.000 | -1.176 | 0.029 |
| Alox12 | 1.391 | 0.000 | -1.312 | 0.001 |
| Mboat1 | 3.833 | 0.000 | -2.563 | 0.000 |
| Zfp367 | 1.580 | 0.000 | -1.080 | 0.000 |
| Ephx3 | 2.321 | 0.001 | -1.666 | 0.014 |
| Tenm2 | -1.636 | 0.000 | 1.273 | 0.041 |
| Clec4b2 | 4.865 | 0.020 | -4.396 | 0.033 |
| Cndp1 | 1.563 | 0.000 | -1.292 | 0.000 |
| Slc38a5 | 1.240 | 0.002 | -1.469 | 0.012 |
| Trpm3 | -1.666 | 0.000 | 1.335 | 0.001 |
| Tsku | 2.563 | 0.000 | -1.860 | 0.001 |
| Cdc6 | 4.913 | 0.000 | -2.527 | 0.001 |
| Lilrb4 | 7.740 | 0.000 | -3.850 | 0.010 |
| AABR07041232.1 | -1.682 | 0.001 | 1.873 | 0.012 |
| Fcar | 5.352 | 0.002 | -4.076 | 0.036 |
| Tmem26 | 2.515 | 0.000 | -2.214 | 0.001 |
| Rapgefl1 | -1.236 | 0.000 | 1.058 | 0.003 |
| Iqgap3 | 2.976 | 0.000 | -1.566 | 0.013 |
| Fam83g | 5.172 | 0.000 | -2.814 | 0.004 |
| Plppr3 | -2.000 | 0.000 | 1.533 | 0.003 |
| Crip1 | 2.242 | 0.000 | -1.188 | 0.011 |
| Dscam | -1.449 | 0.000 | 1.050 | 0.003 |
| Pf4 | 2.466 | 0.000 | -1.839 | 0.009 |
| AABR07021402.1 | 3.971 | 0.000 | -2.740 | 0.000 |
| Adamts7 | 3.520 | 0.000 | -3.017 | 0.000 |
| Cxcl3 | 8.061 | 0.000 | -7.116 | 0.000 |
| Tmem35b | 1.963 | 0.000 | -1.065 | 0.047 |
| Tmem255a | -1.356 | 0.000 | 1.102 | 0.000 |
| AABR07011698.1 | -1.452 | 0.027 | 1.839 | 0.005 |
| Mcemp1 | 7.677 | 0.000 | -4.034 | 0.012 |
| Ppp1r1b | -1.562 | 0.000 | 2.525 | 0.001 |
| Cdc20 | 3.691 | 0.000 | -1.873 | 0.010 |
| AC141959.1 | 2.206 | 0.000 | -1.490 | 0.012 |
| Arhgap27 | 1.188 | 0.001 | -1.003 | 0.021 |
| Ksr2 | -1.760 | 0.000 | 1.097 | 0.019 |
| Bcl2l14 | 2.644 | 0.007 | -2.484 | 0.012 |
| Il10rb | 2.059 | 0.000 | -1.301 | 0.001 |
| Siva1 | 1.462 | 0.000 | -1.115 | 0.000 |
| Tox3 | -1.734 | 0.001 | 1.683 | 0.011 |
| Slc28a2 | 3.240 | 0.000 | -3.037 | 0.000 |
| Acvrl1 | 1.132 | 0.000 | -1.003 | 0.007 |
| Lcn12 | -1.438 | 0.008 | 1.970 | 0.000 |
| Gbp4 | 1.330 | 0.012 | -1.576 | 0.002 |
| Gsap | 3.764 | 0.000 | -1.988 | 0.001 |
| Trim80 | 3.261 | 0.016 | -3.278 | 0.025 |
| Slc9a5 | -1.600 | 0.000 | 1.114 | 0.004 |
| Rai14 | 3.005 | 0.000 | -1.910 | 0.001 |
| Rtp4 | 1.012 | 0.033 | -1.251 | 0.022 |
| Dab2 | 2.026 | 0.000 | -1.960 | 0.000 |
| LOC102555453 | 1.241 | 0.000 | -1.302 | 0.001 |
| Ttk | 3.240 | 0.000 | -1.734 | 0.007 |
| LOC102555453 | 1.192 | 0.000 | -1.679 | 0.001 |
| LOC685067 | 2.144 | 0.000 | -1.594 | 0.003 |
| Vcan | 2.075 | 0.000 | -1.872 | 0.000 |
| Plekho2 | 1.010 | 0.000 | -1.034 | 0.003 |
| Pitpnm2 | -1.053 | 0.000 | 1.089 | 0.001 |
| AABR07053166.1 | 1.369 | 0.012 | -1.326 | 0.035 |
| Prrt2 | -1.046 | 0.000 | 1.253 | 0.002 |
| Plxnb1 | -1.935 | 0.000 | 1.177 | 0.002 |
| Cbs | -1.970 | 0.000 | 1.627 | 0.000 |
| Eef1akmt4 | 2.379 | 0.000 | -1.700 | 0.000 |
| Cish | 2.129 | 0.000 | -1.356 | 0.004 |
| LOC304725 | -2.665 | 0.001 | 2.393 | 0.004 |
| AABR07030544.1 | 2.779 | 0.000 | -2.419 | 0.000 |
| Nkain2 | -1.458 | 0.000 | 1.083 | 0.000 |
| Wdfy4 | 3.038 | 0.000 | -1.661 | 0.008 |
| Clic1 | 4.302 | 0.000 | -2.384 | 0.000 |
| Slc35g1 | 1.057 | 0.046 | -1.596 | 0.022 |
| Ccl12 | 6.641 | 0.000 | -4.511 | 0.000 |
| Lrrc4c | -1.225 | 0.000 | 1.025 | 0.001 |
| Gucy1a2 | -1.530 | 0.000 | 1.214 | 0.002 |
| Spock3 | -1.525 | 0.000 | 1.248 | 0.002 |
| RGD1565462 | 5.457 | 0.000 | -4.512 | 0.002 |
| Clec4a2 | 6.328 | 0.000 | -5.071 | 0.000 |
| Ccl6 | 4.363 | 0.000 | -2.939 | 0.001 |
| Sox11 | 1.394 | 0.000 | -1.429 | 0.000 |
| LOC100359600 | 1.655 | 0.000 | -1.682 | 0.000 |
| Msn | 3.643 | 0.000 | -1.985 | 0.000 |
| Plod2 | 1.540 | 0.000 | -1.252 | 0.010 |
| Mmp12 | 5.979 | 0.000 | -4.345 | 0.001 |
| AABR07051892.1 | -1.610 | 0.000 | 1.059 | 0.034 |
| Atp2b2 | -1.434 | 0.000 | 1.280 | 0.001 |
| Rimbp3 | -1.391 | 0.000 | 1.040 | 0.002 |
| Gzmm | -2.563 | 0.000 | 1.815 | 0.010 |
| Olr34 | -1.878 | 0.026 | 1.934 | 0.035 |
| Slc25a45 | 1.796 | 0.000 | -1.124 | 0.020 |
| Ms4a6bl | 2.966 | 0.000 | -1.993 | 0.002 |
| RT1-A2 | 2.197 | 0.000 | -1.395 | 0.015 |
| Cfh | 1.809 | 0.000 | -1.233 | 0.003 |
| Csmd1 | -1.509 | 0.000 | 1.164 | 0.004 |
| C4b | 2.216 | 0.000 | -1.536 | 0.001 |
| Dgkb | -1.765 | 0.000 | 1.490 | 0.008 |
| Sytl2 | 3.781 | 0.000 | -2.602 | 0.018 |
| Htr2c | -2.252 | 0.000 | 1.753 | 0.032 |
| Lexm | 3.706 | 0.000 | -2.791 | 0.000 |
| Rtn4r | -1.168 | 0.000 | 1.022 | 0.011 |
| Samsn1 | 3.928 | 0.000 | -3.235 | 0.000 |
| Was | 2.416 | 0.000 | -1.638 | 0.001 |
| Nfkbiz | 2.666 | 0.000 | -1.728 | 0.000 |
| AABR07054319.1 | 1.977 | 0.000 | -1.298 | 0.004 |
| Trappc3l | -1.350 | 0.041 | 1.449 | 0.026 |
| Zfp804b | -2.687 | 0.000 | 2.315 | 0.007 |
| Tnfrsf1a | 2.547 | 0.000 | -1.468 | 0.001 |
| Hyal2 | 1.622 | 0.000 | -1.187 | 0.001 |
| Eif1a | 1.261 | 0.000 | -1.031 | 0.000 |
| Cdca8 | 2.908 | 0.000 | -1.675 | 0.010 |
| Ftl1 | 1.973 | 0.000 | -1.366 | 0.001 |
| LOC100362384 | 1.680 | 0.000 | -1.321 | 0.000 |
| Zfp648 | -1.779 | 0.002 | 1.571 | 0.022 |
| Zfp455 | 1.482 | 0.000 | -1.081 | 0.005 |
| Atp8b4 | 7.782 | 0.000 | -3.839 | 0.005 |
| Sh2d1b | 1.744 | 0.001 | -1.706 | 0.012 |
| Gls2 | -1.380 | 0.000 | 1.321 | 0.002 |
| AABR07026539.1 | -1.464 | 0.004 | 1.081 | 0.047 |
| Gbp2 | 2.152 | 0.000 | -1.644 | 0.001 |
| Nkain4 | -2.046 | 0.000 | 1.568 | 0.003 |
| Timeless | 2.355 | 0.000 | -1.523 | 0.002 |
| Ttll7 | -1.456 | 0.000 | 1.093 | 0.003 |
| Hapln1 | -2.247 | 0.000 | 1.733 | 0.000 |
| Rnase2 | 4.930 | 0.000 | -3.530 | 0.024 |
| Cenpa | 4.284 | 0.000 | -1.808 | 0.022 |
| Cntnap5b | -1.361 | 0.000 | 1.322 | 0.002 |
| Cyp2d4 | -1.452 | 0.000 | 1.227 | 0.000 |
| Diras2 | -1.896 | 0.000 | 1.330 | 0.004 |
| LOC103690996 | 1.408 | 0.000 | -1.359 | 0.001 |
| Kcnip4 | -1.350 | 0.000 | 1.137 | 0.001 |
| RGD1559575 | 2.520 | 0.000 | -2.229 | 0.000 |
| Tmod3 | 1.761 | 0.000 | -1.218 | 0.001 |
| Recql4 | 2.290 | 0.000 | -1.380 | 0.002 |
| Acot5 | -2.805 | 0.003 | 2.559 | 0.005 |
| Cntn6 | -1.569 | 0.000 | 1.554 | 0.003 |
| RT1-T24-1 | 2.044 | 0.000 | -2.295 | 0.002 |
| Mmp3 | 2.587 | 0.045 | -2.999 | 0.018 |
| Ttyh1 | -1.499 | 0.000 | 1.354 | 0.000 |
| F7 | 5.869 | 0.000 | -5.885 | 0.000 |
| Bub1 | 3.515 | 0.000 | -1.864 | 0.012 |
| Dysf | 2.467 | 0.000 | -1.773 | 0.003 |
| RT1-Da | 2.483 | 0.000 | -2.429 | 0.000 |
| Cyp4f4 | -2.789 | 0.000 | 2.737 | 0.000 |
| Rufy4 | 5.046 | 0.000 | -3.686 | 0.001 |
| Dclk3 | -1.784 | 0.000 | 1.963 | 0.000 |
| Ltbp1 | 1.656 | 0.000 | -1.264 | 0.010 |
| Mef2c | -1.127 | 0.000 | 1.257 | 0.000 |
| Sfn | 3.178 | 0.000 | -2.326 | 0.000 |
| Fam71e2 | 4.029 | 0.014 | -3.565 | 0.030 |
| Osmr | 4.054 | 0.000 | -1.945 | 0.001 |
| RT1-Db1 | 2.041 | 0.001 | -2.311 | 0.004 |
| Esam | 1.754 | 0.000 | -1.240 | 0.002 |
| Ccrl2 | 1.769 | 0.000 | -1.679 | 0.000 |
| Relb | 1.836 | 0.000 | -1.665 | 0.000 |
| LOC691141 | 4.237 | 0.000 | -2.682 | 0.000 |
| Hmgb2l1 | 2.477 | 0.001 | -2.624 | 0.000 |
| Cenpi | 2.766 | 0.000 | -1.372 | 0.007 |
| Csrnp1 | 2.090 | 0.000 | -1.448 | 0.002 |
| Casp12 | 2.086 | 0.001 | -1.860 | 0.007 |
| Igdcc4 | -1.709 | 0.000 | 1.024 | 0.000 |
| LOC100360791 | 1.777 | 0.001 | -1.593 | 0.005 |
| Pappa1 | 1.342 | 0.012 | -1.479 | 0.010 |
| Cfd | 1.533 | 0.000 | -1.329 | 0.008 |
| Arhgap8 | 2.768 | 0.000 | -2.676 | 0.000 |
| Sgk2 | -1.845 | 0.011 | 1.509 | 0.015 |
| Emid1 | -1.244 | 0.000 | 1.044 | 0.012 |
| Cd276 | 1.720 | 0.000 | -1.374 | 0.001 |
| Krt26 | -1.270 | 0.000 | 1.404 | 0.001 |
| Mpp3 | -1.194 | 0.000 | 1.120 | 0.007 |
| Kntc1 | 2.855 | 0.000 | -1.467 | 0.024 |
| Naip2 | 2.969 | 0.000 | -2.027 | 0.001 |
| Rnf207 | -1.482 | 0.000 | 1.075 | 0.037 |
| Ankrd34a | -1.388 | 0.000 | 1.252 | 0.000 |
| Rasgrp4 | 2.441 | 0.000 | -1.806 | 0.007 |
| Sp110 | 2.179 | 0.000 | -1.335 | 0.000 |
| LOC100359668 | 1.576 | 0.007 | -1.177 | 0.027 |
| Ajm1 | -1.337 | 0.000 | 1.049 | 0.005 |
| Fcho1 | -1.609 | 0.000 | 1.271 | 0.001 |
| Gpt | -1.587 | 0.000 | 1.307 | 0.003 |
| Ifnlr1 | 2.092 | 0.000 | -1.555 | 0.001 |
| Plk5 | -2.447 | 0.000 | 2.224 | 0.003 |
| C2cd4a | 1.154 | 0.005 | -1.035 | 0.014 |
| Mir124-2 | -3.084 | 0.000 | 2.873 | 0.010 |
| Mir223 | 3.803 | 0.005 | -2.858 | 0.036 |
| Mir138-2 | -2.603 | 0.000 | 2.616 | 0.003 |
| AC128792.1 | 1.551 | 0.000 | -1.305 | 0.005 |
| AABR07008505.1 | 2.206 | 0.000 | -1.330 | 0.021 |
| Ifit1bl | 2.363 | 0.001 | -2.547 | 0.001 |
| Sectm1b | 6.854 | 0.000 | -3.602 | 0.005 |
| Slc16a3 | 3.347 | 0.000 | -2.325 | 0.000 |
| Notum | -1.879 | 0.000 | 2.120 | 0.000 |
| P4hb | 1.657 | 0.000 | -1.233 | 0.000 |
| Gcgr | 3.148 | 0.000 | -2.188 | 0.008 |
| Itgax | 6.102 | 0.000 | -3.135 | 0.000 |
| Spn | 2.042 | 0.000 | -2.183 | 0.000 |
| Cd300e | 2.911 | 0.001 | -2.740 | 0.002 |
| Cd300lb | 4.260 | 0.000 | -3.546 | 0.000 |
| Tmc5 | -2.044 | 0.000 | 1.836 | 0.011 |
| Itpripl2 | 2.197 | 0.000 | -1.782 | 0.001 |
| Pde1b | -1.535 | 0.000 | 1.671 | 0.000 |
| Nckap1l | 1.480 | 0.000 | -1.068 | 0.008 |
| Gpr84 | 1.953 | 0.000 | -1.117 | 0.024 |
| Nfe2 | 2.940 | 0.000 | -2.913 | 0.000 |
| Arl5c | 1.545 | 0.000 | -1.143 | 0.007 |
| Zgrf1 | 1.571 | 0.001 | -1.283 | 0.007 |
| Xirp1 | 3.245 | 0.000 | -2.548 | 0.001 |
| Slfn2 | 2.472 | 0.000 | -1.988 | 0.000 |
| Shc4 | 1.901 | 0.000 | -1.369 | 0.000 |
| Usp18 | 1.127 | 0.022 | -1.513 | 0.006 |
| Tlcd2 | 1.994 | 0.000 | -1.506 | 0.004 |
| RGD1309036 | -1.067 | 0.000 | 1.228 | 0.000 |
| Rad51 | 3.627 | 0.000 | -2.108 | 0.003 |
| Cd33 | 1.895 | 0.000 | -1.374 | 0.005 |
| Siglec10 | 4.322 | 0.000 | -2.486 | 0.009 |
| Epha10 | -1.498 | 0.000 | 1.016 | 0.012 |
| Scimp | 6.742 | 0.000 | -4.202 | 0.000 |
| Exo5 | 1.429 | 0.000 | -1.179 | 0.000 |
| Tmem196 | -1.294 | 0.000 | 1.507 | 0.001 |
| Pole | 2.610 | 0.000 | -1.442 | 0.005 |
| Chek2 | 2.218 | 0.000 | -1.177 | 0.019 |
| Cd68 | 4.828 | 0.000 | -3.056 | 0.000 |
| Mob3c | 1.335 | 0.000 | -1.092 | 0.007 |
| Calr4 | -3.602 | 0.000 | 2.270 | 0.003 |
| LOC299277 | 4.551 | 0.003 | -4.087 | 0.016 |
| Lims1 | 1.178 | 0.000 | -1.141 | 0.000 |
| Cdk5r2 | -1.327 | 0.000 | 1.111 | 0.004 |
| P2ry10 | 2.360 | 0.009 | -2.236 | 0.019 |
| Spidr | 1.192 | 0.000 | -1.094 | 0.000 |
| Sfxn5 | -2.009 | 0.000 | 1.281 | 0.000 |
| Plaur | 4.580 | 0.000 | -2.837 | 0.000 |
| Aifm3 | -2.151 | 0.000 | 1.624 | 0.000 |
| Kif4a | 2.747 | 0.000 | -1.520 | 0.030 |
| Vsig4 | 7.835 | 0.000 | -5.460 | 0.000 |
| AC120448.1 | 4.885 | 0.000 | -3.276 | 0.013 |
| Slamf6 | 4.339 | 0.000 | -2.954 | 0.001 |
| Akr1c19 | -2.142 | 0.000 | 1.433 | 0.006 |
| AABR07015812.1 | -1.403 | 0.000 | 1.056 | 0.007 |
| AABR07018323.1 | 2.126 | 0.000 | -2.627 | 0.000 |
| Gmnc | -1.509 | 0.006 | 1.538 | 0.022 |
| Lrrc15 | 4.112 | 0.000 | -2.315 | 0.003 |
| Tlr1 | 4.032 | 0.000 | -2.090 | 0.002 |
| Bco2 | 2.383 | 0.000 | -1.686 | 0.001 |
| Cd86 | 3.122 | 0.000 | -2.195 | 0.000 |
| Btn2a2 | 1.333 | 0.006 | -1.103 | 0.016 |
| Hcls1 | 3.519 | 0.000 | -2.068 | 0.001 |
| Dram1 | 2.266 | 0.000 | -1.449 | 0.000 |
| MGC105567 | 1.322 | 0.002 | -1.909 | 0.000 |
| Galntl6 | -1.779 | 0.000 | 1.383 | 0.005 |
| AABR07051878.1 | -1.128 | 0.000 | 1.082 | 0.000 |
| Cip2a | 3.161 | 0.000 | -1.495 | 0.024 |
| Yjefn3 | -2.181 | 0.000 | 1.315 | 0.045 |
| LOC108348074 | 2.793 | 0.000 | -2.723 | 0.000 |
| Pram1 | 2.723 | 0.000 | -1.955 | 0.003 |
| Cfap157 | 1.192 | 0.000 | -1.239 | 0.000 |
| Slc37a2 | 2.721 | 0.000 | -2.159 | 0.000 |
| Pate4 | 5.393 | 0.000 | -4.445 | 0.002 |
| Tmem229a | -1.385 | 0.000 | 1.063 | 0.000 |
| Plp2 | 4.952 | 0.000 | -2.345 | 0.001 |
| Ecscr | 3.667 | 0.000 | -2.844 | 0.000 |
| Col8a1 | 1.406 | 0.045 | -1.952 | 0.005 |
| RT1-CE4 | 1.732 | 0.000 | -1.375 | 0.002 |
| Lrrc73 | -1.389 | 0.000 | 1.143 | 0.002 |
| Adgrg5 | 3.975 | 0.000 | -2.070 | 0.004 |
| AABR07020786.1 | -1.175 | 0.023 | 1.193 | 0.011 |
| Rad54b | 2.361 | 0.000 | -1.927 | 0.000 |
| Dsc2 | 2.273 | 0.000 | -1.904 | 0.026 |
| Ctla2a | 1.660 | 0.000 | -1.234 | 0.004 |
| RGD1565355 | 4.148 | 0.000 | -2.983 | 0.000 |
| Cyp1b1 | 3.017 | 0.000 | -2.474 | 0.001 |
| Mir675 | 7.933 | 0.000 | -2.699 | 0.000 |
| Mir770 | -1.090 | 0.000 | 1.268 | 0.002 |
| Mir760 | -1.726 | 0.000 | 1.566 | 0.017 |
| Il31ra | 5.320 | 0.000 | -3.195 | 0.017 |
| Sting1 | 1.143 | 0.000 | -1.064 | 0.010 |
| Clec4a1 | 3.824 | 0.000 | -2.628 | 0.000 |
| Mroh2a | 1.215 | 0.001 | -1.535 | 0.000 |
| Efcab2 | -1.267 | 0.003 | 1.246 | 0.003 |
| ENSRNOG00000042229 | 1.519 | 0.001 | -1.207 | 0.013 |
| RGD1307182 | 4.516 | 0.000 | -3.887 | 0.001 |
| Nsl1 | 2.172 | 0.000 | -1.321 | 0.007 |
| AABR07052588.1 | -1.699 | 0.000 | 1.573 | 0.001 |
| Mlkl | 2.287 | 0.000 | -2.506 | 0.000 |
| Tgif2 | 1.146 | 0.001 | -1.126 | 0.018 |
| Ankrd63 | -2.993 | 0.000 | 2.638 | 0.001 |
| Hmga2 | 6.392 | 0.000 | -2.809 | 0.023 |
| Adam22 | -1.342 | 0.000 | 1.185 | 0.000 |
| Smim17 | -1.605 | 0.000 | 1.302 | 0.001 |
| Milr1 | 3.519 | 0.000 | -2.203 | 0.035 |
| Cks1b | 1.559 | 0.000 | -1.247 | 0.000 |
| AABR07072264.1 | -2.189 | 0.000 | 1.668 | 0.043 |
| Slc25a18 | -2.872 | 0.000 | 1.810 | 0.000 |
| Fgf13 | -1.684 | 0.000 | 1.148 | 0.006 |
| Apol3 | 1.057 | 0.001 | -1.148 | 0.006 |
| Cd300le | 5.528 | 0.000 | -4.342 | 0.000 |
| Junb | 1.237 | 0.000 | -1.134 | 0.000 |
| Cnn2 | 3.579 | 0.000 | -1.820 | 0.003 |
| Ddx21 | 1.027 | 0.000 | -1.128 | 0.000 |
| AABR07059308.1 | 5.541 | 0.000 | -3.811 | 0.035 |
| Fbn2 | 1.300 | 0.040 | -1.894 | 0.012 |
| Pde4c | 5.014 | 0.000 | -2.884 | 0.037 |
| Birc7 | -1.408 | 0.014 | 1.397 | 0.020 |
| Cap2 | -1.307 | 0.000 | 1.034 | 0.001 |
| Nkpd1 | -1.718 | 0.000 | 1.187 | 0.023 |
| Bcl3 | 4.967 | 0.000 | -2.278 | 0.001 |
| AC123144.1 | -1.780 | 0.000 | 1.432 | 0.024 |
| Spp1 | 10.690 | 0.000 | -3.593 | 0.007 |
| Tnfrsf26 | 4.084 | 0.000 | -2.492 | 0.001 |
| Cyp4x1 | -1.717 | 0.000 | 1.286 | 0.007 |
| Tnfsf9 | 1.922 | 0.000 | -1.136 | 0.033 |
| AC139391.1 | -1.438 | 0.000 | 1.050 | 0.010 |
| LOC102549173 | 3.006 | 0.000 | -1.517 | 0.049 |
| LOC108348108 | 4.041 | 0.001 | -2.742 | 0.027 |
| Etnppl | -1.630 | 0.000 | 2.005 | 0.000 |
| Thbs1 | 4.210 | 0.000 | -3.984 | 0.000 |
| Ephb4 | 1.245 | 0.000 | -1.228 | 0.003 |
| Tlr8 | 4.379 | 0.000 | -3.157 | 0.000 |
| Chst14 | 1.523 | 0.000 | -1.198 | 0.001 |
| Tnfaip8l1 | 2.271 | 0.000 | -1.945 | 0.001 |
| Haus6 | 1.460 | 0.000 | -1.146 | 0.004 |
| Itprip | 2.813 | 0.001 | -1.723 | 0.019 |
| AABR07030793.1 | 6.879 | 0.000 | -6.897 | 0.000 |
| RGD1561143 | 3.806 | 0.026 | -3.818 | 0.038 |
| Cgas | 2.861 | 0.000 | -1.966 | 0.005 |
| Icos | 2.511 | 0.001 | -1.812 | 0.009 |
| Metrnl | 2.374 | 0.000 | -2.051 | 0.000 |
| Cyp27b1 | 4.756 | 0.000 | -3.044 | 0.005 |
| Cd300c2 | 8.379 | 0.000 | -6.213 | 0.000 |
| B3gnt5 | 2.665 | 0.000 | -2.246 | 0.005 |
| Acp5 | 1.940 | 0.000 | -1.049 | 0.000 |
| Rp1l1 | 2.290 | 0.000 | -1.859 | 0.001 |
| Rbpj | 1.445 | 0.000 | -1.142 | 0.003 |
| Npy | 2.454 | 0.000 | -1.237 | 0.031 |
| Fcgr2b | 4.627 | 0.000 | -2.516 | 0.000 |
| Rbm24 | -1.632 | 0.000 | 1.201 | 0.000 |
| Tub | -1.487 | 0.000 | 1.033 | 0.008 |
| Cited4 | -1.328 | 0.001 | 1.605 | 0.002 |
| Cdca5 | 3.768 | 0.000 | -1.846 | 0.021 |
| Cyp3a9 | -1.487 | 0.007 | 1.330 | 0.022 |
| Fcgr2a | 3.427 | 0.000 | -2.600 | 0.000 |
| Slpi | 9.298 | 0.000 | -4.548 | 0.002 |
| Abcc2 | -1.247 | 0.000 | 1.319 | 0.002 |
| Kdr | 1.351 | 0.001 | -1.568 | 0.001 |
| PCOLCE2 | 2.864 | 0.000 | -2.191 | 0.000 |
| Tmem121b | -1.685 | 0.000 | 1.244 | 0.008 |
| B3gat2 | -1.602 | 0.000 | 1.019 | 0.022 |
| Chrm2 | -1.185 | 0.014 | 1.291 | 0.009 |
| Aldh1l1 | -1.202 | 0.000 | 1.094 | 0.002 |
| LOC681341 | 4.396 | 0.000 | -3.448 | 0.008 |
| Hells | 3.157 | 0.000 | -2.386 | 0.017 |
| Spsb2 | 2.913 | 0.002 | -1.875 | 0.050 |
| Zfp551 | -1.094 | 0.000 | 1.047 | 0.003 |
| Prss55 | -2.215 | 0.000 | 1.573 | 0.010 |
| Bdkrb2 | 2.455 | 0.000 | -2.434 | 0.000 |
| Tk1 | 2.857 | 0.000 | -1.553 | 0.013 |
| AABR07006269.1 | 5.318 | 0.000 | -2.830 | 0.000 |
| Kcnk13 | 1.520 | 0.000 | -1.220 | 0.002 |
| Card14 | 1.405 | 0.000 | -1.055 | 0.026 |
| Lmo3 | -1.503 | 0.000 | 1.769 | 0.001 |
| Wasf1 | -1.368 | 0.000 | 1.092 | 0.003 |
| AABR07066700.1 | -1.202 | 0.007 | 1.600 | 0.005 |
| Bcl2a1 | 3.794 | 0.000 | -2.814 | 0.000 |
| Il2ra | 1.019 | 0.032 | -1.394 | 0.011 |
| Crybb3 | 3.522 | 0.022 | -4.921 | 0.009 |
| Dcx | -1.387 | 0.000 | 1.004 | 0.010 |
| Tmem37 | 2.348 | 0.000 | -1.646 | 0.004 |
| Tnfsf14 | 5.471 | 0.000 | -2.852 | 0.006 |
| Rpl7a | 1.462 | 0.000 | -1.110 | 0.001 |
| E2f2 | 1.592 | 0.000 | -1.121 | 0.006 |
| Slc25a23 | -1.363 | 0.000 | 1.342 | 0.001 |
| C5ar1 | 4.678 | 0.000 | -3.195 | 0.000 |
| AABR07025358.1 | -1.967 | 0.000 | 1.886 | 0.007 |
| LOC108348072 | 1.637 | 0.003 | -1.579 | 0.008 |
| LOC689986 | -1.662 | 0.003 | 2.825 | 0.002 |
| Tcim | 3.860 | 0.001 | -3.404 | 0.007 |
| Garem2 | -2.096 | 0.000 | 1.441 | 0.002 |
| AC128859.3 | 2.614 | 0.000 | -3.569 | 0.000 |
| F2rl3 | 2.678 | 0.000 | -2.179 | 0.008 |
| Nlrc5 | 2.436 | 0.000 | -2.091 | 0.000 |
| AABR07039229.2 | -1.152 | 0.016 | 1.202 | 0.036 |
| Uhrf1 | 3.096 | 0.000 | -1.879 | 0.003 |
| Myo18b | 1.877 | 0.000 | -1.625 | 0.001 |
| Tshz2 | -1.321 | 0.006 | 1.413 | 0.028 |
| Itgb3 | 2.669 | 0.000 | -2.337 | 0.000 |
| AABR07066529.1 | 1.872 | 0.000 | -1.665 | 0.002 |
| Kcne4 | 1.444 | 0.001 | -1.294 | 0.013 |
| Trip6 | 1.433 | 0.000 | -1.188 | 0.000 |
| Il2rb | 4.230 | 0.000 | -3.367 | 0.001 |
| Haspin | 1.828 | 0.000 | -1.253 | 0.012 |
| Pip5kl1 | -1.642 | 0.000 | 1.201 | 0.043 |
| RGD1559482 | 7.250 | 0.000 | -4.318 | 0.001 |
| Gpx1 | 2.044 | 0.000 | -1.206 | 0.002 |
| Sowahc | 1.224 | 0.000 | -1.074 | 0.000 |
| Plin3 | 2.376 | 0.000 | -1.522 | 0.000 |
| Traf1 | 1.691 | 0.002 | -1.701 | 0.008 |
| Smagp | 2.729 | 0.000 | -1.857 | 0.000 |
| LOC688459 | 4.258 | 0.000 | -3.893 | 0.000 |
| Racgap1 | 2.733 | 0.000 | -1.879 | 0.006 |
| Ptrh1 | 1.537 | 0.000 | -1.423 | 0.002 |
| Ntsr2 | -1.636 | 0.000 | 1.519 | 0.000 |
| AC095947.3 | 1.467 | 0.000 | -1.004 | 0.003 |
| Ccr5 | 1.095 | 0.001 | -1.019 | 0.012 |
| Map2k3 | 2.528 | 0.000 | -1.241 | 0.015 |
| AABR07045621.1 | -1.882 | 0.000 | 1.692 | 0.000 |
| Myh9l1 | 1.190 | 0.000 | -1.075 | 0.036 |
| LOC100911837 | -1.547 | 0.043 | 2.623 | 0.000 |
| Oas2 | 1.609 | 0.032 | -1.789 | 0.027 |
| Nat8l | -1.382 | 0.000 | 1.239 | 0.005 |
| Shisa8 | -1.406 | 0.024 | 1.363 | 0.035 |
| Chrm3 | -1.537 | 0.000 | 1.297 | 0.005 |
| LOC108348047 | 3.707 | 0.000 | -3.212 | 0.000 |
| Tnfaip3 | 1.168 | 0.000 | -1.068 | 0.006 |
| AABR07021544.1 | 2.006 | 0.000 | -1.513 | 0.005 |
| Gpr6 | -2.556 | 0.002 | 3.238 | 0.012 |
| AABR07028488.1 | -1.336 | 0.000 | 1.196 | 0.003 |
| Wdr62 | 3.091 | 0.008 | -3.739 | 0.018 |
| Asap3 | 1.462 | 0.000 | -1.016 | 0.011 |
| Adcy9 | -1.138 | 0.000 | 1.010 | 0.021 |
| Csf2ra | 3.255 | 0.000 | -2.422 | 0.000 |
| AABR07060872.1 | 1.580 | 0.013 | -1.770 | 0.004 |
| Lrg1 | 4.268 | 0.000 | -2.182 | 0.000 |
| Sinhcaf | 2.572 | 0.000 | -1.676 | 0.005 |
| Ifi44l | 1.033 | 0.003 | -1.636 | 0.000 |
| AABR07015531.1 | -1.205 | 0.006 | 1.836 | 0.002 |
| Ms4a4e | 5.583 | 0.000 | -3.155 | 0.002 |
| Cdc45 | 2.017 | 0.000 | -1.454 | 0.001 |
| Cenpo | 2.233 | 0.000 | -1.742 | 0.002 |
| Eng | 2.385 | 0.000 | -1.917 | 0.000 |
| RT1-CE10 | 2.663 | 0.001 | -2.698 | 0.002 |
| Nrxn1 | -1.281 | 0.000 | 1.013 | 0.001 |
| MGC105649 | 6.151 | 0.000 | -3.530 | 0.008 |
| Pmepa1 | 1.575 | 0.000 | -1.071 | 0.012 |
| Vav1 | 3.291 | 0.000 | -2.004 | 0.003 |
| AABR07002848.1 | 1.226 | 0.000 | -1.001 | 0.000 |
| Rps27l | 2.350 | 0.000 | -1.432 | 0.000 |
| Dbf4 | 3.662 | 0.000 | -3.457 | 0.000 |
| Fbln5 | 2.315 | 0.000 | -1.806 | 0.000 |
| LOC100362342 | 1.265 | 0.000 | -1.367 | 0.001 |
| Hspa1b | 4.864 | 0.000 | -2.960 | 0.000 |
| Ervfrd-1 | 4.258 | 0.000 | -3.068 | 0.004 |
| Myl4 | 1.352 | 0.015 | -1.130 | 0.038 |
| Ctsz | 3.609 | 0.000 | -1.859 | 0.001 |
| Col25a1 | -2.587 | 0.000 | 1.681 | 0.001 |
| Nrn1 | -1.636 | 0.000 | 1.310 | 0.001 |
| Fpgs | 1.304 | 0.000 | -1.440 | 0.000 |
| Tnfaip6 | 2.815 | 0.000 | -2.205 | 0.000 |
| Cebpd | 3.450 | 0.000 | -1.497 | 0.005 |
| Ptger2 | 2.587 | 0.000 | -2.093 | 0.003 |
| Kctd4 | -2.400 | 0.000 | 1.326 | 0.028 |
| AABR07039303.3 | -3.348 | 0.000 | 3.014 | 0.003 |
| AC114233.2 | 6.001 | 0.000 | -2.996 | 0.016 |
| AABR07038983.1 | 7.080 | 0.000 | -3.826 | 0.000 |
| C2 | 2.145 | 0.000 | -1.762 | 0.000 |
| AABR07059679.1 | -1.220 | 0.000 | 1.120 | 0.008 |
| Mycn | -1.522 | 0.000 | 1.306 | 0.007 |
| AABR07002337.1 | -1.219 | 0.002 | 1.108 | 0.010 |
| AABR07014424.1 | 6.358 | 0.000 | -4.025 | 0.014 |
| 7SK | -1.291 | 0.002 | 1.511 | 0.002 |
| Ddx11 | 1.385 | 0.000 | -1.197 | 0.004 |
| Catsperz | -1.530 | 0.000 | 1.454 | 0.007 |
| AABR07049695.2 | 2.353 | 0.043 | -2.004 | 0.048 |
| AABR07048878.1 | -1.200 | 0.001 | 1.053 | 0.015 |
| Nwd2 | -1.848 | 0.000 | 1.410 | 0.006 |
| Enpep | 1.927 | 0.000 | -1.762 | 0.000 |
| Rbp3 | -3.296 | 0.012 | 4.179 | 0.044 |
| Tes | 1.778 | 0.000 | -1.301 | 0.001 |
| Mmrn2 | 1.348 | 0.000 | -1.045 | 0.007 |
| Plcg2 | 2.153 | 0.000 | -1.496 | 0.000 |
| Gdf10 | -2.678 | 0.000 | 2.091 | 0.000 |
| AABR07004703.1 | -1.302 | 0.001 | 1.092 | 0.038 |
| AABR07010468.1 | -1.248 | 0.001 | 1.307 | 0.002 |
| 7SK | -1.636 | 0.000 | 1.340 | 0.006 |
| Pnma3 | -2.240 | 0.000 | 1.389 | 0.001 |
| AABR07015917.1 | 3.675 | 0.000 | -1.691 | 0.041 |
| Haus8 | 1.662 | 0.000 | -1.109 | 0.001 |
| Parvg | 3.165 | 0.000 | -2.149 | 0.000 |
| Nwd1 | -1.311 | 0.001 | 1.028 | 0.020 |
| AABR07007026.1 | -1.561 | 0.002 | 1.613 | 0.030 |
| Dact3 | -1.323 | 0.000 | 1.115 | 0.001 |
| U6 | -1.969 | 0.000 | 1.620 | 0.013 |
| Fosl2 | 1.027 | 0.000 | -1.059 | 0.005 |
| Bend6 | -1.088 | 0.000 | 1.066 | 0.000 |
| Btk | 2.839 | 0.000 | -1.477 | 0.017 |
| AABR07026984.1 | 3.377 | 0.002 | -2.221 | 0.014 |
| AABR07019254.1 | -3.032 | 0.020 | 2.866 | 0.049 |
| Klrc2 | 4.446 | 0.001 | -2.582 | 0.044 |
| Sox21 | -1.039 | 0.002 | 1.057 | 0.003 |
| LOC102556085 | 2.939 | 0.044 | -3.433 | 0.023 |
| AABR07030791.1 | 4.065 | 0.005 | -5.963 | 0.001 |
| AABR07058464.1 | 1.002 | 0.013 | -1.022 | 0.005 |
| Shcbp1 | 3.252 | 0.000 | -1.725 | 0.015 |
| AABR07001389.1 | 3.580 | 0.000 | -2.635 | 0.002 |
| Scn1a | -1.614 | 0.000 | 1.635 | 0.001 |
| LOC102553088 | -2.484 | 0.000 | 1.527 | 0.021 |
| Faim2 | -1.180 | 0.000 | 1.094 | 0.001 |
| Lilrb3a | 3.902 | 0.000 | -2.988 | 0.001 |
| Efcab11 | 2.319 | 0.000 | -2.051 | 0.002 |
| Bmp7 | 1.644 | 0.002 | -1.683 | 0.003 |
| Adcy10 | -1.658 | 0.028 | 2.085 | 0.007 |
| Slco4a1 | 1.655 | 0.000 | -1.417 | 0.011 |
| AABR07030861.1 | 1.243 | 0.000 | -1.037 | 0.000 |
| AABR07029417.1 | -1.323 | 0.000 | 1.307 | 0.000 |
| Dnajc22 | 3.209 | 0.000 | -1.834 | 0.025 |
| Itga1 | 1.526 | 0.000 | -1.124 | 0.032 |
| AABR07051069.1 | 1.061 | 0.003 | -1.037 | 0.025 |
| Dhh | 1.570 | 0.002 | -1.227 | 0.021 |
| Mdfic | 2.096 | 0.000 | -1.240 | 0.003 |
| Prmt8 | -1.906 | 0.000 | 1.574 | 0.003 |
| LOC103689947 | -2.523 | 0.000 | 2.204 | 0.016 |
| AABR07047189.1 | -3.165 | 0.019 | 3.810 | 0.027 |
| Daam2 | -1.493 | 0.000 | 1.239 | 0.001 |
| P3h1 | 1.203 | 0.000 | -1.109 | 0.001 |
| AABR07005837.1 | 3.964 | 0.002 | -3.977 | 0.005 |
| AABR07005837.2 | 4.193 | 0.000 | -2.181 | 0.033 |
| AABR07070518.1 | -2.449 | 0.000 | 2.289 | 0.010 |
| Xkrx | -2.257 | 0.000 | 2.131 | 0.011 |
| Sim2 | -2.695 | 0.003 | 2.638 | 0.011 |
| Runx3 | 5.499 | 0.000 | -3.193 | 0.005 |
| Il18rap | 3.469 | 0.000 | -1.960 | 0.006 |
| Clec7a | 7.281 | 0.000 | -3.215 | 0.005 |
| Rrm2 | 5.110 | 0.000 | -2.481 | 0.007 |
| SNORA70 | 1.582 | 0.010 | -1.424 | 0.014 |
| Ier2 | 2.042 | 0.000 | -1.731 | 0.000 |
| Vdr | 2.108 | 0.000 | -1.597 | 0.007 |
| Rel | 1.080 | 0.004 | -1.006 | 0.011 |
| Mastl | 3.368 | 0.000 | -2.103 | 0.001 |
| Tnip3 | 4.101 | 0.009 | -4.114 | 0.016 |
| AABR07021357.1 | 4.220 | 0.027 | -3.752 | 0.044 |
| Srpk3 | -1.803 | 0.001 | 1.550 | 0.031 |
| Isg20 | 1.810 | 0.001 | -1.946 | 0.005 |
| Fhod1 | 2.490 | 0.000 | -1.738 | 0.001 |
| AABR07041096.1 | -1.567 | 0.000 | 1.352 | 0.002 |
| AABR07026032.1 | -2.178 | 0.000 | 1.611 | 0.011 |
| Clec12a | 5.519 | 0.000 | -2.082 | 0.017 |
| AC126640.1 | -1.009 | 0.010 | 1.041 | 0.020 |
| Wdr72 | 1.832 | 0.003 | -1.525 | 0.038 |
| Flna | 2.595 | 0.000 | -1.249 | 0.030 |
| Aoah | 4.538 | 0.000 | -2.810 | 0.000 |
| AABR07026483.1 | -1.189 | 0.010 | 1.144 | 0.019 |
| Hilpda | 1.484 | 0.000 | -1.268 | 0.000 |
| Cftr | -2.992 | 0.002 | 2.597 | 0.031 |
| Prtg | -1.850 | 0.003 | 1.619 | 0.018 |
| Phldb3 | 1.771 | 0.001 | -1.952 | 0.001 |
| AABR07013686.1 | -1.888 | 0.000 | 1.409 | 0.018 |
| Kcnc1 | -1.257 | 0.000 | 1.048 | 0.009 |
| Tarm1 | 5.901 | 0.000 | -4.469 | 0.003 |
| Gpx2 | 3.965 | 0.000 | -3.885 | 0.000 |
| Gpr52 | -1.101 | 0.044 | 1.320 | 0.009 |
| Oscar | 4.261 | 0.000 | -2.640 | 0.002 |
| Rasl10b | -2.568 | 0.000 | 1.674 | 0.024 |
| AABR07064980.1 | -2.318 | 0.001 | 1.440 | 0.044 |
| AC103574.1 | 3.347 | 0.037 | -3.843 | 0.039 |
| AABR07062138.2 | 5.346 | 0.000 | -2.910 | 0.003 |
| Bnip2 | 1.461 | 0.000 | -1.139 | 0.000 |
| Kif11 | 3.532 | 0.000 | -1.932 | 0.021 |
| Hephl1 | 3.763 | 0.000 | -2.233 | 0.001 |
| AABR07007642.1 | -2.087 | 0.000 | 1.373 | 0.011 |
| Tent5b | 2.942 | 0.000 | -3.098 | 0.000 |
| Exo1 | 3.965 | 0.000 | -1.941 | 0.036 |
| Casp7 | 1.366 | 0.000 | -1.102 | 0.000 |
| Olr1 | 5.277 | 0.000 | -3.447 | 0.000 |
| AC099449.1 | 2.293 | 0.000 | -1.321 | 0.024 |
| Olfml1 | -1.438 | 0.000 | 1.381 | 0.000 |
| AABR07030086.2 | 1.713 | 0.001 | -1.907 | 0.003 |
| Y_RNA | 4.132 | 0.001 | -3.679 | 0.010 |
| Unc13c | -1.390 | 0.008 | 1.588 | 0.047 |
| AABR07036336.2 | -2.477 | 0.005 | 2.424 | 0.003 |
| Piezo1 | 2.774 | 0.000 | -2.023 | 0.000 |
| Muc6 | -1.559 | 0.003 | 1.397 | 0.012 |
| AABR07025272.1 | 2.345 | 0.022 | -3.344 | 0.011 |
| Cd300a | 3.358 | 0.000 | -2.459 | 0.000 |
| Slfn4 | 4.569 | 0.000 | -4.139 | 0.000 |
| AABR07006097.1 | -2.203 | 0.000 | 1.849 | 0.005 |
| RGD1561730 | 6.387 | 0.000 | -5.916 | 0.002 |
| Pla1a | 3.777 | 0.000 | -1.822 | 0.000 |
| AABR07027407.1 | -1.410 | 0.000 | 1.144 | 0.018 |
| LOC103690326 | 4.248 | 0.001 | -4.263 | 0.003 |
| Kcnh3 | -1.116 | 0.000 | 1.046 | 0.004 |
| Cebpb | 2.616 | 0.000 | -2.256 | 0.000 |
| Slc47a1 | 2.103 | 0.010 | -2.807 | 0.000 |
| Nos2 | 3.013 | 0.010 | -3.841 | 0.003 |
| Itga5 | 4.338 | 0.000 | -2.819 | 0.000 |
| LOC103691157 | 4.241 | 0.001 | -2.294 | 0.044 |
| Fam81a | -1.749 | 0.000 | 1.418 | 0.000 |
| Ahnak | 1.439 | 0.000 | -1.023 | 0.010 |
| AC133613.1 | -1.893 | 0.000 | 1.374 | 0.040 |
| AABR07027212.1 | -1.125 | 0.000 | 1.168 | 0.000 |
| LOC100909671 | 4.331 | 0.014 | -4.831 | 0.021 |
| Ccdc148 | -1.240 | 0.007 | 1.274 | 0.003 |
| Stil | 1.521 | 0.000 | -1.123 | 0.035 |
| Rnf125 | 2.596 | 0.000 | -1.692 | 0.015 |
| AABR07022653.1 | -4.034 | 0.001 | 3.380 | 0.045 |
| Phactr3 | -1.413 | 0.000 | 1.286 | 0.000 |
| AABR07013914.1 | -3.843 | 0.004 | 3.327 | 0.048 |
| AABR07018244.2 | 3.551 | 0.000 | -2.905 | 0.000 |
| Lilrc2 | 6.201 | 0.000 | -3.669 | 0.000 |
| AC118094.1 | -3.484 | 0.018 | 4.453 | 0.011 |
| Errfi1 | 1.493 | 0.000 | -1.218 | 0.000 |
| Tcf19 | 2.504 | 0.000 | -1.433 | 0.008 |
| Asic2 | -1.695 | 0.000 | 1.223 | 0.001 |
| Pnck | -2.271 | 0.000 | 1.588 | 0.003 |
| Prrx2 | 1.888 | 0.002 | -1.712 | 0.019 |
| Plcb2 | 1.158 | 0.000 | -1.129 | 0.001 |
| AABR07051515.1 | 2.404 | 0.000 | -1.720 | 0.027 |
| Zfp36 | 3.097 | 0.000 | -2.069 | 0.000 |
| Il7r | 1.093 | 0.004 | -1.159 | 0.008 |
| Ccnb1 | 2.591 | 0.000 | -1.563 | 0.010 |
| 7SK | 3.288 | 0.001 | -2.711 | 0.008 |
| Dhrs9 | 2.442 | 0.000 | -1.541 | 0.019 |
| Tnc | 3.251 | 0.000 | -2.468 | 0.001 |
| Tmem52b | 3.510 | 0.000 | -2.111 | 0.001 |
| AABR07044635.1 | 2.997 | 0.030 | -3.011 | 0.032 |
| Igfbp1 | 1.641 | 0.030 | -2.026 | 0.005 |
| Camkv | -1.629 | 0.000 | 1.117 | 0.039 |
| Rab11fip1 | 1.498 | 0.005 | -1.793 | 0.003 |
| LOC690097 | 4.702 | 0.000 | -2.837 | 0.004 |
| AABR07060519.1 | -1.055 | 0.003 | 1.068 | 0.009 |
| AABR07026032.3 | -1.995 | 0.000 | 1.557 | 0.007 |
| AABR07025140.1 | 1.844 | 0.000 | -2.176 | 0.000 |
| Myo1g | 4.561 | 0.000 | -2.823 | 0.000 |
| Oas3 | 1.829 | 0.026 | -3.382 | 0.001 |
| Rad51b | 2.691 | 0.000 | -1.155 | 0.029 |
| AABR07053509.2 | -2.152 | 0.001 | 1.792 | 0.041 |
| AC119015.4 | 1.646 | 0.000 | -1.144 | 0.009 |
| Has3 | -1.839 | 0.000 | 1.698 | 0.004 |
| B4galt1 | 2.449 | 0.000 | -1.640 | 0.002 |
| Lamc3 | -2.191 | 0.000 | 1.038 | 0.002 |
| Clec2g | 1.709 | 0.000 | -1.346 | 0.000 |
| AABR07021998.1 | 5.354 | 0.000 | -3.738 | 0.017 |
| Scarf1 | 1.366 | 0.000 | -1.167 | 0.005 |
| RGD1560281 | 5.347 | 0.026 | -5.354 | 0.040 |
| AABR07007878.1 | -2.239 | 0.011 | 2.682 | 0.007 |
| AC141220.1 | 4.133 | 0.010 | -4.148 | 0.017 |
| Hmmr | 1.761 | 0.000 | -1.180 | 0.003 |
| Bst2 | 2.265 | 0.000 | -1.927 | 0.000 |
| Sdc1 | 4.957 | 0.000 | -2.832 | 0.011 |
| Bcl6b | 1.067 | 0.018 | -1.326 | 0.006 |
| AABR07071838.1 | 4.635 | 0.003 | -4.166 | 0.009 |
| Brip1 | 1.585 | 0.001 | -1.375 | 0.033 |
| Gm27820 | -2.290 | 0.003 | 1.795 | 0.019 |
| Sowaha | -2.074 | 0.000 | 1.726 | 0.001 |
| Knl1 | 2.986 | 0.000 | -1.626 | 0.011 |
| Astn2 | -1.349 | 0.000 | 1.067 | 0.004 |
| Kifc2 | -1.376 | 0.000 | 1.203 | 0.002 |
| Prr29 | 1.465 | 0.000 | -1.359 | 0.004 |
| AABR07051882.2 | -1.681 | 0.000 | 1.440 | 0.000 |
| AABR07044837.2 | 4.004 | 0.000 | -3.174 | 0.000 |
| LOC497796 | 4.162 | 0.003 | -3.697 | 0.011 |
| Kif15 | 2.399 | 0.000 | -1.417 | 0.025 |
| Col15a1 | 3.008 | 0.000 | -2.726 | 0.000 |
| AABR07017159.1 | -1.893 | 0.013 | 1.796 | 0.017 |
| Kcnq4 | -2.424 | 0.000 | 1.270 | 0.012 |
| AABR07011857.2 | -2.029 | 0.000 | 1.977 | 0.010 |
| Slc10a3 | 1.429 | 0.000 | -1.123 | 0.004 |
| AABR07071208.2 | 2.628 | 0.003 | -2.373 | 0.003 |
| Pxdn | 1.771 | 0.000 | -1.780 | 0.001 |
| AABR07027309.1 | -1.321 | 0.000 | 1.251 | 0.014 |
| Slc24a3 | -1.765 | 0.000 | 1.021 | 0.020 |
| Adam17 | 1.265 | 0.000 | -1.091 | 0.001 |
| Nlrp12 | 5.366 | 0.001 | -3.032 | 0.040 |
| Esyt1 | 2.168 | 0.000 | -1.634 | 0.000 |
| AC125248.1 | 5.143 | 0.000 | -1.932 | 0.011 |
| AABR07059527.1 | -1.971 | 0.018 | 2.393 | 0.011 |
| Fancd2 | 2.162 | 0.000 | -1.312 | 0.010 |
| LOC103694210 | -1.988 | 0.001 | 1.516 | 0.048 |
| AABR07027494.1 | 1.609 | 0.003 | -1.537 | 0.005 |
| Atp2b3 | -1.680 | 0.000 | 1.061 | 0.001 |
| Ulbp1 | 5.146 | 0.000 | -2.423 | 0.025 |
| LOC690414 | -2.514 | 0.000 | 1.716 | 0.030 |
| AABR07054490.1 | 2.714 | 0.000 | -2.497 | 0.000 |
| Clec4e | 8.118 | 0.000 | -4.383 | 0.000 |
| AABR07039446.2 | 3.408 | 0.000 | -2.543 | 0.000 |
| AABR07030914.1 | -3.474 | 0.000 | 3.573 | 0.002 |
| AABR07068214.1 | 1.687 | 0.000 | -1.450 | 0.007 |
| AABR07033720.1 | -1.755 | 0.000 | 1.092 | 0.014 |
| Rsph6a | -1.468 | 0.000 | 1.294 | 0.010 |
| Tfec | 5.112 | 0.000 | -2.788 | 0.001 |
| AABR07062069.2 | -3.713 | 0.004 | 3.657 | 0.007 |
| SNORA73 | 1.096 | 0.023 | -1.217 | 0.046 |
| Klrk1 | 3.266 | 0.000 | -2.006 | 0.009 |
| AABR07006736.1 | -3.119 | 0.032 | 3.088 | 0.025 |
| AABR07021812.1 | -1.369 | 0.000 | 1.071 | 0.001 |
| Ly6c | 6.725 | 0.000 | -4.813 | 0.000 |
| Mgst2 | 4.899 | 0.000 | -1.951 | 0.001 |
| LOC690045 | 5.301 | 0.000 | -2.879 | 0.000 |
| Igfbp3 | 4.045 | 0.000 | -3.239 | 0.000 |
| AABR07065173.1 | -2.899 | 0.001 | 2.801 | 0.003 |
| AC115202.3 | 5.261 | 0.000 | -4.312 | 0.000 |
| AABR07049773.2 | -2.150 | 0.004 | 1.843 | 0.027 |
| Ace | 1.539 | 0.000 | -1.358 | 0.002 |
| Slc18a3 | 3.875 | 0.000 | -2.266 | 0.003 |
| AABR07035955.1 | 4.075 | 0.000 | -2.112 | 0.011 |
| AABR07058158.1 | 1.825 | 0.000 | -1.580 | 0.001 |
| Il1rapl2 | -1.507 | 0.015 | 1.556 | 0.013 |
| AABR07072853.5 | 3.787 | 0.000 | -1.798 | 0.015 |
| AABR07044631.2 | 1.669 | 0.000 | -1.026 | 0.040 |
| AABR07012058.2 | -3.517 | 0.004 | 2.983 | 0.039 |
| novel.1006 | 1.534 | 0.000 | -1.069 | 0.011 |
| novel.101 | -1.225 | 0.040 | 1.813 | 0.048 |
| novel.1041 | 2.198 | 0.000 | -1.989 | 0.000 |
| novel.1048 | 2.091 | 0.000 | -1.428 | 0.017 |
| novel.1051 | -1.809 | 0.002 | 1.644 | 0.007 |
| novel.1062 | 5.115 | 0.000 | -2.979 | 0.002 |
| novel.1073 | 3.880 | 0.000 | -2.291 | 0.002 |
| novel.1087 | 3.342 | 0.000 | -2.834 | 0.016 |
| novel.1092 | -2.023 | 0.000 | 1.172 | 0.050 |
| novel.1099 | 5.961 | 0.000 | -2.087 | 0.048 |
| novel.1100 | 3.763 | 0.000 | -2.325 | 0.007 |
| novel.1102 | -1.371 | 0.003 | 1.242 | 0.029 |
| novel.1111 | 4.369 | 0.002 | -3.917 | 0.012 |
| novel.1120 | -3.950 | 0.000 | 2.920 | 0.000 |
| novel.1133 | -1.779 | 0.000 | 1.814 | 0.001 |
| novel.1134 | -1.620 | 0.000 | 1.067 | 0.006 |
| novel.1135 | -1.354 | 0.000 | 1.210 | 0.000 |
| novel.1137 | -1.096 | 0.031 | 1.113 | 0.032 |
| novel.1147 | -3.348 | 0.000 | 2.443 | 0.009 |
| novel.1154 | 3.968 | 0.000 | -2.370 | 0.009 |
| novel.1197 | 1.561 | 0.000 | -1.086 | 0.045 |
| novel.1198 | -1.557 | 0.000 | 1.070 | 0.001 |
| novel.1232 | -1.906 | 0.000 | 1.200 | 0.004 |
| novel.124 | -1.466 | 0.000 | 1.406 | 0.005 |
| novel.1244 | -2.823 | 0.000 | 2.473 | 0.017 |
| novel.1256 | -1.399 | 0.001 | 1.034 | 0.042 |
| novel.1257 | -2.448 | 0.000 | 2.248 | 0.041 |
| novel.1264 | -1.197 | 0.000 | 1.084 | 0.002 |
| novel.1267 | -1.240 | 0.001 | 1.060 | 0.012 |
| novel.1278 | 2.638 | 0.017 | -3.088 | 0.017 |
| novel.1280 | 1.951 | 0.001 | -1.526 | 0.047 |
| novel.1283 | -1.516 | 0.000 | 1.062 | 0.030 |
| novel.1288 | -1.497 | 0.003 | 1.749 | 0.003 |
| novel.1291 | -1.322 | 0.000 | 1.316 | 0.000 |
| novel.1300 | 2.997 | 0.000 | -1.939 | 0.042 |
| novel.1306 | 7.337 | 0.000 | -3.139 | 0.006 |
| novel.1309 | 5.602 | 0.000 | -3.301 | 0.000 |
| novel.1310 | 4.776 | 0.000 | -2.104 | 0.028 |
| novel.1317 | 1.291 | 0.008 | -1.255 | 0.021 |
| novel.1318 | 1.037 | 0.018 | -1.056 | 0.020 |
| novel.132 | 5.875 | 0.000 | -4.048 | 0.000 |
| novel.1321 | -1.551 | 0.000 | 1.388 | 0.006 |
| novel.1326 | -2.093 | 0.021 | 1.996 | 0.041 |
| novel.1329 | -1.418 | 0.000 | 1.661 | 0.004 |
| novel.1333 | -1.796 | 0.000 | 1.558 | 0.000 |
| novel.1366 | 2.835 | 0.006 | -2.237 | 0.041 |
| novel.1371 | -2.435 | 0.023 | 3.141 | 0.039 |
| novel.14 | -1.505 | 0.000 | 1.511 | 0.003 |
| novel.1438 | -1.748 | 0.000 | 1.215 | 0.018 |
| novel.1451 | 2.855 | 0.000 | -1.859 | 0.000 |
| novel.1468 | -1.133 | 0.000 | 1.065 | 0.001 |
| novel.152 | -1.755 | 0.000 | 1.641 | 0.003 |
| novel.161 | -1.628 | 0.001 | 1.235 | 0.045 |
| novel.163 | -1.326 | 0.000 | 1.208 | 0.001 |
| novel.165 | -2.756 | 0.000 | 2.044 | 0.000 |
| novel.176 | 1.681 | 0.000 | -1.250 | 0.003 |
| novel.182 | 2.286 | 0.000 | -1.540 | 0.000 |
| novel.187 | -3.055 | 0.001 | 2.345 | 0.028 |
| novel.198 | -1.219 | 0.002 | 1.428 | 0.001 |
| novel.208 | -1.389 | 0.000 | 1.072 | 0.007 |
| novel.21 | 1.843 | 0.014 | -1.559 | 0.045 |
| novel.235 | 1.629 | 0.000 | -1.281 | 0.001 |
| novel.238 | 2.957 | 0.000 | -2.646 | 0.000 |
| novel.26 | 4.272 | 0.000 | -2.488 | 0.006 |
| novel.269 | -2.052 | 0.000 | 1.750 | 0.004 |
| novel.292 | 4.238 | 0.030 | -4.249 | 0.044 |
| novel.30 | -1.289 | 0.000 | 1.111 | 0.003 |
| novel.307 | -1.116 | 0.005 | 1.281 | 0.003 |
| novel.320 | 2.530 | 0.007 | -1.991 | 0.031 |
| novel.322 | 5.186 | 0.000 | -4.238 | 0.002 |
| novel.349 | -1.992 | 0.000 | 2.470 | 0.000 |
| novel.363 | -1.319 | 0.000 | 1.093 | 0.021 |
| novel.365 | 1.674 | 0.000 | -1.143 | 0.010 |
| novel.376 | -2.231 | 0.001 | 1.951 | 0.046 |
| novel.38 | 4.977 | 0.000 | -4.500 | 0.000 |
| novel.386 | 2.504 | 0.000 | -2.676 | 0.003 |
| novel.391 | 3.655 | 0.000 | -2.757 | 0.000 |
| novel.410 | -1.137 | 0.000 | 1.040 | 0.000 |
| novel.418 | 4.028 | 0.010 | -3.652 | 0.008 |
| novel.421 | -1.069 | 0.004 | 1.177 | 0.011 |
| novel.427 | -1.774 | 0.000 | 1.876 | 0.002 |
| novel.444 | 7.972 | 0.000 | -2.999 | 0.000 |
| novel.446 | -2.461 | 0.004 | 2.366 | 0.009 |
| novel.448 | -1.507 | 0.000 | 1.023 | 0.000 |
| novel.481 | -1.778 | 0.000 | 1.538 | 0.000 |
| novel.488 | 1.623 | 0.003 | -1.763 | 0.005 |
| novel.492 | -1.745 | 0.000 | 1.250 | 0.026 |
| novel.495 | 3.828 | 0.000 | -2.375 | 0.002 |
| novel.498 | -2.479 | 0.000 | 1.887 | 0.000 |
| novel.5 | 2.254 | 0.000 | -1.402 | 0.047 |
| novel.512 | 1.749 | 0.023 | -2.056 | 0.042 |
| novel.518 | 1.139 | 0.001 | -1.068 | 0.026 |
| novel.522 | -1.285 | 0.000 | 1.012 | 0.006 |
| novel.537 | -1.344 | 0.000 | 1.057 | 0.002 |
| novel.541 | 1.635 | 0.000 | -1.452 | 0.024 |
| novel.548 | -1.017 | 0.001 | 1.302 | 0.003 |
| novel.577 | -1.276 | 0.001 | 1.240 | 0.009 |
| novel.59 | 2.196 | 0.000 | -1.190 | 0.021 |
| novel.616 | -1.430 | 0.000 | 1.269 | 0.024 |
| novel.643 | -1.423 | 0.000 | 1.085 | 0.000 |
| novel.649 | 5.552 | 0.000 | -4.620 | 0.001 |
| novel.652 | -1.404 | 0.000 | 1.294 | 0.000 |
| novel.657 | -1.026 | 0.007 | 1.095 | 0.004 |
| novel.658 | -3.594 | 0.000 | 2.980 | 0.000 |
| novel.673 | -1.002 | 0.000 | 1.089 | 0.015 |
| novel.678 | -1.143 | 0.001 | 1.077 | 0.025 |
| novel.683 | -1.239 | 0.000 | 1.110 | 0.001 |
| novel.699 | -1.274 | 0.000 | 1.117 | 0.021 |
| novel.702 | -1.343 | 0.000 | 1.368 | 0.001 |
| novel.705 | 3.897 | 0.000 | -2.716 | 0.009 |
| novel.708 | -1.661 | 0.000 | 1.467 | 0.000 |
| novel.711 | -1.749 | 0.000 | 1.267 | 0.011 |
| novel.718 | -1.606 | 0.000 | 1.026 | 0.017 |
| novel.720 | 4.789 | 0.000 | -2.908 | 0.014 |
| novel.723 | 3.073 | 0.004 | -3.254 | 0.011 |
| novel.726 | 6.468 | 0.000 | -2.853 | 0.004 |
| novel.731 | 6.395 | 0.000 | -4.505 | 0.001 |
| novel.735 | -1.586 | 0.000 | 1.569 | 0.002 |
| novel.741 | -1.277 | 0.018 | 1.325 | 0.033 |
| novel.748 | 5.199 | 0.000 | -3.479 | 0.000 |
| novel.750 | -1.456 | 0.005 | 1.288 | 0.031 |
| novel.760 | 3.597 | 0.000 | -2.038 | 0.005 |
| novel.763 | 1.563 | 0.001 | -1.409 | 0.005 |
| novel.764 | -2.687 | 0.001 | 2.570 | 0.001 |
| novel.766 | -1.498 | 0.004 | 1.267 | 0.046 |
| novel.770 | 1.903 | 0.005 | -1.678 | 0.008 |
| novel.778 | -1.860 | 0.000 | 1.439 | 0.005 |
| novel.787 | -1.505 | 0.025 | 2.406 | 0.008 |
| novel.797 | 1.757 | 0.001 | -1.520 | 0.039 |
| novel.803 | -3.145 | 0.000 | 2.740 | 0.000 |
| novel.820 | -2.366 | 0.000 | 1.951 | 0.001 |
| novel.827 | -3.181 | 0.001 | 2.364 | 0.036 |
| novel.840 | -2.933 | 0.000 | 3.024 | 0.002 |
| novel.843 | 4.044 | 0.000 | -2.166 | 0.019 |
| novel.852 | 1.018 | 0.010 | -1.097 | 0.032 |
| novel.855 | -2.774 | 0.000 | 1.378 | 0.003 |
| novel.867 | 1.458 | 0.002 | -1.480 | 0.005 |
| novel.870 | -1.425 | 0.000 | 1.719 | 0.000 |
| novel.872 | 1.954 | 0.001 | -1.564 | 0.019 |
| novel.873 | -1.499 | 0.000 | 1.071 | 0.009 |
| novel.876 | -2.462 | 0.000 | 1.494 | 0.016 |
| novel.897 | -1.780 | 0.037 | 2.675 | 0.002 |
| novel.898 | 1.971 | 0.000 | -1.352 | 0.001 |
| novel.900 | -1.483 | 0.000 | 1.197 | 0.001 |
| novel.909 | 1.016 | 0.019 | -1.059 | 0.020 |
| novel.92 | -3.873 | 0.001 | 4.515 | 0.004 |
| novel.922 | 2.070 | 0.000 | -1.481 | 0.002 |
| novel.929 | 4.360 | 0.000 | -2.209 | 0.000 |
| novel.930 | -1.647 | 0.000 | 1.316 | 0.003 |
| novel.935 | -1.201 | 0.021 | 1.248 | 0.038 |
| novel.936 | -3.254 | 0.000 | 3.017 | 0.005 |
| novel.949 | -1.554 | 0.000 | 1.186 | 0.001 |
| novel.954 | -1.967 | 0.000 | 1.363 | 0.018 |
| novel.955 | 1.990 | 0.007 | -2.161 | 0.012 |
| novel.970 | 1.418 | 0.000 | -1.197 | 0.002 |
| novel.974 | 2.659 | 0.000 | -1.754 | 0.006 |
| novel.987 | 4.632 | 0.001 | -3.683 | 0.012 |
| novel.988 | 2.581 | 0.000 | -1.811 | 0.004 |

**Table S4 Statistical analysis of fluorescence intensity in rat brain tissues among groups in Figure 4G**

| **Group** | **Sham** | **CIRI** | **H-PD** |
| --- | --- | --- | --- |
| Sample-1 | 0.105 | 0.304 | 0.253 |
| Sample-2 | 0.116 | 0.354 | 0.264 |
| Sample-3 | 0.135 | 0.395 | 0.289 |
| Mean | 0.119 | 0.351 | 0.269 |
| SD | 0.015 | 0.046 | 0.018 |
| *p* (Shapiro-Wilk) | 0.7082 | 0.8911 | 0.5782 |
| *p* (Levene) | 0.3078 | | |
| *p*-value | CIRI *vs.* Sham | 0.0002 | |
|  | H-PD *vs.* CIRI | 0.0337 | |

Group differences were evaluated using one-way ANOVA followed by Tukey's post hoc test.
